# Temporal segregation of biosynthetic processes is responsible for metabolic oscillations during the budding yeast cell cycle

**DOI:** 10.1038/s42255-023-00741-x

**Published:** 2023-02-27

**Authors:** Vakil Takhaveev, Serdar Özsezen, Edward N. Smith, Andre Zylstra, Marten L. Chaillet, Haoqi Chen, Alexandros Papagiannakis, Andreas Milias-Argeitis, Matthias Heinemann

**Affiliations:** 1 Molecular Systems Biology, Groningen Biomolecular Sciences and Biotechnology Institute, University of Groningen, Groningen, The Netherlands; 2 Present Address: Department of Health Sciences and Technology, ETH Zurich, Zurich, Switzerland; 3 Present Address: Department of Microbiology and Systems Biology, The Netherlands Organization for Applied Scientific Research (TNO), Leiden, The Netherlands; 4 Present Address: Structural Biochemistry, Bijvoet Center for Biomolecular Research, Utrecht University, Utrecht, The Netherlands; 5 Present Address: Department of Biology and Sarafan Chemistry, Engineering, and Medicine for Human Health Institute, Stanford University, Stanford, CA USA

**Keywords:** Metabolism, Single-cell imaging, Wide-field fluorescence microscopy, Cell growth, Fungal systems biology

## Abstract

Many cell biological and biochemical mechanisms controlling the fundamental process of eukaryotic cell division have been identified; however, the temporal dynamics of biosynthetic processes during the cell division cycle are still elusive. Here, we show that key biosynthetic processes are temporally segregated along the cell cycle. Using budding yeast as a model and single-cell methods to dynamically measure metabolic activity, we observe two peaks in protein synthesis, in the G1 and S/G2/M phase, whereas lipid and polysaccharide synthesis peaks only once, during the S/G2/M phase. Integrating the inferred biosynthetic rates into a thermodynamic-stoichiometric metabolic model, we find that this temporal segregation in biosynthetic processes causes flux changes in primary metabolism, with an acceleration of glucose-uptake flux in G1 and phase-shifted oscillations of oxygen and carbon dioxide exchanges. Through experimental validation of the model predictions, we demonstrate that primary metabolism oscillates with cell-cycle periodicity to satisfy the changing demands of biosynthetic processes exhibiting unexpected dynamics during the cell cycle.

## Abstract (web summary)

Takhaveev et al. use single-cell methods and mathematical modeling to reveal distinct waves in the synthesis rates of proteins, lipids and polysaccharides during the budding yeast cell cycle and relate them to flux changes in primary metabolism.

## Main

Cell growth and division are fundamental biological processes. While we have a solid account of the cell biological and biochemical mechanisms controlling the cell division cycle, we know much less about the temporal dynamics of biosynthesis and primary metabolism that drive cell growth during the cell cycle. Whereas DNA biosynthesis is known to be temporally constrained within the S phase, the dynamics of other major biosynthetic processes, such as protein and lipid syntheses, remain unclear; are biosynthetic processes constantly active throughout the whole cell cycle? If their activities change, do the rates of different biosynthetic processes alter in the same manner? Such knowledge is essential to uncover the mechanisms behind cell-growth regulation, whose defects are associated with disease^1,2^.

Currently, protein synthesis is considered to increase with either exponential or constant rate throughout the yeast cell cycle, as determined by population-level studies with radioactive labeling^3–5^ and single-cell analyses^6,7^. Recently, however, we found that the production rate of green fluorescent protein (GFP) controlled by the endogenous *TEF1* promoter peaks in G1 (ref. ^8^), suggesting that protein biosynthetic activity could actually be non-monotonic during the cell cycle. This finding would be consistent with an observed peak of ribosomal protein abundance in G1 (ref. ^9^), although others have found no such dynamics^10^. Likewise, the expression of genes associated with ribosome biogenesis and translation has also been observed peaking in G1 (refs. ^10,11^); however, single-cell RNA-sequencing (RNA-seq) studies have reported either only a small increase of ribosomal protein mRNA in G1 (ref. ^12^) or no notable differences over the cell cycle^13^. As for other macromolecule classes, such as lipids and nucleic acids, their biosynthesis has also been suggested to accelerate during certain phases of the cell cycle according to recent multi-omic studies^9,10^. Yet, the molecular abundances measured in these studies provide only indirect evidence for actual biosynthetic rates. Thus, the temporal dynamics of biosynthetic activities during the cell cycle are still largely elusive. Answering this question will likely require the measurement of rates in a dynamic, cell-cycle-resolved manner, which so far poses enormous technical challenges.

Here, using budding yeast as a model and employing dynamic single-cell fluorescence microscopy with a new stop-and-respond method, we discovered that the activities of protein, lipid and polysaccharide biosynthesis are neither exponential nor constant during the cell cycle. Specifically, we found that protein biosynthesis exhibits two waves of activity per cell cycle, whereas the activities of lipid and polysaccharide biosynthesis are low during the first wave of protein biosynthesis in G1 but high during the second wave in S/G2/M. We converted the discovered patterns of biosynthetic activities into absolute units via a mathematical model of cell-mass dynamics, integrated them into a thermodynamic-stoichiometric metabolic model and thereby inferred the cell-cycle dynamics of the primary metabolic fluxes. As we could experimentally validate the inferred metabolic flux changes, this provided additional evidence for the discovered dynamic patterns of the biosynthetic activities and also allowed us to conclude that the temporal segregation in the biosynthetic processes must be responsible for the hour-scale oscillations in primary metabolism. Our work shows that cell growth during the cell cycle is an aggregate of temporally segregated biosynthetic and primary metabolic processes, which provides fundamental insights into the very basics of cellular physiology.

## Results

### Biosynthesis of macromolecules is temporally segregated

**Extended Data Fig. 1:**
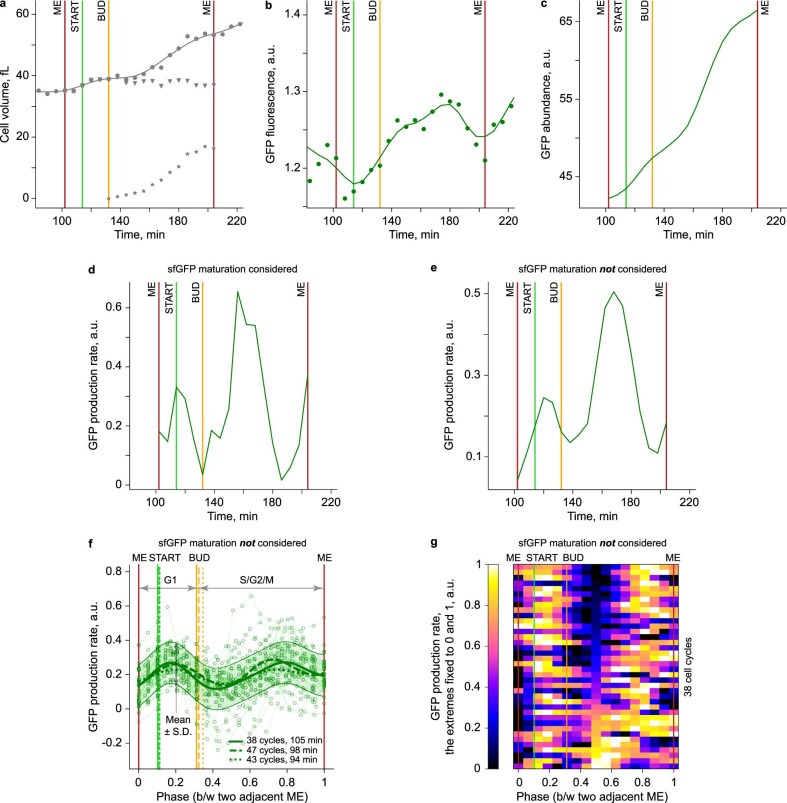
Processing raw single-cell data: smoothing single-cell traces of volume and sfGFP fluorescence, obtaining the production rate of sfGFP expressed from the heterologous promoter tetO_7_, with and without accounting for sfGFP maturation. **(a,b**) Single-cell volume (a) and sfGFP fluorescence (b) during an individual cell cycle. We show raw data with asterisks when corresponding to the daughter cell, triangles when corresponding to the mother cell and circles when corresponding to the whole cell. The vertical lines denote major cell cycle events: mitotic exit (ME), START and budding (BUD). The curves show the LOWESS smoothing with adjacent smoothed values (at the same time points as the raw data) connected with a line. a: the discontinuity in the values that should immediately follow mitotic exits due to cytokinesis is tackled by vertically moving down the data before the first mitotic exit and up the data after the second mitotic exit. b: we measured sfGFP fluorescence only in the mother cell and assumed that it is the same in the whole cell. (**c–e**) Single-cell sfGFP abundance (c), sfGFP production rate calculated with (d) and without (e) accounting for the maturation of the fluorescent protein (assumed maturation half-time 6 minutes, first-order maturation kinetics). The curves show linearly connected values corresponding to the same time points as the raw data. c: values are the product of the smoothed cell volume (a) and smoothed sfGFP fluorescence (b). d: values are a linear combination of the first and second derivatives of sfGFP abundance (c). e: values are the first derivative of sfGFP abundance (c). (**f, g**) The production rate of sfGFP presented as a summary of 38 individual cell-cycle traces. The sfGFP production rate was computed without accounting for the maturation of this fluorescent protein. Values from (e) and other 37 cell cycles correspond to the markers. The plots were built analogously to Fig. 1a, b.

To determine the activity of protein biosynthesis during the cell cycle, we expressed superfolder GFP (sfGFP) from a heterologous, and hence unregulated, promoter (*tet*O_7_) such that sfGFP production solely depends on the activity of the protein biosynthesis machinery. We recorded sfGFP fluorescence intensity and cell volume over time in single cells growing in a microfluidic device^14,15^ and derived the production rate of sfGFP via a mathematical model assuming first-order kinetics of sfGFP maturation (Extended Data Fig. 1a–d). To define cell-cycle phases, we used the nuclear entry of mCherry-labeled Whi5 to denote mitotic exit (beginning of G1) and the subsequent Whi5 re-localization to the cytoplasm to indicate START, as conducted previously^16,17^. We used the moment of bud emergence to demark the beginning of S phase^18,19^.

**Fig. 1:**
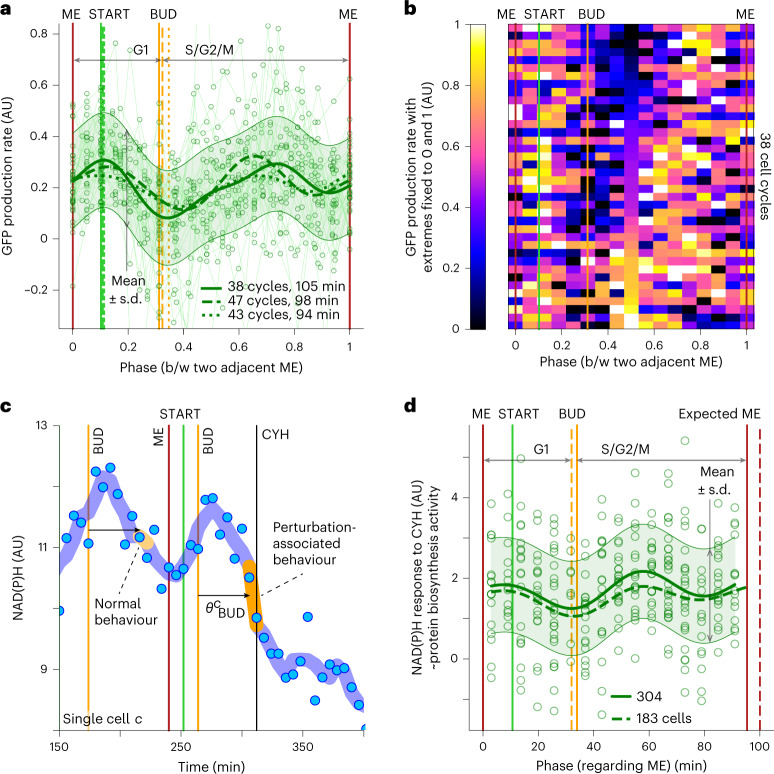
Protein biosynthesis has two activity waves during the cell cycle. **a**,**b**, Heterologous promoter (*tetO*_*7*_)-expressed sfGFP production rate computed from dynamic single-cell fluorescence and volume measurements, incorporating sfGFP maturation (Extended Data Fig. 1a–d). Cell-cycle traces (line-connected markers) of sfGFP production rate are summarized by posterior mean (thick solid curve) and region of high posterior probability (shaded area, mean ± s.d.) of a Gaussian process regression model with a radial basis function (RBF) kernel (**a**). AU, arbitrary units; b/w, between. Dashed and dotted thick curves indicate posterior means obtained via the same data analysis pipeline in two additional replicate experiments (number and average duration of analyzed cell cycles indicated). To align cell-cycle traces and calculate cell-cycle phases, we used as reference points mitotic exit (ME), START, budding (BUD) and next ME, whose average timing in three replicates is indicated. sfGFP production rate in cell cycles presented separately (**b**). Cell-cycle traces from the first replicate in **a** were interpolated, sampled at 17 evenly spaced phase points and min–max normalized. **c**, Measuring protein biosynthesis activity with the stop-and-respond method by determining single-cell NAD(P)H response to CYH, which is the difference between NAD(P)H derivative upon CYH addition and the median NAD(P)H derivative at the same phase ($\theta _{\mathrm{BUD}}^c$) in the unperturbed condition. In CYH experiments, this difference is multiplied by −1 so that metabolic response is on average non-negative. Markers indicate raw mother-cell NAD(P)H fluorescence; curve indicates smoothing (Savitzky–Golay filter). **d**, NAD(P)H response to CYH has two peaks during the cell cycle. Markers indicate single cells analyzed as in **c** from one replicate experiment. Solid curve and shaded area indicate posterior mean and region of high posterior probability (mean ± s.d.) of a Gaussian process regression summarizing the marker values via an RBF kernel. Dashed curve indicates posterior mean obtained via the same data analysis pipeline in the second replicate experiment (number of analyzed cells indicated). Vertical lines indicate mean phases of ME, START and BUD in two replicate experiments. The phase of expected ME is the mean cell-cycle duration before CYH addition. We analyzed cells that had produced at least two buds before the perturbation (not newborn cells).

Here, we found that the production rate of sfGFP exhibits a two-wave behavior during the cell cycle (Fig. 1a and Extended Data Fig. 1d–f). The first peak occurs around START, similar to what we recently found with the endogenous *TEF1* promoter^8^. The sfGFP production rate reaches a minimum around budding, rises to a second peak in the middle of S/G2/M and displays a further minimum just before mitotic exit. By scrutinizing individual cell-cycle traces, we confirmed that both waves of increased protein biosynthesis appear in the majority of cell cycles, instead of arising from separate cell subpopulations, and noticed that the timing of the second peak is more variable compared to the timing of the first (Fig. 1b and Extended Data Fig. 1g). Thus, the production rate of a heterologous promoter-controlled fluorescent protein, reflecting protein biosynthesis activity, has two waves during the cell cycle.

As this finding goes against the prevailing notion that protein biosynthesis dynamics are either exponential or constant during the cell cycle^3–7^, we aimed to assess the protein biosynthesis dynamics also with a second, independent method. Therefore, we devised a new technique (the stop-and-respond method) in which we exploited our capability to dynamically monitor the NAD(P)H level in single cells. Specifically, we abruptly halt the activity of a particular enzyme or process, for instance with a chemical inhibitor, in cells asynchronously growing in the microfluidic device, and simultaneously measure each cell’s instantaneous response to this perturbation in terms of the NAD(P)H dynamics. We assume that, if the perturbed enzyme or process was inactive in a cell at the moment of the inhibitor addition, then this would not result in any deviation of the NAD(P)H level from its normal trajectory during the cell cycle. By contrast, if the perturbed enzyme or process was active at the moment of the inhibitor addition, then the enzyme’s substrates would accumulate and the products would be depleted, with these changes further propagating to up- and downstream reactions, some of which likely involve NAD(P)H. Thus, we argue that the magnitude of the perturbation-induced deviation of the NAD(P)H level from its normal cell-cycle related trajectory could serve as a proxy of the enzyme’s activity at the moment of the inhibitor addition.

We applied the stop-and-respond method to determine the protein biosynthesis activity throughout the cell cycle by using cycloheximide (CYH) to halt translation. Specifically, we added CYH to asynchronously growing cells, measured the derivative of NAD(P)H level in individual cells upon the perturbation, and subtracted from it the derivative of NAD(P)H at the same cell-cycle phase before CYH addition (Fig. 1c). The difference between these two derivatives, reflecting the perturbation-associated and normal behavior of NAD(P)H, was considered as NAD(P)H response to CYH in an individual cell at a certain cell-cycle phase. From such single-cell values, by employing Gaussian process regression^20^, we determined the average cell-cycle pattern of NAD(P)H response to CYH. The magnitude of the NAD(P)H response at each cell-cycle phase is assumed to reflect the activity of protein biosynthesis at that phase. Here, we discovered that the cell-cycle pattern of NAD(P)H response to CYH (Fig. 1d) is highly similar to what we found with the sfGFP intensity and cell-volume measurements (Fig. 1a); there are two waves of protein synthesis activity during the cell cycle.

**Extended Data Fig. 2:**
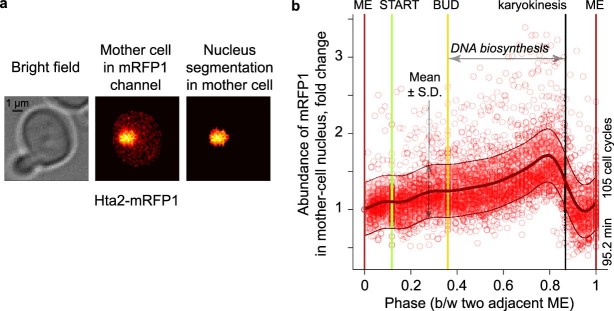
Identification of the typical cell-cycle phase of karyokinesis, which is assumed to denote the end of DNA replication. (**a**) Microscopy image segmentation to locate the nucleus in the mother cell containing mRFP1 fused to the histone protein Hta2, and to calculate the abundance of the fusion. Details of automatic nucleus segmentation are provided in Methods. To calculate the abundance of the fusion Hta2-mRFP in the mother cell nucleus, we sum the intensities of the pixels located within the segmented nucleus. This experiment was performed once, however, similar nucleus segmentation micrographs were observed in all microscopy time points in 105 cell cycles. (**b**) The relative abundance of the fusion Hta2-mRFP1 in the mother-cell nucleus during the cell cycle to identify the phase of karyokinesis. In each individual cell-cycle trace, we divide the abundance by the value at the phase zero (the first mitotic exit ME). The circular markers correspond to different cell-cycle traces and phases. The plot is built analogously to Fig. 1a. Cell cycles whose duration is smaller or equal to 120 mins are considered for this analysis. The average cell-cycle duration measured in minutes is indicated. To summarize the behavior of the relative abundance during the cell cycle, we obtain the Gaussian process (GP) regression, whose mean and region of high posterior probability density (mean ± SD) are presented as the thick curve and the shaded area. The radial basis function (RBF) kernel assuming smoothness with the length scale range [0.05, 0.1] and the white kernel explaining the noise in the data with free noise level are used as a prior. We identified the typical cell cycle phase of karyokinesis as the middle time point between the maximal and minimal values of the GP regression mean after budding (the black vertical line).

The agreement between two independent methods to determine protein production dynamics, first, validated our new stop-and-respond method to infer metabolic activity during the cell cycle. Second, both methods revealed that protein biosynthesis has two activity waves during the cell cycle, one peaking around START and the other in the middle of S/G2/M, opposite to the current notion of protein biosynthesis dynamics^3–7^, but in line with the recent finding of cell-cycle-dependent activity of TORC1 and PKA toward ribosome biogenesis^21^. The protein biosynthesis activity has a minimum around budding as well as 10–20 min before mitotic exit (Fig. 1a,d), which is close to karyokinesis (Extended Data Fig. 2) and may be analogous to the mitotic block of protein biosynthesis in animal cells^22,23^.

**Fig. 2:**
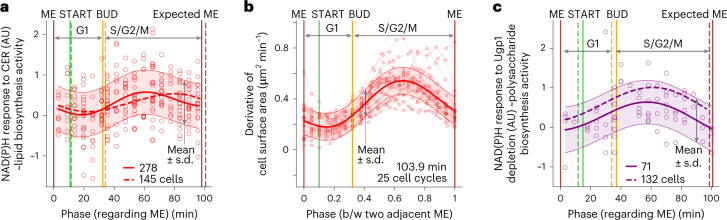
The activities of lipid and polysaccharide biosynthesis change during the cell cycle, peaking in S/G2/M. **a**, The NAD(P)H response to CER varies during the cell cycle, suggesting that lipid biosynthesis activity peaks in S/G2/M. The plot is built analogously to Fig. 1d. The solid curve and shaded area represent the posterior mean and the region of high posterior probability (mean ± s.d.) of a Gaussian process regression summarizing the values of the markers with the help of an RBF kernel. The dashed curve is the posterior mean obtained via the same data analysis pipeline from a replicate experiment, for which we do not show the single-cell values here for the sake of simplicity but indicate the number of analyzed cells. **b**, The derivative of the cell surface area changes during the cell cycle, similarly to the activity of lipid biosynthesis in **a**. The plot is built analogously to Fig. 1a and summarizes 25 cell-cycle traces. The derivative was calculated in smoothed single-cell traces of cell surface area with cytokinesis-associated discontinuity tackled by the *y* axis geometric translation of the data of neighboring cell cycles. **c**, The NAD(P)H response to the auxin-induced Ugp1 depletion changes during the cell cycle, suggesting that cell-wall-polysaccharide biosynthesis activity peaks in S/G2/M. The synthetic auxin 1-naphthaleneacetic acid (NAA) was used to induce the Ugp1 depletion. The plot is built analogously to **a** and Fig. 1d. The NAD(P)H response to NAA in the control strain lacking the degron tag is essentially constant during the cell cycle (Extended Data Fig. 3).

Having found unexpected temporal behavior in protein biosynthesis, we aimed to determine whether dynamics exist also in the biosynthesis of other macromolecular classes. To investigate lipid biosynthesis, we again used the stop-and-respond method; this time with the inhibitor cerulenin (CER) targeting the fatty acid synthase^24^. Here, we found that the dynamic NAD(P)H response to the inhibitor, now reporting lipid biosynthesis activity, is also not constant during cell cycle. In contrast to protein synthesis, we found lipid biosynthesis to be low between START and budding and to peak in the middle of S/G2/M (Fig. 2a). Notably, we identified a similar temporal behavior in the derivative of the cell surface area (Fig. 2b), which, assuming a correlation between the lipid mass in the plasma membrane and the total cellular lipid mass, can be considered a proxy for lipid biosynthesis activity. Together, these data suggest that lipid biosynthesis has the lowest activity in G1, when protein biosynthesis is highly active, but that both biosynthetic processes are active in the middle of S/G2/M (Figs. 1a,d and 2a,b).

**Extended Data Fig. 3:**
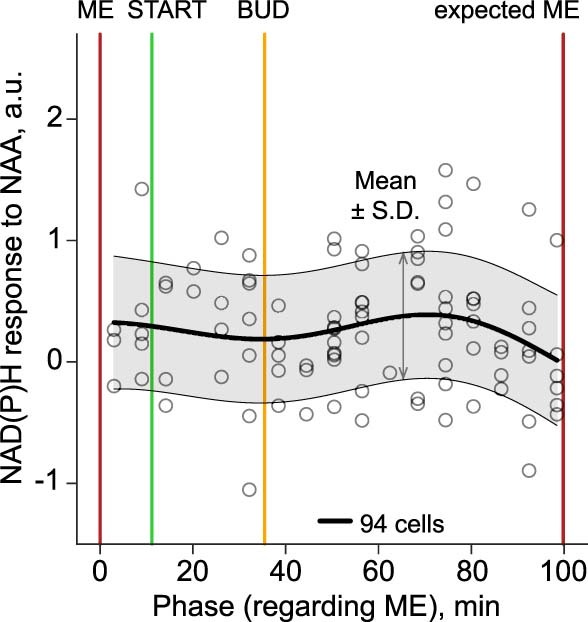
NAD(P)H response to the synthetic auxin 1-naphthaleneacetic acid (NAA) during the cell cycle. The strain treated with NAA lacks the degron tag (the control for the dynamic NAA-induced depletion of Ugp1 having the degron tag; Fig. 2c, the same y axis scaling). The plot is built analogously to Fig. 2c.

Polysaccharides represent another substantial biomass component. Specifically, the cell wall constituents β-glucans, mannan and chitin can account for more than a third of the yeast dry weight^25^, whereas trehalose and glycogen storage can consist of more than 20% of the dry weight under some conditions^26^. To estimate the activity of polysaccharide biosynthesis, we again used the stop-and-respond method; now not with an inhibitor, but with the auxin-inducible degron system^27,28^ to dynamically deplete the enzyme Ugp1 that synthesizes UDP-glucose, the precursor for β-glucans, trehalose and glycogen. Here, we found that the NAD(P)H response to auxin-induced Ugp1 depletion is low in G1 but high in S/G2/M (Fig. 2c), whereas the response to auxin in a control strain does not show these dynamics (Extended Data Fig. 3). Because there is only minor production of trehalose and glycogen under the high-glucose conditions investigated here^29–31^, the recorded NAD(P)H response to Ugp1 depletion must primarily reflect the activity of the synthesis of β-glucans, which are the major component of the cell wall^32^. Indeed, the response to Ugp1 depletion is similar to the above reported derivative of the cell surface area (Fig. 2b), which can be considered a proxy for the rate of cell-wall construction. Thus, akin to lipids, cell wall polysaccharides are predominantly synthesized in S/G2/M when the bud emerges and grows.

Through Bayesian and frequentist model selection criteria^20^, we confirmed that the oscillatory functions, namely two waves of protein biosynthesis activity and one wave of lipid and polysaccharide biosynthesis activity during the cell cycle, explain the experimental data better and have higher predictive performance as compared to linear, including constant-linear functions (Supplementary Table 5).

Together, these data demonstrate that the biosynthesis of proteins, lipids and polysaccharides is temporally segregated during the cell cycle. Most notably, while protein biosynthesis activity peaks twice, the activities of lipid and polysaccharide biosynthesis peak only once in S/G2/M.

### Biosynthetic rates are inferred with model-based analysis

Cell growth during the cell cycle is often viewed only in terms of integral variables such as cell size or cell mass; however, our finding of a temporal segregation among the different biosynthetic activities suggests that cell growth should be considered in a more differentiated manner involving individual biosynthetic processes. To this end, we next set out to quantify the contribution of each major biosynthetic process to the overall rate of cell-mass increase at each phase throughout the cell cycle.

**Fig. 3:**
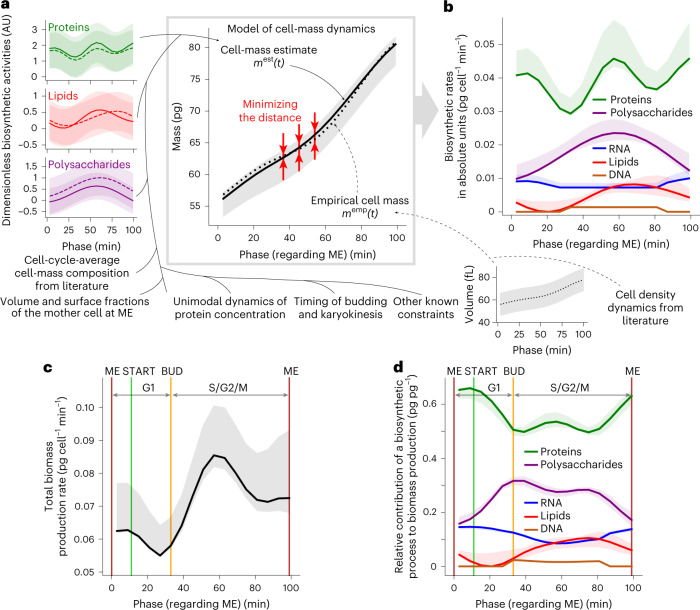
Cell-cycle dynamics of biosynthetic rates inferred with model-based analysis. **a**, The mathematical model describes the dynamics of the cell-mass development along the cell cycle. The model (1) combines single-cell measurements, such as the activities of protein, lipid and polysaccharide biosynthesis, cell volume, fractions of mother-cell volume and surface with regard to the whole cell, timing of cell-cycle events; (2) incorporates literature-derived knowledge of cell-density dynamics and cell-cycle-average cell-mass composition; and (3) infers the biosynthetic rates of five major biomass components expressed in absolute units (pg min^−1^). To implement this inference, we minimize the distance between the cell-mass estimate, which is a function of the discovered biosynthetic patterns (Figs. 1d and 2a,c) and the empirical cell mass obtained by multiplying our dynamic cell-volume measurements (Extended Data Fig. 4) and cell-density measurements^33^ at corresponding cell-cycle phases. For proteins, lipids and polysaccharides, we show mean ± s.d. of biosynthetic activities measured in two replicate experiments (left). Data are from one experiment and shown as mean ± s.d. (right: volume). Model equations are provided in Supplementary Methods. **b**, The inferred biosynthetic rates of five major biomass components expressed in absolute units (pg min^−1^). **c**, Inferred total biomass production rate *r*_biomass_(*t*) during the cell cycle, computed by summing up the rates of protein, RNA, lipid, polysaccharide and DNA biosynthesis in **b** at each phase of the cell cycle. **d**, Inferred relative contributions of biosynthetic process to the total biomass production throughout the cell cycle. To calculate the relative contributions, we divided individual biosynthetic rates in **b** by the total biomass synthesis rate *r*_biomass_(*t*) in **c** at each phase of the cell cycle. For data presentation for cell-mass estimate in **a** and all variables in **b**–**d**, an error band shows the minimum–maximum range of an inferred variable among eight model optimizations covering all combinations of replicate measurements of protein, lipid and polysaccharide biosynthesis (**a**, left) as inputs; a thick line shows an inferred variable in the model optimization that uses the input dataset where two replicate measurements of each macromolecule biosynthesis were averaged.

**Extended Data Fig. 4:**
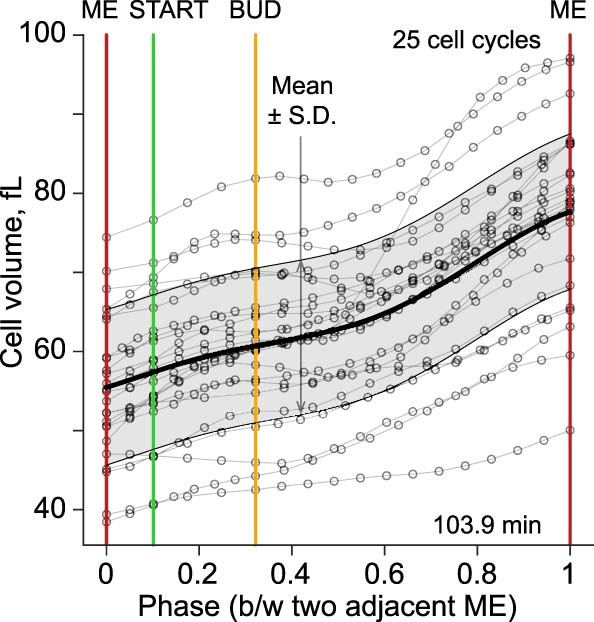
Cell volume during the cell cycle. Cell volume dynamics is presented as a summary of 25 individual cell-cycle traces. The plot is built analogously to Figs. 1a, 2b.

Here, the challenge was to translate our determined dimensionless biosynthetic activities into rates expressed in absolute units (pg min^−1^) and to infer the cell-cycle-dependent rates in the synthesis of the remaining major biomass components, namely DNA and RNA. For this, we formulated an algebraic model (Fig. 3a) that describes the development of total cell mass over the cell cycle as a function of the pg min^−1^-expressed biosynthetic rates. The cell-mass development over the cell cycle (Fig. 3a; ‘cell-mass estimate’) was defined by these temporally changing biosynthetic rates, which were determined via the dimensionless biosynthesis patterns (Fig. 3a, left) multiplied by conversion factors to obtain absolute units and via other constraints (see below). By fitting this model to cell-cycle-resolved cell-mass data (Fig. 3a, ‘empirical cell mass’), obtained from our dynamic cell-volume measurements (Extended Data Fig. 4) and cell-cycle-dependent cell-density values^33^, we could infer the absolute cell-cycle-resolved biosynthetic rates (Fig. 3b).

Specifically, our model describing the dynamics of cell mass during the cell cycle has the following features and assumptions (Supplementary Methods): (1) DNA synthesis was assumed to occur at a constant rate between budding^18,19^ and karyokinesis. Timing of budding and karyokinesis was obtained from microscopic experiments; budding is clearly visible under bright-field illumination and karyokinesis was identified as the rapid decrease of tagged histone protein Hta2–mRFP1 in the mother cell (Extended Data Fig. 2). (2) RNA synthesis rate was considered as the sum of rRNA, the most abundant RNA type, and non-rRNA-synthesis rates. Non-rRNA was assumed to be produced at a constant rate, as was rRNA between budding and karyokinesis. rRNA synthesis rate was considered proportional to the protein translation rate from ~15 min before mitotic exit through to budding. This assumption was based on transcriptomics data showing that rRNA processing and ribosome biogenesis gene expression peaks once during the cell cycle in G1 (refs. ^11,34^). (3) The rates of protein, lipid and polysaccharide biosynthesis in pg min^−1^ were estimated by multiplying their respective dimensionless activities (Fig. 3a, left) with conversion factors determined in the fitting. The dimensionless activities were allowed to move vertically (to undergo geometric translation) within their uncertainty bounds. (4) The mass of each biomass component at every given time point was calculated as the sum of the component’s initial mass and the integral of its biosynthetic rate over the time duration from the latest cell division. The initial mass of protein and RNA was defined by their masses at cell division multiplied by the measured volume fraction of the mother-cell compartment relative to the whole cell at that time point. For the lipid and polysaccharide initial masses, the cell-surface-area fraction of the mother compartment was used instead. (5) Finally, the dynamic cell-mass estimate was calculated using the masses of all five macromolecule classes and the water in their hydration shells, whose size we constrained according to literature^35–39^, as well as using the mass of free water and metabolites scaled with the measured cell volume. Previously reported values on cell-cycle-average mass fractions of major biomass components at high growth rates^40^ and water^41,42^ were used to constrain the model.

**Extended Data Fig. 5:**
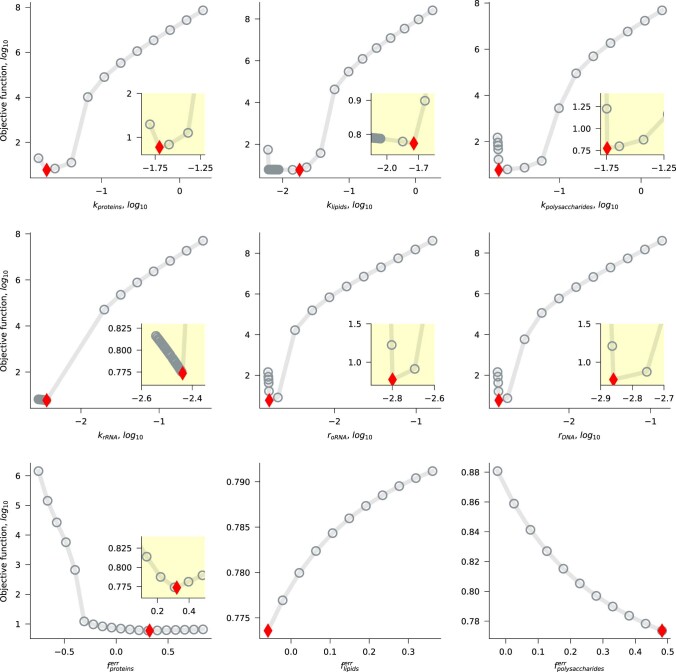
The cell-mass model parameters used to define the biosynthetic rates are structurally identifiable according to profile likelihood analysis. We considered 9 parameters defining the rates of protein, lipid, polysaccharide, RNA and DNA biosynthesis in the model of cell-mass dynamics during the cell cycle (details in Supplementary Methods). For each fixed parameter value (x-values of empty circular markers), we optimized the model over the rest of parameters and reported the objective function value (y-values of empty circular markers). The red diamond marker shows the result of the free optimization in which the parameters of interest were not fixed. The yellow internal plot zooms in on the proximity of the red marker. For each of the 9 parameters, the objective function in the optimization where the parameter is free proves to be the smallest compared to the optimizations where the parameter is fixed to a value different from the optimal one, which demonstrates that the 9 parameters of interest are structurally (globally) identifiable according to profile likelihood approach [main-text ref. ^43^]. The optimal value of $f_{lipids}^{\,err}$ is at the lower boundary of feasibility; this parameter cannot be smaller, otherwise the rate of lipid biosynthesis becomes negative at some cell-cycle phases. The optimal value of $f_{polysaccharides}^{\,err}$ is at the upper boundary of feasibility defined by the variability of the measurement of the polysaccharide biosynthesis activity. The profile likelihood analysis was performed using the input data set where the replicate measurements of each biosynthetic activity were averaged (the respective simulation results are showed via the solid line in Fig. 3b–d).

We then used mathematical optimization to minimize the difference between the empirical cell mass dynamics, as determined by the cell volume and density and the model-based dynamic cell-mass estimate. With this optimization, we inferred the biosynthetic rates for each of the five major biomass components, namely proteins, lipids, polysaccharides, DNA and RNA in absolute terms, expressed in pg min^−1^ (Fig. 3b). A profile likelihood analysis^43^ confirmed structural identifiability of the model parameters defining these rates (Extended Data Fig. 5).

The obtained rates allowed us to quantitatively compare the different biosynthetic processes among each other. For instance, protein biosynthesis was found to have the highest mass-increase rate values of all biosynthetic processes with its lowest value still being higher than the maximum of polysaccharide biosynthesis (Fig. 3b). Summing up the inferred biosynthetic rates, we found that the total biomass production rate has two peaks during the cell cycle (Fig. 3c). Dividing the individual biosynthetic rates by the total biomass production rate, we obtained the cell-cycle-phase-dependent relative contribution of each biosynthetic process to the total biomass production (Fig. 3d). The relative contribution of protein biosynthesis to the total biomass production was found to be higher around mitotic exit and throughout G1 compared to the biggest part of S/G2/M (Fig. 3d), when most of the biosynthetic processes peak (Fig. 3b). Thus, our model-based analysis revealed the relative contribution of the individual biosynthetic processes to cell growth during the cell cycle, which is apparently much more variable and dynamic than previously thought.

### Altering biosynthetic rates change primary metabolic fluxes

Next, we hypothesized that the uncovered temporal segregation of the biosynthetic processes could be the reason why metabolism shows dynamics during the cell cycle, which were observed in single cells in the form of hour-scale-oscillating cofactor levels and referred to as metabolic oscillations^44^. Our approach to test this hypothesis was the following: we used a recently developed thermodynamic-stoichiometric model of yeast metabolism^45^ to infer the flux dynamics in primary metabolism that are necessary to satisfy the cell-cycle-dependent requirements of the biosynthetic pathways. If the respective model-inferred metabolic flux dynamics could be supported by independent experimental observations, then this would suggest that the metabolic dynamics, as observed in primary metabolism of yeast, are indeed in place to meet the identified cell-cycle-dependent biosynthetic rates.

To infer the metabolic flux dynamics during the cell cycle as required to meet the temporally changing biosynthetic dynamics, we first had to adjust the earlier developed thermodynamic-stoichiometric metabolic model. Specifically, we had to split the model’s biomass equation into five separate equations, each respectively defining the production of proteins, lipids, cell-wall polysaccharides, RNA and DNA, and to introduce a new biomass equation that combines these five major biomass components into the final biomass as the end product. After a regression analysis to determine the model’s parameters (standard Gibbs energies of reactions) as conducted previously^45^, we had a stoichiometric-thermodynamic metabolic network model with which we could perform flux balance analysis (FBA)-type predictions for each moment in the cell cycle.

**Fig. 4:**
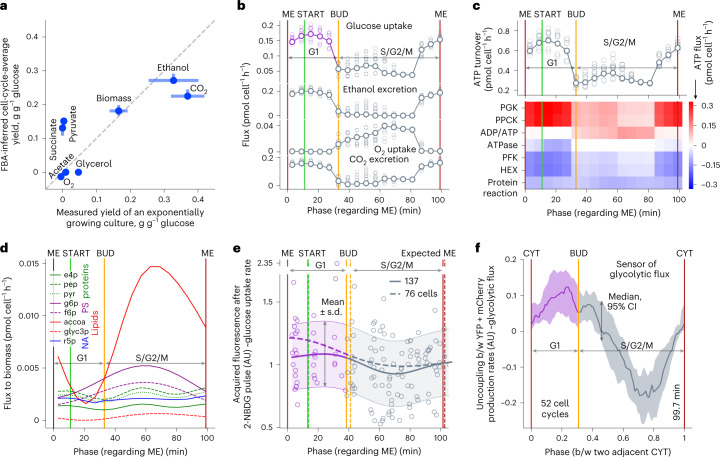
Dynamic phenotypes of primary metabolism predicted via the biosynthetic rates agree with experimental observations. **a**, Yields of extracellularly exchanged metabolites with respect to glucose agree between cell-cycle-averaged flux predictions and independent measurements in an exponentially growing culture (*x* axis, mean ± s.d. from elsewhere^89^; *y* axis data described below). **b**, Cell-cycle dynamics of predicted fluxes in the primary carbon and energy metabolism. **c**, Predicted turnover of cytoplasmic ATP during the cell cycle and the ATP fluxes in reactions that are largest producers or consumers of this metabolite. The turnover was calculated as the sum of ATP fluxes in reactions where this metabolite is produced. We show reactions whose cytoplasmic ATP flux is bigger than 0.09 or smaller than −0.09 mol cell^−1^ h^−1^ in at least one cell-cycle phase. PGK, phosphoglycerate kinase; PPCK, phosphoenolpyruvate carboxylase kinase; ADP/ATP, adenine nucleotide translocator (oxidative phosphorylation); PFK, phosphofructokinase; HEX, hexokinase. **d**, Predicted fluxes of biomass precursors diverting from central carbon and energy metabolism to the synthesis of major biomass components. NA, nucleic acids; PS, polysaccharides; e4p, erythrose 4-phosphate; pep, phosphoenolpyruvate; pyr, pyruvate; g6p, glucose 6-phosphate; f6p, fructose 6-phosphate; accoa, acetyl-CoA; glyc3p, glycerol 3-phosphate; r5p, ribose 5-phosphate. Vertical lines denote typical cell-cycle phases of major cell-cycle events (**b**–**d**). For presentation of data (*y* axis) in **a**–**d**: predictions shown by markers in **a**, line-connected bigger markers in **b** and **c**, heat map in **c** and lines in **d** correspond to the output of the cell-mass model provided with averaged replicate measurements of macromolecule biosynthesis (solid lines in Fig. 3b–d); predictions shown by *y* axis error bars (min–max range) in **a** and smaller markers in **b** and **c** correspond to the output of the cell-mass model using eight different combinations of replicate measurements (shaded area in Fig. 3b–d). **e**, Acquired intracellular fluorescence after a pulse of the glucose analog 2-NBDG varies during the cell cycle. Solid curve and shaded area indicate posterior mean and region of high posterior probability (mean ± s.d.) of the Gaussian process regression summarizing single-cell values (markers) via an RBF kernel. Dashed curve: posterior mean obtained via the same data analysis pipeline in the replicate experiment (number of analyzed cells indicated). **f**, Production rates of YFP and mCherry, having and lacking glycolytic flux regulation, respectively are uncoupled during the cell cycle in cells expressing the glycolytic flux biosensor. The uncoupling was calculated in individual cell‐cycle traces as the difference between the momentary production rates of YFP and mCherry normalized to have the same scale. A higher value of the uncoupling reflects a higher production rate of YFP with respect to the production rate of mCherry and thus a higher value of the glycolytic flux. Curve and shaded area show median and its 95% CIs. To align individual cell‐cycle traces and calculate phases, we used as reference points cytokinesis (CYT, 0), budding and next cytokinesis (CYT, 1).

For these simulations, we used the momentary relative contributions of the biosynthetic rates to the total biomass production (Fig. 3d), which we obtained by relating the individual biosynthetic rates (Fig. 3b) to the total biomass production rate (Fig. 3c). We used these momentary relative contributions to define the stoichiometric coefficients of the respective biomass components in the model’s biomass equation in a cell-cycle-dependent manner. For different discrete moments during the cell cycle, we assumed a quasi-steady state and ran FBA simulations, where we maximized the flux through the respectively defined biomass equation, while the model was constrained by the earlier identified upper limit in the cellular Gibbs energy dissipation rate^45^. As a global validation of the simulation results, we used the predicted cell-cycle-resolved physiological parameters, then computed from them the population-level (cell-cycle average) yield coefficients and compared these to experimentally measured ones. Supporting the validity of the simulations, we found that the computed values showed good agreement with those measured in a batch culture grown on high glucose, in particular reflecting the globally fermentative mode of metabolism (Fig. 4a).

**Extended Data Fig. 6:**
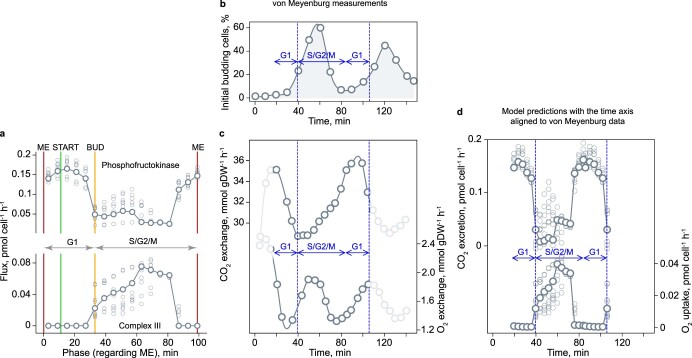
Additional model predictions and comparison with independent measurements. (**a**) Primary metabolism is predicted to alternate between fermentative and respiratory modes during the cell cycle. The predicted flux dynamics in glycolysis (phosphofructokinase) and in electron transport chain (Complex III). See the caption of Fig. 4b for more details. (**b–d**) Predicted carbon dioxide and oxygen exchange rates match von Meyenburg measurements performed in synchronized batch cultures grown on high glucose [main-text ref. ^46^]. (b,c) von Meyenburg measurements: cells from the late stage of budding phase were isolated via dextrin gradient centrifugation and cultivated in a high-glucose batch culture, with the synchrony controlled by monitoring the fraction of initial budding cells. Here, the beginning of the S phase is defined as the time point corresponding to one third of the ascending part in each wave of the initial budding cell percentage (b). The G1 boundaries are established by considering that G1 constitutes 33% of the total cell-cycle duration on high glucose as observed in this work. (d) The predicted fluxes of carbon dioxide excretion and oxygen uptake. These data are also shown in Fig. 4b, more details can be found in its caption.

Focusing on the inferred cell-cycle-resolved fluxes, we found that the glucose-uptake flux (Fig. 4b) and glycolytic flux (Extended Data Fig. 6a) markedly change during the cell cycle; these fluxes are high in G1, drop after budding and stay low for the largest part of S/G2/M, before they rise again toward mitotic exit. High ethanol excretion fluxes occur during the phases of high glucose uptake (Fig. 4b). Oxygen uptake flux (Fig. 4b) as well as the flux through the electron transport chain (Extended Data Fig. 6a) are high after budding during the biggest part of S/G2/M. Carbon dioxide is excreted mostly around mitotic exit and in G1 (Fig. 4b). The turnover rate of cytoplasmic ATP shows highest values around mitotic exit and in G1, with the most important ATP-producing reactions being phosphoglycerate kinase and phosphoenolpyruvate carboxylase kinase (Fig. 4c; PGK and PPCK).

We could also estimate the rates at which precursor metabolites are employed to satisfy the momentary biosynthetic requirements. The fluxes running from erythrose 4-phosphate, phosphoenolpyruvate and pyruvate to biomass follow two waves per cell cycle to satisfy protein synthesis (Fig. 4d). In contrast, while acetyl-CoA is needed for both protein and lipid biosynthesis, the flux running from acetyl-CoA to biomass has only one wave per cell cycle (Fig. 4d), reflecting a larger acetyl-CoA demand for the once-oscillating lipid biosynthesis (Fig. 2a).

For our model simulations, we used a number of assumptions and it is thus important to further validate the model predictions. Specifically, we assumed that (1) fluxes are geared to biomass optimality; that (2) fluxes are at quasi-steady state; that (3) there is an upper limit in the cellular Gibbs energy dissipation rate as recently identified^45^; and that (4) the above-determined temporally segregated dynamics of the biosynthetic processes are correct (Fig. 3). If these assumptions are correct, then the cell should exhibit high rates of glucose uptake toward mitotic exit and in G1 and low rates in the middle of S/G2/M, as shown in Fig. 4b. Furthermore, primary metabolism should respectively alternate between a fermentative and respiratory metabolism during the cell cycle (Fig. 4b and Extended Data Fig. 6a). In case these predictions agreed with independent experimental data, then this would suggest that the temporal segregation in the biosynthetic processes is indeed responsible for the metabolic dynamics in primary carbon and energy metabolism.

In fact, data from synchronized high-glucose batch cultures^46^ match with our predictions; in line with our predicted oxygen and CO_2_ exchange rates, O_2_ uptake and CO_2_ excretion rates were found to oscillate almost in antiphase to each other (Extended Data Fig. 6b–d), with the O_2_ uptake peaking soon after the initiation of budding and the CO_2_ excretion peaking in the late S/G2/M and G1 (ref. ^46^). Furthermore, the model predicted markedly changing glucose-uptake fluxes, namely high fluxes in G1 and several minutes before mitotic exit and low fluxes during the biggest part of S/G2/M (Fig. 4b). We aimed to validate these predictions with cell-cycle-resolved single-cell measurements of the glucose-uptake flux. First, we administered a ~13–15-min pulse of 2-NBDG, a fluorescent non-metabolizable glucose analog, to cells growing asynchronously in the microfluidic chamber on glucose and used the acquired intracellular fluorescence to assess the glucose-uptake flux at different cell-cycle stages. Here, we found that the intracellular fluorescence acquired following the 2-NBDG pulse varies depending on the cell-cycle phase. Particularly, the fluorescence increase, and thus glucose-uptake flux, is higher in G1 than during S/G2/M (Fig. 4e), which agrees with our model predictions (Fig. 4b).

**Extended Data Fig. 7:**
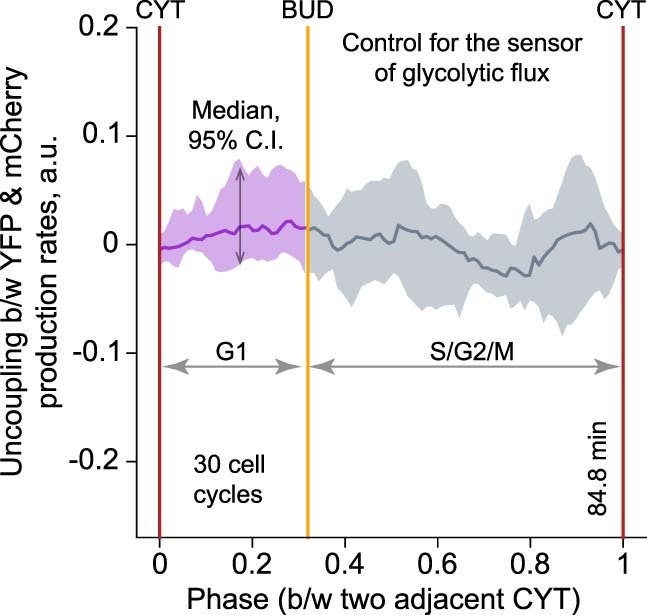
The uncoupling between the YFP and mCherry production rates in the control strain lacking the glycolytic flux regulation of YFP expression (the control experiment for Fig. 4f). The figure was obtained after the same analysis as in Fig. 4f, see more details in its caption and Methods.

Second, to further test these predictions, we employed a glycolytic flux biosensor that expresses yellow fluorescent protein (YFP) under the control of a glycolytic flux-sensing transcription factor and mCherry from a constitutive promoter^47^. By continuously recording YFP and mCherry fluorescence as well as cell volume, we could determine the momentary production rates of YFP and mCherry in single cells. The difference between these two production rates (their uncoupling) during the cell cycle is a proxy for the momentary glycolytic flux. Here, again consistent with the model predictions, we found that the uncoupling between the YFP and mCherry production rates changes throughout the cell cycle, with the higher uncoupling toward mitotic exit and in G1, suggesting high glycolytic flux in this phase (Fig. 4f). In a control strain, the uncoupling is constant throughout the cell cycle (Extended Data Fig. 7).

**Extended Data Fig. 8:**
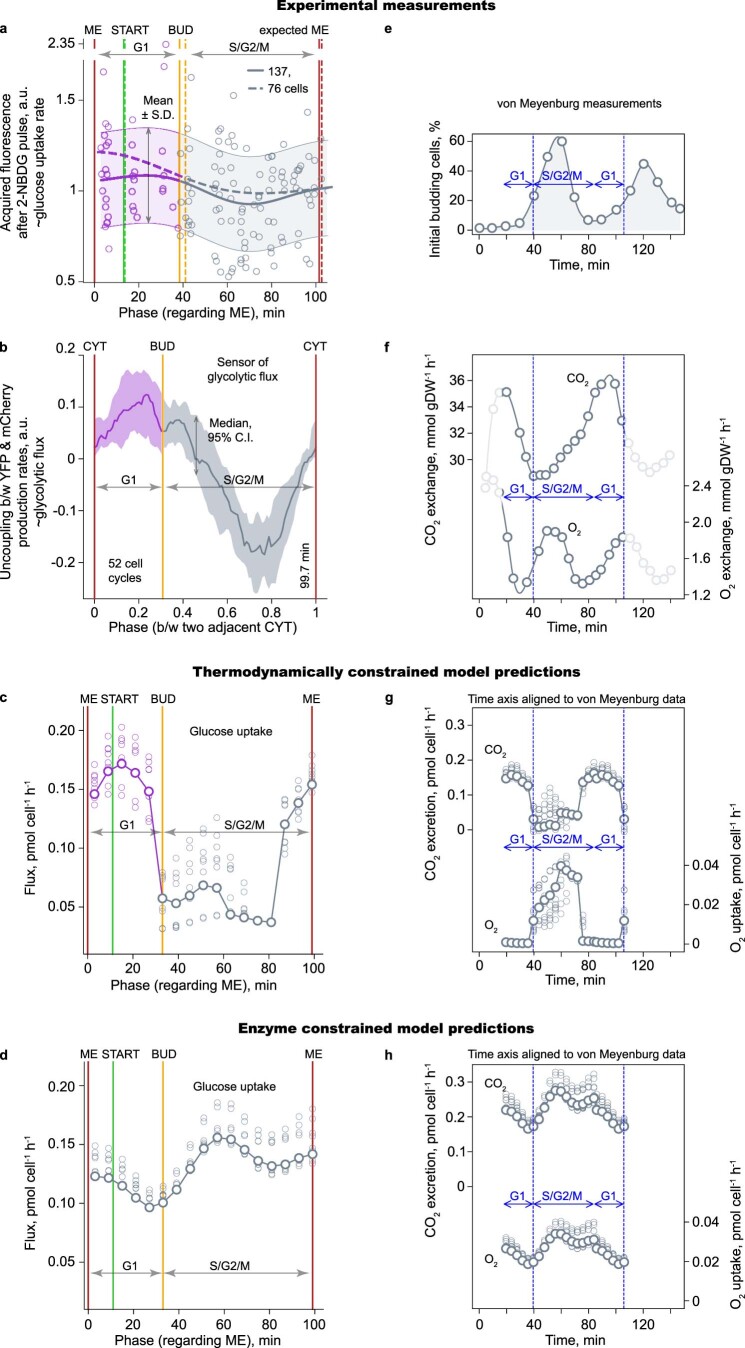
Thermodynamically constrained model better predicts the cell-cycle dynamics of key metabolic fluxes compared to enzyme-constrained model. (**a**) Glucose-uptake flux measurements, described in Fig. 4e. (**b**) Glycolytic flux measurements, described in Fig. 4f. (**c**) Glucose-uptake flux predictions of thermodynamically constrained model, described in Fig. 4b. In agreement with independent experimental measurements (a, b), the model predicts the highest fluxes (violet) in G1 and the trough of the flux dynamics in S/G2/M. (**d**) Glucose-uptake flux predictions of enzyme-constrained model. This model predicts the highest glucose-uptake-flux values in the middle of S/G2/M, contradicting experimental observations (a, b). (**e,f**) Measurements of carbon dioxide and oxygen exchange rates, described in Extended Data Fig. 6c. (**g**) O_2_-uptake and CO_2_-excretion flux predictions of thermodynamically constrained model, described in Fig. 4b. In agreement with experimental measurements (f), the model predicts that O_2_-uptake and CO_2_-excretion rates oscillate almost in antiphase, O_2_ uptake peaks soon after budding and CO_2_ excretion peaks in late S/G2/M and G1. (**h**) O_2_-uptake and CO_2_-excretion flux predictions of enzyme-constrained model. This model predicts the highest values of CO_2_-excretion flux in the middle of S/G2/M and synchronized dynamics of O_2_-uptake and CO_2_-excretion fluxes, contradicting independent observations (f). d,h: we predicted metabolic fluxes via the model with constraints over enzyme kinetics and abundance (GECKO, v.2.0.2) [main-text ref. ^48^] by incorporating the cell-cycle-resolved macromolecular synthesis rates (Fig. 3c, d) identically to how we did it with thermodynamically constrained model. We used the same stoichiometry of macromolecule and biomass reactions (Supplementary Table 7). In GECKO, the total amount of enzyme had an upper limit such that the model could freely distribute individual enzyme amounts under this overall constraint. In model simulations for all cell-cycle time points, we set the upper limit of the total enzyme amount to 0.0445 g/gDW, under which GECKO with modified macromolecule reaction stoichiometries correctly predicts, at the population level, changes of yeast physiology (the rates of growth, oxygen uptake, ethanol and carbon dioxide excretion) across the range of glucose uptake rates [main-text ref. ^40^]. GECKO was accessed with the help of the Python module geckopy.

Thus, the key metabolic feature predictions, obtained when using the identified temporally segregated biosynthetic rates (Fig. 3b–d) as input of the thermodynamically constrained model, are in agreement with independent experimental observations. These include population-level physiological parameters in a batch culture (Fig. 4a), gas-exchange dynamics previously determined in synchronized high-glucose batch cultures (Extended Data Fig. 6b–d)^46^ and cell-cycle-resolved metabolic activity dynamics, such as glucose-uptake flux (Fig. 4e) and glycolytic flux (Fig. 4f) measured in single cells. Notably, an enzyme-constrained model^48^ generated flux predictions that could not be validated by these independent experimental observations (Extended Data Fig. 8), which suggests that the limit on the cellular Gibbs energy dissipation rate is key to predict correct cell-cycle-resolved fluxes. The agreement between the thermodynamically constrained model predictions and the independent measurements suggests that the temporally segregated biosynthetic processes are responsible for the metabolic oscillations, reflecting a rewiring of the fluxes in the primary metabolism to meet the changing demands in building blocks and energy.

### NAD(P)H dynamics support biosynthetic temporal segregation

Our conclusion that the temporal segregation of biosynthetic processes dictates the primary metabolic dynamics has a number of direct consequences. First, the earlier conjectures on the causes of metabolic dynamics during the cell cycle, such as respiratory activity^34,49,50^ and carbohydrate-storage turnover^51–53^, should not be correct. Second, as the temporal segregation of biosynthetic processes is likely a condition-independent behavior, metabolic dynamics should occur across all nutrients on which cells grow and divide. Third, inhibition of biosynthetic processes should halt the metabolic dynamics. If we show that these envisioned consequences of our finding are correct, then this would serve as additional validation for what we put forward.

To test the proposed consequences, we made use of our ability to dynamically measure NAD(P)H levels in individual cells. We expect that flux changes in primary metabolic pathways would lead to transient imbalances between metabolites’ production and depletion and thereby to temporal changes in the metabolite levels. The effect of such imbalances should be seen in single cells in terms of dynamically changing NAD(P)H levels.

**Fig. 5:**
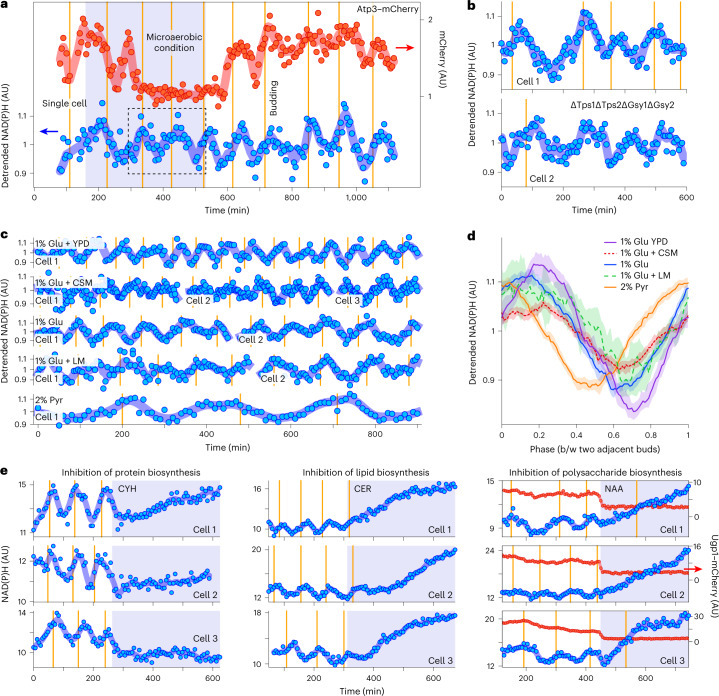
Evidence of the temporal segregation of biosynthetic processes is found in NAD(P)H dynamics. **a**, NAD(P)H oscillations are unperturbed (dashed rectangle) in microaerobic condition with disrupted fluorescence dynamics of mCherry fused to ATP synthase subunit Atp3. Methods describe technical solutions to attain the microaerobic condition. More cells are shown in Extended Data Fig. 9. **b**, NAD(P)H oscillations exist in a strain lacking two subunits of trehalose-6-phosphate synthase/phosphatase complex *TPS1* and *TPS2*, and two paralogs encoding glycogen synthase *GSY1* and *GSY2*. **c**,**d**, NAD(P)H oscillations are present in cells growing in various media: minimal medium containing 1% glucose (Glu), 2% pyruvate (Pyr), combination of 1% Glu with a lipid mixture (LM; seven fatty acids) or with a complete supplement mixture (CSM; 12 amino acids, two nucleobases); complex medium YPD with 1% Glu. NAD(P)H oscillations with respect to absolute time in single cells growing in indicated medium and going through several cell and metabolic cycles (**c**). Summarized NAD(P)H oscillations with respect to cell-cycle-relative time (phase) in multiple cells growing in indicated medium (**d**). Curves and shaded areas show median and its 95% CI. Numbers of individual cell cycles (and single cells going through them) in each condition are 355 (102) for 1% Glu; 268 (93) for 2% Pyr; 27 (9) for 1% Glu + LM; 258 (98) for 1% Glu + CSM; and 124 (15) for 1% Glu YPD. NAD(P)H fluorescence values were detrended and normalized by performing LOWESS in an entire single-cell trace (large window size for line fits) and dividing raw NAD(P)H values by the resulting LOWESS curve (**a**–**d**). Markers show detrended values; curves show LOWESS (small window size for line fits) smoothing of detrended values (**a**–**c**). Window sizes used in LOWESS for detrending and smoothing are shown in Supplementary Table 8. Phase shifts and cell-cycle coupling of NAD(P)H oscillations across growth conditions are shown in Extended Data Fig. 10. **e**, NAD(P)H oscillations cease when CYH and CER are added and when Ugp1 is depleted via NAA-induced degradation. Markers show raw (not detrended and normalized) NAD(P)H or Ugp1-mCherry fluorescence; curves show smoothing with LOWESS (six and three data points for line fitting). Vertical lines indicate budding (**a**–**e**).

**Extended Data Fig. 9:**
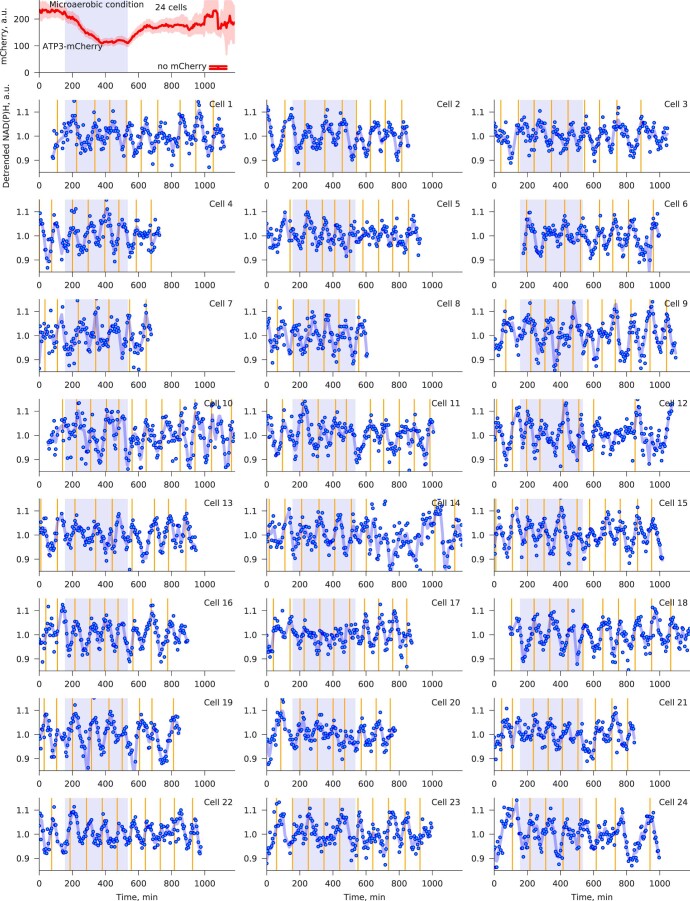
NAD(P)H oscillations are unperturbed in the microaerobic condition indicated by a disruption in the dynamics of the fluorescence of mCherry fused to the ATP synthase subunit Atp3. The upper plot summarizes the dynamics of mCherry fluorescence in 24 cells with the mean and its 95% confidence interval. The NAD(P)H dynamics of these 24 cells are shown separately below. Cell 1 is also shown in Fig. 5a. With the whiskers in the upper plot, we show the spread of values of a cell not expressing mCherry that was monitored in the mCherry microscopy channel in a separate experiment. See more details in the caption of Fig. 5 and Methods.

By measuring NAD(P)H levels in single cells as a readout of biosynthetic and primary metabolic dynamics, we tested whether the above-mentioned consequences of our finding are correct. First, the conjectures that metabolic oscillations are caused by dynamics in respiration^34,49,50^ or carbohydrate-storage metabolism^51–53^ are expected to be incorrect. Indeed, decreasing the oxygen content in the microfluidic device, confirmed by a drop in the level of mCherry-tagged γ-subunit Atp3 of the ATP synthase^54^, did not affect the NAD(P)H oscillations (Fig. 5a and Extended Data Fig. 9). This suggests that mitochondrial respiration can be excluded as a cause of the metabolic oscillations, in line with recent observations^55^. Furthermore, after deleting the four genes needed for trehalose and glycogen biosynthesis, *TPS1*, *TPS2*, *GSY1* and *GSY2*, and thus removing any possibility for carbohydrate-storage production^30^, we still observed NAD(P)H oscillations (Fig. 5b), which demonstrates that also dynamics in carbohydrate-storage metabolism are not the cause of the metabolic oscillations.

**Extended Data Fig. 10:**
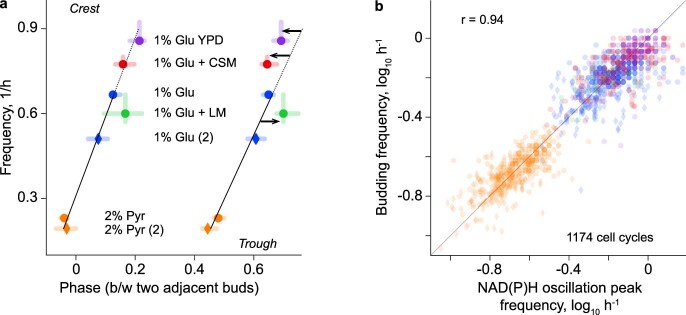
Phase shifts and cell-cycle coupling of NAD(P)H oscillations across growth conditions. (**a**) NAD(P)H oscillations are phase-shifted across growth conditions. NAD(P)H oscillation frequency versus the crest and trough phases in different conditions: minimal medium containing 1% glucose (Glu), 2% pyruvate (Pyr), combination of 1% Glu with a lipid mixture (LM, 7 fatty acids) or with a complete supplement mixture (CSM: 12 amino acids, 2 nucleobases); complex medium YPD with 1% Glu. Markers and error bars: median and its 95% confidence intervals. Circular markers: data sets of Fig. 5d, diamonds: replicates in 1% Glu and 2% Pyr with 155 (89) and 192 (80) cell cycles (and cells). Inclined lines: relationship between frequency and crest/trough phase in single carbon and energy source conditions (linear regression of four 1% Glu and 2% Pyr median values). Arrows: pronounced deviations from these lines (no overlap between a line and both confidence intervals). Pronounced deviations from the trough line have different directions between the cases of YPD and CSM (protein and nucleic-acid precursors) versus the case of LM (lipid precursors), suggesting temporal segregation between respective biosynthetic processes. (**b**) NAD(P)H oscillations and cell cycle are strongly coupled across growth conditions. Each marker corresponds to a pair of adjacent budding events, for which we calculated budding frequency and NAD(P)H oscillation peak frequency. We used the same data set as in (a) and indicated different growth conditions with the same colors and marker styles. The following numbers of budding pairs (cell cycles) were used: 319 for 1% Glu, 125 for 1% Glu (2), 234 for 2% Pyr, 161 for 2% Pyr, 26 for 1% Glu + LM, 192 for 1% Glu + CSM and 117 for 1% Glu YPD. We present the Pearson correlation coefficient for the frequencies.

Second, with the temporal segregation of biosynthesis likely being a condition-independent behavior, metabolic dynamics should occur under all growth conditions. To test this, we performed a series of microfluidic experiments, in which we provided cells with different nutrients that are utilized through different metabolic pathways, and monitored the NAD(P)H dynamics in single cells. Growth medium included a minimal medium with either glucose or pyruvate, a glucose minimal medium supplemented with either fatty acids or amino acids and nucleobases, and a complex medium with glucose (YPD). In all these conditions, across which the median cell-cycle duration varied between 70 and 260 min and which included largely different metabolic operations such as fermentation and respiration, we found NAD(P)H oscillations (Fig. 5c,d) in line with our finding that the uncovered temporal segregation in biosynthesis, rather than a specific primary metabolic pathway, is responsible for the metabolic oscillations. In fact, phase shifts, which we observed between the NAD(P)H oscillations when different biomass precursors (namely, fatty acids, amino acids and nucleobases, components of YPD) were provided in the growth medium, are consistent with the finding of the temporal segregation between different biosynthetic processes (Extended Data Fig. 10a).

Finally, if our finding of the temporal segregation in biosynthesis is correct, then inhibition of biosynthetic processes should halt the metabolic dynamics. To test this, we returned to the experiments in which we inhibited protein biosynthesis with cycloheximide, lipid biosynthesis with cerulenin and polysaccharide biosynthesis with the auxin-inducible depletion of Ugp1 in cells growing in the microfluidic device. Here, in these dynamic inhibition experiments, we observed that NAD(P)H levels stopped oscillating immediately after the addition of the inhibitors or auxin (Fig. 5e). Thus, by confirming all three envisioned consequences, we provided additional support for the uncovered temporal segregation of biosynthetic processes and for this segregation to be the cause of the flux dynamics in primary metabolism.

## Discussion

Using dynamic perturbation experiments and new microscopic single-cell analyses, we uncovered how the activities of biosynthetic processes are organized in time during the cell cycle of budding yeast. We found that the protein biosynthesis activity has two waves per cell cycle, one in G1 and the other in S/G2/M, whereas the activities of lipid and polysaccharide biosynthesis synchronously peak only once in S/G2/M. Through integration of the generated dynamic biosynthesis data in mathematical models, we determined changes of metabolic fluxes through primary metabolism that are required to meet the temporally changing biosynthetic activities. We could experimentally validate the inferred metabolic fluxes and found additional evidence for the temporal segregation of biosynthetic processes in NAD(P)H dynamics. This suggests that the metabolic flux changes in primary metabolism during the cell cycle occur to satisfy the precursor and energy demands of the uncovered temporally segregated biosynthetic activities. Thus, we have revealed a key temporal aspect of the intracellular physiology during the cell cycle.

The uncovered two-wave behavior of protein biosynthesis activity opposes the current notion of its monotonic dynamics during the cell cycle. This notion has emerged from early studies using radioactive labeling^3–5^. In fact, a mathematical analysis of the key reference work^4^ showed that its method based on radioactive dual-labeling and centrifugal elutriation is unable to discriminate between exponential and periodic dynamics of protein synthesis rate^56^. More recent single-cell studies with microscopy and fluorescent proteins^6,7^ had also suggested that protein synthesis rate is monotonic. Here, it is interesting to note that while the authors of previous work^7^ claim that protein biosynthesis rate is constant during the cell cycle, one can clearly see in their data, a reproducible drop of the fluorescent-protein production rate around budding, which is one the aspects that led us to infer a non-monotonic behavior in the protein synthesis rate. Finally, based on a study from the Manalis laboratory that used a suspended microchannel resonator to determine yeast cell growth rate as a function of cell mass^57^, one could also conclude that protein synthesis rate would be constant during the cell cycle. Yet, it must be noted that the authors had performed linear regression in rather broad ranges of cell buoyant masses (>threefold change) and respective cell growth rates (>11-fold change), which are larger spreads than those during the cell cycle (~1.4-fold change of dry mass and ~1.6-fold change of dry mass derivative, as estimated from our data). The changes in protein synthesis rate during the cell cycle that we report here could thus be well hidden in the noise of the cell mass/growth rate data from the other work^57^. Moreover, cellular composition changes during the cell cycle could potentially confound a direct comparison of the buoyant-mass data^57^ with our dry-mass-related data (as shown in formula 1 in recent work^58^). The authors of the paper^57^ have cautiously not made any conclusion on cell-cycle dynamics of cell growth rate in yeast.

In contrast to the current notion on the protein synthesis dynamics during the cell cycle and in support of our work, an earlier study with glucose-limited chemostat cultures found that the rate of protein biosynthesis fell close to zero in the first half of the S phase^59^. A recent study employing an inertial picobalance and microscopy showed that the growth rate of yeast cells in S/G2/M has a non-monotonic pattern similar to the protein synthesis rate dynamics observed in this work^60^. Furthermore, TORC1 and PKA activity toward ribosome biogenesis was recently reported to have two waves per cell cycle^21^ and we found (with two completely orthogonal single-cell methods) that protein synthesis has two activity peaks during the cell cycle. This suggests that the existing notion of a constant/exponential protein biosynthesis rate during the cell cycle needs to be revised.

Our finding that the activity of lipid and cell-wall-polysaccharide biosynthesis changes during the cell cycle is in line with some indirect evidence from literature. Specifically, cells with a temperature-sensitive mutant of acetyl-CoA carboxylase Acc1, a crucial enzyme in fatty acid biosynthesis, were reported to be arrested in G2/M under a restrictive temperature^61^. Besides, the translational efficiency of messenger RNAs encoding lipogenic enzymes (Acc1, Fas1 and Fas2) as well as the transcription of the fatty acid elongase Elo2 involved in sphingolipid biosynthesis were found to increase in G2/M^11^. Two recent studies have demonstrated that a range of metabolites involved in pathways of lipid metabolism have peak abundance in S/G2/M^9,10^. For polysaccharides, an early study based on pulse-labeling electron microscopic experiments reported that in *Saccharomyces* *cerevisiae*, the rate of glucan and mannan biosynthesis increases after budding (S/G2/M) and drops at cytokinesis and in the pre-budding phase (G1)^62^. Thus, our results regarding the increased lipid and polysaccharide biosynthesis activity in S/G2/M are supported by a range of indications from literature.

One implication of our work is that we potentially should start looking at the concept of ‘cell growth’ in a different manner. While it is known that the rate of cell growth (in terms of cell mass or size) changes during the cell cycle^63,64^, here we show that the individual contributors to cell growth and mass (the biosynthetic processes synthesizing the different cellular components) are differentially active at different moments of the cell cycle. We have earlier shown this for G1, where protein biosynthesis rate is high and cell size growth is low^8^, but now we extend this to the whole cell cycle and other biosynthetic processes. While the concept of cell growth has been viewed holistically for decades, expanding the knowledge of cell-size and cell-cycle control, we now suggest going a step further to look at cell growth during the cell cycle in a more differentiated manner, where protein, lipid and polysaccharide biosynthesis as key contributors to cell growth are partially segregated in time.

Our work suggests that the uncovered temporal segregation in the biosynthetic processes is responsible for the observed metabolic dynamics during the cell cycle, where high glucose-uptake and fermentation fluxes occur in G1, followed by a switch to respiration at the onset of the S phase and eventual return to high fermentation toward mitotic exit. These dynamics in primary carbon and energy metabolism seem to be in place to meet the temporally changing demands in the biosynthetic processes. An early work based on glucose-limited synchronous cultures^59^ and a recent multi-omics study with α-factor-synchronized cells^9^ has generated important indications along these lines, but we can now (based on direct activity measurements) provide actual evidence to this notion. Together with the fact that we have observed metabolic oscillations under a broad range of experimental conditions whenever cells divided, this indicates that the metabolic oscillations do not emerge in specific primary metabolic pathways, such as respiration- or storage-related pathways, as earlier conjectured^34,49–53^. Thus, primary metabolism is dynamic likely because it has to fulfill the temporarily changing demands for precursors, redox and energy cofactors to supply the different biosynthetic processes.

The key question is now what causes this temporal segregation in biosynthesis. In the first instance, one would speculate that it is driven by the cell-cycle machinery, which indeed has targets in metabolism^65–69^; however, we and others have recently found that the metabolic oscillations in the range of hours, manifesting in NAD(P)H, ATP or flavin dynamics, also occur in cells that do not go through the cell cycle^44,55^, including cells undergoing dynamic depletion of the Cdc20 (ref. ^70^) or α-factor treatment^44^. This suggests that the biosynthetic/metabolic oscillations are (at least not primarily) generated by cell-cycle activity. We conjecture that negative feedback interactions between different biosynthetic processes could form a biosynthetic oscillator. Such negative feedback could be based on the competition for the resources from the primary metabolism or on the regulation of gene expression, for instance, by metabolite-dependent chromatin modification^71,72^. Alternatively, the biosynthetic dynamics could be orchestrated by the earlier suggested transcriptional oscillator^73^, by signaling pathways (for example TORC1/2, PKA and Snf1) sensing biomass precursor levels or by a mechanism overarching these diverse players.

## Methods

### Strains

An overview of *S.* *cerevisiae* strains used in this study is presented in Supplementary Table 1. The strains had the background of the prototrophic S288C-derived strain YSBN6 (MATa FY3 HO::HphMX4) or the auxotrophic S288C-derived strain YSBN10 (MATa FY3 HO::HphMX4, ura3-52)^74^. Sequences of primers used for strain construction are provided in Supplementary Table 2. To construct the strains YSBN6 Atp3–mCherry, YSBN6.tetO7–sfGFP, YSBN6 Ugp1–mCherry-AID and YSBN6.AIDcontrol (Supplementary Table 1), we implemented a number of cloning steps with the goal to insert a sequence of interest into a parental strain via homologous recombination. First, using Gibson assembly or phosphorylation ligation, we created a plasmid with *Escherichia* *coli* origin of replication as well as antibiotic selection marker in the backbone and with the sequence of interest accompanied by a yeast selection marker both flanked by the sequences for homologous recombination. The correctness of this plasmid assembly was checked with PCR and sequencing. Second, we linearized the plasmid by amplifying the fragment containing the flanking sequences and, between them, the sequence of interest with the yeast selection marker. Third, we transformed a target strain with the linear fragment using an established protocol^75^ and grew cells on a 2% glucose YPD agar plate with a selection agent (for example G418, nourseothricin). Fourth, resulting colonies were re-streaked on a replicate selection plate, and new colonies on it were inoculated in liquid selection YPD with 2% glucose to produce overnight cultures, from which genomic DNA (gDNA) was isolated and glycerol stock was made for long-term storage at −70/−80 °C. The integration of the sequence of interest was verified through PCR on gDNA and sequencing of this PCR amplicon.

To generate a strain with suppressed carbohydrate-storage biosynthesis, we knocked out four genes, namely, *TPS1*, *TPS2*, *GSY1* and *GSY2*, with the CRISPR/Cas9 system adapted from elsewhere^76^. To make the strain expressing Cas9 (YSBN6-Cas9), we integrated the *Can1Δ::cas9-natNT2* cassette amplified from the strain IMX585 (ref. ^76^) into YSBN6. In parallel, using pROS13 (ref. ^76^) as a basis, we created two plasmids each of which expresses two sgRNAs targeting the genes of interest. First, to have different selection markers in these plasmids, the kanMX cassette in pROS13 was replaced by the pAgTEF1-ble-tAgTEF1 cassette from pUG66 (ref. ^77^) conveying phleomycin resistance (the resulting plasmid was called pROS_phleo). Second, using the yeastriction webtool^76^, we designed primers that target each of the four genes of interest using the S288C genome as a template and, following the protocol from elsewhere^76^, introduced the corresponding sequences in the plasmids pROS13 and pROS_phleo, obtaining pROS13-Tps2/Gsy1 and pROS_phleo-Tps1/Gsy2. Subsequently, we transformed the YSBN6-Cas9 strain with pROS_phleo-Tps1/Gsy2 using phleomycin for selection. To avoid genetic heterogeneity, single colonies were later picked and re-streaked on a non-selective plate, from which single colonies were taken again to start liquid cultures for PCR verification of gene deletion and long-term storage of the strain. Eventually, after obtaining the YSBN6 ΔTps1ΔGsy2 strain, we transformed it with the pROS13-Tps2/Gsy1 plasmid using G418 for selection and, after colony re-streaking and PCR verification, obtained the desired strain YSBN6 ΔTps1ΔTps2ΔGsy1ΔGsy2. To generate the YSBN10 glycolytic biosensor, we incorporated pTEF7mut_CggRAla250 from Addgene plasmid 124585 (ref. ^47^) into the HO region of YSBN10 via CRISPR/Cas9 and co-transformed the cells with the reporter plasmid P_cggRO (Addgene 124582)^47^. YSBN10 control for the glycolytic biosensor was transformed only with the reporter plasmid. Plasmids to generate key strains are deposited on Addgene: pB_tetO7_sfGFP (196616), pUGP1.1 (196615), GA46 (196614), pROS13-Tps2/Gsy1 (196613) and pROS_phleo-Tps1/Gsy2 (196612); more details in Supplementary Tables 1 and 2.

### Liquid media

In this study, we used two minimal media, one of which was supplemented with biomass precursors in several experiments (specified below), and one complex medium. The first minimal medium was yeast nitrogen base medium without amino acids, referred to as YNB, which was prepared by dissolving 6.9 g of the powder (Formedium, CYN0410) in 1 l water. YNB was supplemented with 2% (20 g l^−1^) or 0.015% glucose (Millipore, 49159). The second minimal medium was modified Verduyn minimal medium^78^. We composed it using four stock solutions: 10× buffer solution, 5× salt solution, 100× tracer salt solution and 1,000× vitamin solution. The 10× buffer solution represented 100 mM solution of potassium phthalate monobasic (HOOCC_6_H_4_COOK, Sigma-Aldrich, 60360) in water with pH set to 5 with KOH (Fisher Scientific, 10113190). One liter 5× salt solution contained 25 g (NH_4_)_2_SO_4_ (Sigma-Aldrich, 09978), 15 g KH_2_PO_4_ (Sigma-Aldrich, P5655) and 2.5 g MgSO_4_·7H_2_O (Sigma-Aldrich, 63138) dissolved in water. The 1 l 100× tracer salt solution contained 2.135 g EDTA (Na_4_EDTA·2H_2_O, Sigma-Aldrich, ED4SS), 0.449 g ZnSO_4_·7H_2_O (Supelco, 1.08883), 0.031 g CoCl_2_·6H_2_O (Supelco, 1.02539), 0.099 g MnCl_2_·4H_2_O (Sigma-Aldrich, M5005), 0.03 g CuSO_4_·5H_2_O (Supelco, 1.02790), 0.45 g CaCl_2_·2H_2_O (Sigma-Aldrich, 223506), 0.297 g FeSO_4_·7H_2_O (Sigma-Aldrich, 215422; light-blue-green powder), 0.044 g NaMoO_4_·2H_2_O (Sigma-Aldrich, M1651), 0.1 g H_3_BO_3_ (Sigma-Aldrich, B7901) and 0.01 g KI (Sigma-Aldrich, 221945) dissolved in water (the solution was used while its color remained light-green and discarded when the color changed to light-red). One liter 1,000× vitamin solution contained 0.05 g d-biotin (Sigma-Aldrich, B4501), 1 g d-pantothenic acid hemicalcium salt (Sigma-Aldrich, 21210), 1 g nicotinic acid (Sigma-Aldrich, 72309), 25 g myo-inositol (Millipore, 57570), 1 g pyridoxine hydrochloride (Sigma-Aldrich, P9755), 0.2 g 4-aminobenzoic acid (Sigma, A9878) and 1 g thiamine hydrochloride (Sigma-Aldrich, T4625) dissolved in water. The modified Verduyn minimal medium was supplemented with appropriate carbon sources, which are indicated in Methods describing the experiments where this medium was used. The complex medium YPD was composed of 1% (10 g l^−1^) yeast extract (Difco, 212750), 2% (20 g l^−1^) peptone (Difco, 211677) and 1% glucose (Millipore, 49159) dissolved in water.

In the experiments where we determined the sfGFP production rate, used the stop-and-respond method, monitored the histone protein Hta2, traced cell volume and surface dynamics and applied the glycolytic flux biosensor, cells were cultivated in YNB with 2% glucose supplemented. In the 2-NBDG-addition experiments (Fig. 4e), cells were cultivated in 0.015% glucose YNB. In the experiments generating Fig. 5a–d, cells were cultivated in modified Verduyn medium with the addition of 1% glucose, 2% pyruvate or the combination of 1% glucose with LM (Lipid Mixture 1, Sigma, L0288) or with CSM (Formedium, DCS0019); cells were also cultivated in YPD with 1% glucose.

### Cultivation

Several days before an experiment, we recovered a necessary strain from its glycerol stock stored at −70/−80 °C by growing it for 2–3 d on a 2% glucose YPD agar plate. A small part of a single colony was picked from the plate and inoculated into 10 ml liquid medium in a 100-ml shake flask, initiating an overnight pre-culture. If we planned to eventually grow cells in the microfluidic device in a medium with 1% glucose or 2% pyruvate, this pre-culture was based on 1% glucose. Alternatively, if we planned to eventually grow cells in the microfluidic device in a medium with 2% glucose, the pre-culture was based also on 2% glucose. The pre-culture was grown overnight at 30 °C at a shaking speed of 300 r.p.m., with the pre-culture’s OD_600_ being in the morning of the next day typically <2, thus indicating an exponential state. If we planned to eventually grow cells in the microfluidic device in a medium with 1 or 2% glucose (high glucose), a new culture was started from the pre-culture by diluting it in the same fresh medium (10 ml in a 100-ml shake flask) at OD_600_ in the range 0.0125–0.05. This new culture was grown at 30 °C at a shaking speed of 300 r.p.m. for several hours and at OD_600_ in the range 0.08–0.2, cells were loaded in the microfluidic device as described previously^14,15^. Cultivating cells in 2% pyruvate and 0.015% glucose is described in Methods of respective experiments.

Before using a medium in a microfluidic experiment, we filtered and prewarmed it by shaking in a flask at 30 °C for at least 30 min. In the microfluidic device, cells were constantly provided with fresh medium by a syringe pump or an air-pressurized pumping system (OB1, Elveflow) assisted by a flow sensor (MFS2, Elveflow). While assembling the system that provides the medium to the microfluidic device, we took necessary precautions not to contaminate the medium (namely, working close to a Bunsen burner or in a laminar flow cabinet, disinfecting tubing with ethanol and drying it with compressed clean air). During cultivation in the microfluidic device, the temperature was maintained at 30 °C with the help of a microscope incubator (Life Imaging Services). Cells were kept in the microfluidic device under constant conditions by providing fresh medium for controlled periods of time. Methods describing individual experiments provide details on the media and their carbon-source supplementation, culturing scheme in shake flasks, the medium flow rate in the microfluidic device and media/oxygen-level switches that were used in these experiments. Conditions of the stop-and-respond experiments are summarized in Supplementary Table 3. Supplementary Table 8 summarizes the growth conditions among which cell-cycle-associated NAD(P)H oscillations were compared (Fig. 5a–d).

### Microscopy

The microfluidic device was mounted to one of two Nikon Eclipse Ti-E inverted wide-field fluorescence microscopes (microscope 1 and 2) where time-lapse imaging of cells was performed. Microscopes were equipped with Andor DU-897 EX cameras, ×40 (Nikon ×40 S Fluor Oil, NA = 1.3) and ×100 (Nikon ×100 S Fluor Oil Iris, NA = 1.30; Nikon Plan Apo VC Oil DIC N2, NA = 1.4) objectives. Microscope 1 was used with either CoolLED pE-2 (denoted as setup 1a) or Lumencor AURA (setup 1b) excitation system. Microscope 2 was always used with the CoolLED pE-2 excitation system (setup 2a). For NAD(P)H measurements, we excited cells at 365 nm in setups 1–2a and at 360 nm in setup 1b, employing a 350/50-nm band-pass filter, a 409-nm beam splitter and a 435/40-nm emission filter (NAD(P)H channel). For GFP measurements, we excited cells at 470 nm in setups 1–2a and at 485 nm in setup 1b, using a 470/40-nm band-pass filter, a 495-nm beam splitter and a 525/50-nm emission filter (GFP channel). For red fluorescent protein (RFP) measurements, we excited cells at 565 nm in setups 1–2a and at 560 nm in setup 1b, using a 560/40-nm band-pass filter, a 585-nm beam splitter and a 630/75-nm emission filter (RFP channel). For YFP measurements, we excited cells at 500 nm in setup 2a, using a 520/20-nm band-pass filter, a 515-nm beam splitter and a 535/30-nm emission filter (YFP channel). For bright-field imaging, a halogen lamp produced light that was filtered with a 420-nm beam splitter to exclude UV before illuminating cells (BF channel). The microscopes were operated using NIS-Elements software. We set the Readout Mode to 1 MHz to minimize the camera readout noise and fixed the baseline level of the cameras to 500 at −75 °C. The Nikon Perfect Focus System was used in time-lapse imaging to prevent the loss of focus set at the beginning of the experiment (in which a cell was seen as surrounded by two concentric circumferences of very low and high intensity pixels, respectively). In Methods sections dedicated to individual experiments, we specify the frequency of image acquisition, objective, setup and channels, indicating the corresponding percentage of maximal light intensity and exposure time.

### Image and signal analysis

In every microscopy experiment, multiple non-overlapping regions in the *XY* plane of the microfluidic device were imaged, which resulted in a set of Nikon NIS-Elements ND2 files each containing a multi-channel video for one *XY* region. Every ND2 file was imported into ImageJ^79,80^ where images in the fluorescent channels were background corrected via rolling-ball background subtraction plugin (except for the 2-NBDG addition experiment, see details in the respective Methods section) and images in the bright-field channel were sharpened and contrast-enhanced, after which the video was saved as a TIFF file. Cells were tracked throughout the video and segmented by fitting an ellipse in the bright-field image at each time point via the semi-automated plugin BudJ^81^ used with ImageJ. Simultaneously, by visual inspection and with the help of a custom macro, we recorded for each segmented cell the time points of budding events (appearance of a dark-pixel cluster from which a daughter cell would later grow) and death (abrupt shrinking and darkening of the cell, cessation of cytoplasmic movement, after which the data from the cell were not used). When a glycolytic flux biosensor was used, we also recorded the time points of cytokinesis events (one time point before the daughter cell would rapidly detach from the mother cell, accompanied by the appearance of a dark‐pixel line between the mother and daughter cells). To analyze cellular fluorescence data, we uploaded the video-containing TIFF file into a NumPy multidimensional array via Python’s module scikit-image^82^ and extracted the pixels corresponding to a cell of interest by overlapping the array with the segmentation ellipses provided by BudJ. To get a proxy of concentration, we calculated the average fluorescence intensity of the pixels in the cell segmentation. Cell volume and surface area were calculated using the radii of the segmentation ellipse provided by BudJ and assuming that a cell is a prolate spheroid. All data analysis and result visualization were implemented in Python. Methods sections dedicated to individual experiments and figure captions as well as Supplementary Table 4 and 8 describe further details of image and signal analysis.

### Detection of mitotic exit and START in Whi5 dynamics

Observing the localization of Whi5 tagged with a fluorescent protein (sfGFP, mGFP or mCherry), we identified the time points of the cell-cycle events of two kinds, namely, ME and START. Specifically, we calculated the ratio between the s.d. and mean of the pixel intensities in a cell segmentation (mother-cell compartment) at each time point of the video. Further, we automatically detected those time points before which this ratio’s derivative reaches its local maxima (ME) and minima (START) (Python’s method of scipy.signal.argrelextrema with *x* time points on each side to compare, where *x* = 12 if *δt* = 6 or *x* = 24 if *δt* = 3). To exclude wrongly identified events and add missing ones, we visually inspected the single-cell traces of the ratio, having the knowledge that ME precedes START followed by budding and that the time period between budding and ME is usually bigger than that between ME and budding. In some cell cycles, it was impossible to identify ME and START due to noise in the ratio.

### Tracing cell volume, surface area and sfGFP production rate

To study the cell volume, cell surface area and the production rate of sfGFP during the cell cycle, we microscopically monitored the strain YSBN6.tetO7-sfGFP (Supplementary Table 1) with tetO7-sfGFP-KanMX in HO locus and Whi5-mCherry-BLE. This strain was cultivated in the microfluidics device, with the syringe pump continuously providing 2% YNB at the 4–5 µl min^-1^ flow rate. In the first experiment (the first replicate in Fig. 1a and Extended Data Fig. 1f as well as Figs. 1b and 2b and Extended Data Figs. 1g and 4), microscopy was performed every δt = 6 min with the setup 1b, ×100 objective and in the following channels: BF (3 V, 50 ms), GFP (2%, 100 ms), RFP (10%, 600 ms) and NAD(P)H (4%, 200 ms). In the second and third experiments (the second and third replicates in Fig. 1a and Extended Data Fig. 1f), microscopy was performed in the same way, with the exception of no NAD(P)H measurement.

To work with a continuous cell volume trace $V\left( t \right)\{ fl\}$ without the abrupt drop corresponding to cytokinesis, we considered a cell cycle to be confined within two MEs, excluding the first but including the last one: $t \in \left( {{\mathrm{ME}}_i,{\mathrm{ME}}_{i + 1}} \right]\{ \min \}$. Excluding the first ME is motivated by the fact that cytokinesis happens soon after it. In general, the cell volume *V* consists of the mother *V*^m^ and daughter *V*^d^ parts. We calculated *V*^m^ and *V*^d^ separately, using the radii of the ellipse that ImageJ’s plugin BudJ fitted to the mother and daughter compartments (also referred to as cells) in the bright-field image. Specifically, we assumed that the mother and daughter cells are prolate spheroids, therefore, *V*^m^ and *V*^d^ are calculated via $\frac{4}{3}\pi Rr^2$, where *R* and *r* are the major and minor radii, respectively. Given microscope resolution, it was infeasible to accurately segment daughter cells with BudJ for some time after budding (2–4 time points, 12–24 min, on the median level in the replicate experiments). In the corresponding time points, the daughter cell volume was reconstructed using linear interpolation between the zero volume at budding and the first volume calculation on the basis of BudJ-derived radii. Eventually, a cell-cycle trace of the cell volume was assembled as follows: $V\left( t \right) = V^{\mathrm m}\left( t \right) + V^{\mathrm{d}}\left( t \right),t \in \left( {{\mathrm{ME}}_i,{\mathrm{ME}}_{i + 1}} \right]$, with *V*^d^(*t*) equal to zero until budding (Extended Data Fig. 1a).

Next, we smoothed the cell volume to filter out local fluctuations caused by imperfect segmentation and to capture visible global behavior (Extended Data Fig. 1a). To support smoothing at the beginning and end of a cell-cycle trace, we used the data in the adjacent 50 min of the preceding and following cell cycles (if there were such data), geometrically translating the cell volume down and up to abolish the discontinuity caused by cytokinesis:

$$V\left( t \right),t \in \left( {{\mathrm{ME}}_i - 50,{\mathrm{ME}}_{i + 1} + 50} \right] = \left\{ {\begin{array}{*{20}{l}} {V^{{\mathrm{down}}}\left( t \right),t \in \left( {{\mathrm{ME}}_i - 50,\,{\mathrm{ME}}_i} \right]} \hfill \\ {V\left( t \right),t \in \left( {{\mathrm{ME}}_i,{\mathrm{ME}}_{i + 1}} \right]} \hfill \\ {V^{\mathrm{up}}\left( t \right),t \in \left( {{\mathrm{ME}}_{i + 1},{\mathrm{ME}}_{i + 1} + 50} \right]} \hfill \end{array}} \right..$$

Particularly, in the preceding cell cycle, we subtracted the daughter cell volume at ME: *V*^down^(*t*) = *V*(*t*)*−V* ^d^ (*ME*_*i*_), *t*∈(ME_*i*_−50, ME_i_]. Similarly, in the following cell cycle, we added the daughter cell volume at ME belonging to the cell cycle of interest: $V^{\mathrm{up}}\left(t \right) = V\left( t \right) + V^{\mathrm{d}}\left( {{\mathrm{ME}}_{i + 1}} \right),t \in \left( {{\mathrm{ME}}_{i + 1},{\mathrm{ME}}_{i + 1} + 50} \right]$. We smoothed the cell volume $V\left( t \right) \to V^{\mathrm{smooth}}\left( t \right),t \in \left( {{\mathrm{ME}}_i - 50,{\mathrm{ME}}_{i + 1} + 50} \right]$ with the LOWESS method selecting the time window size of line fitting individually in each cell cycle based on visual inspection of the smoothing quality. The selected window sizes for LOWESS were equal to 6 on the median level in the replicate experiments and analyses. To present the cell volume dynamics, we extracted the values of $V^{\,\mathrm{smooth}}\left( t \right)$ in the interval $t \in [{\mathrm{ME}}_i,{\mathrm{ME}}_{i + 1}]$.

The cell surface area *S* was also perceived as the sum of the mother and daughter cell surface areas: $S^m + S^d$, each of which was calculated according to the prolate spheroid assumption: $2\pi r^2\left( {1 + \frac{R}{{re}}\arcsin e} \right)$, where $e = \sqrt {1 - \frac{{r^2}}{{R^2}}}$ and *R* and *r* are the major and minor radii, respectively. We tackled the discontinuity caused by cytokinesis and interpolated the data points after budding analogously to processing the cell volume. The data were smoothed $s\left( t \right) \to S^{\mathrm{smooth}}\left( t \right),t \in \left( {{\mathrm{ME}}_i - 50,{\mathrm{ME}}_{i + 1} + 50} \right]$ by applying LOWESS with the window size equal to eight time points in all analyzed cell cycles. To obtain the derivative of the cell surface area, we differentiated the cubic spline that goes through the points of $S^{\mathrm{smooth}}\left( t \right),t \in \left( {{\mathrm{ME}}_i - 50,{\mathrm{ME}}_{i + 1} + 50} \right]$ and extracted the values in the interval $t \in [{\mathrm{ME}}_i,{\mathrm{ME}}_{i + 1}]$.

We assumed that there is no active degradation of unfused fluorescent proteins and, therefore, calculated the production rate of sfGFP $r_{\mathrm {sfGFP}}(t)$ directly by differentiating its abundance and considering the maturation kinetics of the fluorescent protein. To obtain for this purpose a cell-cycle trace of sfGFP abundance $A_{\mathrm{sfGFP}}(t)$ (Extended Data Fig. 1c), we multiplied two smoothed traces, namely: (1) of sfGFP fluorescence averaged across the mother-cell pixels $F_{\mathrm{sfGFP}}^{\,\mathrm{smooth}}(t)$ (Extended Data Fig. 1b); and (2) of cell volume $V^{\,\mathrm{smooth}}(t)$ (Extended Data Fig. 1a). In each replicate experiment, we normalized the sfGFP fluorescence traces by the average fluorescence across all cell-cycle traces. We smoothed the cell-cycle trace of sfGFP fluorescence together with the data from the adjacent cell cycles using the LOWESS method: $F_{\mathrm{sfGFP}}\left( t \right) \to F_{\mathrm{sfGFP}}^{\,\mathrm{smooth}}\left( t \right),t \in \left( {{\mathrm{ME}}_i - 50,{\mathrm{ME}}_{i + 1} + 50} \right]$ (Extended Data Fig. 1b). The sfGFP fluorescence is a continuous readout, unaffected by cytokinesis, therefore, we did not pre-process $F_{\mathrm{sfGFP}}\left( t \right),t \in \left( {{\mathrm{ME}}_i - 50,{\mathrm{ME}}_{i + 1} + 50} \right]$ by translating the data from the adjacent cell cycles up and down as we did with the cell volume and surface area. The individually selected window sizes for the LOWESS smoothing were equal to 6–8 on the median level in the replicate experiments. To obtain the first and second derivatives of the cell-cycle trace of sfGFP abundance, we differentiated the cubic spline that goes through the points of $A_{\mathrm{sfGFP}}\left( t \right),t \in \left( {{\mathrm{ME}}_i - 50,{\mathrm{ME}}_{i + 1} + 50} \right]$ and extracted the values in the interval, $t \in [{\mathrm{ME}}_i,{\mathrm{ME}}_{i + 1}]$. To account for sfGFP maturation kinetics while calculating the sfGFP production rate, we used the model described previously^83^ and assumed the sfGFP maturation half-time *t*_1/2_ = 6 min^84^: $r_{\mathrm{sfGFP}}(t) = \frac{{t_{1/2}}}{{ln2}} \cdot \frac{{d^2A_{\mathrm{sfGFP}}\left( t \right)}}{{dt^2}} + \frac{{dA_{\mathrm{sfGFP}}\left( t \right)}}{{dt}}$, where $r_{\mathrm{sfGFP}}(t)$ is the sfGFP production rate (Extended Data Fig. 1d). Negative values appearing at some time points in several cell-cycle traces (Fig. 1a) likely originate from measurement noise propagated by the calculation of two successive derivatives required to obtain the maturation-corrected sfGFP production rate. Without accounting for sfGFP maturation, we used the first derivative of the cell-cycle trace of sfGFP abundance $A_{\mathrm{sfGFP}}\left( t \right)$ as the production rate of this protein: $r_{\mathrm{sfGFP}}(t) = \frac{{dA_{\mathrm{sfGFP}}\left( t \right)}}{{dt}}$ (Extended Data Fig. 1e).

To align several cell-cycle traces and to calculate the phase, we used an array of four cell cycle events *E* = {ME, START, BUD, next ME} as reference points. Specifically, we computed the average cell-cycle-relative timing for each of these events $\bar \varphi ^e$ (vertical lines in Figs. 1a,b and 2b and Extended Data Figs. 1f,g and 4) in the following way: $\forall e \in E,\ {\bar\varphi} ^e = \frac{1}{N}\mathop {\sum}\nolimits_{cc = 1}^N {\frac{{t_{cc}^{\,e} - t_{cc}^{\mathrm{ME}}}}{{t_{cc}^{\mathrm{next}\,\mathrm{ME}} - t_{cc}^{\mathrm{ME}}}}}$, where $t_{cc}^{\,e}$ is the time in minutes when the event *e* happens in the cell cycle *cc* and *N* is the number of cell cycles. In the aligned cell cycles, we converted the time in minutes *t* to the phase *φ*_*cc*_ in the following way: $\varphi _{cc}(t) = (\bar \varphi ^{E\left[ {i + 1} \right]} - \bar \varphi ^{E[i]})\frac{{t - t_{cc}^{\,E[i]}}}{{t_{cc}^{\,E[i + 1]} - t_{cc}^{\,E[i]}}} + \bar \varphi ^{E[i]}$ for $t \in \left[ {t_{cc}^{\,E\left[ i \right]},t_{cc}^{\,E\left[ {i + 1} \right]}} \right]$ if *E* [*i*] = ME or $t \in \left( {t_{cc}^{\,E\left[ i \right]},t_{cc}^{\,E\left[ {i + 1} \right]}} \right]$ if *E* [*i*] ≠ ME, where *i* is the index number of an event in the array *E*. To interpret the relative phase values and eventually compare Fig. 1a to 1d, one can multiply the phase values by provided average cell-cycle durations, thus obtaining the phase values expressed in minutes, in the same scale as in Fig. 1d.

To summarize several individual cell-cycle traces and obtain an average pattern during the cell cycle, we regressed the values belonging to different traces and time points $$\left\{ {V^{\mathrm{smooth}}\left( {t,cc} \right)} \right\}/$$$$\left\{ {\frac{d}{{dt}}S^{\mathrm{smooth}}\left( {t,cc} \right)} \right\}/\left\{ {r_{\mathrm{sfGFP}}\left( {t,cc} \right)} \right\}$$ against the respective cell-cycle phases $$\{ \varphi _{cc}(t)\}$$. Specifically, we implemented a Gaussian process regression (Python’s sklearn.gaussian_process), using as a prior an RBF kernel and a white kernel, and maximizing the log-marginal likelihood. The Gaussian process regression ignored the connection of data points between adjacent phases, therefore, we attributed all the variability at each phase to measurement noise.

To build heat maps (Fig. 1b and Extended Data Fig. 1g), we interpolated each cell-cycle trace of sfGFP production rate (line-connected markers of the first replicate experiment in Fig. 1a and Extended Data Fig. 1f) using a cubic spline and collected from it values at 17 evenly spaced phase points making up a new trace $r(\varphi )$. These values of sfGFP production rate were converted to have the minima and maxima fixed to 0 and 1, respectively, via $\frac{{r(\varphi ) - {{{\mathrm{min}}}}(r(\varphi ))}}{{\max \left( {r(\varphi )} \right) - {{{\mathrm{min}}}}(r(\varphi ))}}$.

### Stop-and-respond experiments

Multiple *XY* regions of the microfluidic device were microscopically observed in the bright-field, NAD(P)H, GFP and RFP channels with the time interval *δt* of 6 min via the microscopy setup 1b and ×100 objective. We provided cells in the microfluidic device with a medium allowing normal growth (control medium) for several hours and afterwards switched it to the same medium that additionally contained a compound leading to a metabolic perturbation (perturbation medium) (Supplementary Table 3).

To precisely control the flow rate in the microfluidic device, we used an air-pressurized pumping system (OB1, Elveflow) together with a flow sensor (MFS2, Elveflow). Medium replacement was performed automatically with a microfluidic flow switch matrix (MUX distributor, Elveflow). The control medium was usually provided at the flow rate 5 µl min^−1^ (in the first hours of some experiments the flow rate was reduced up to 3.6 µl min^−1^ to retain more cells in the microfluidic device). The media switch and the provision with the perturbation medium were always conducted at the flow rate 5 µl min^−1^. The time of the arrival of the perturbation medium into the microfluidic device was calculated by multiplying the flow rate and the total inner volume of the system providing the medium (the combination of the flow switch matrix, flow sensor and tubing with the length similar among different experiments). In case of CYH and NAA addition, the microscopy time point of or right after the arrival of the perturbation medium was confirmed by observing a sharp drop of NAD(P)H fluorescence. For convenience, we called the first time point of microscopy equal to or right after the calculated time of the perturbation medium arrival as *t*_switch_. In some experiments, the perturbation medium arrived to the microfluidic device during imaging so that some *XY* regions had *t*_switch_ one *δt* bigger that others (in case of CYH and NAA addition, it was seen in the dynamics of NAD(P)H signal averaged within individual *XY* regions). In an independent experiment with the shift to a fluorescent dye (C368, Thermo Fisher), we found that, having arrived in the microfluidic device, the new medium fully replaces a previous one within approximately 10 min. Thus, at *t*_switch_ and sometimes also at the next time point of microscopy, the actual concentration of a perturbation compound in the microfluidic device is likely lower than stated in Supplementary Table 3.

To infer the cell-cycle activity pattern of an inhibited process, we implemented the following data analysis pipeline:

1. In the microscopy movie, we traced the maximal number of cells that budded at least twice in the control medium and remained after *t*_switch_, which constituted the initial set of cells *S*0. With a custom macro in ImageJ, for each cell $c \in S0$, we recorded the time points of budding events and stored them in the sorted array $L_{\mathrm{BUD}}\left( c \right) = [t_{\mathrm{BUD}}^1(c), \ldots ,t_{\mathrm{BUD}}^n(c)]$, $|L_{\mathrm{BUD}}\left( c \right)| \ge 2$.
2. To decrease the uncertainty in aligning the single-cell responses to perturbation along the cell cycle, we focused the analysis on the predominant subpopulation of fast-dividing cells. Specifically, we constructed the distribution of the time duration between two latest budding events before *t*_switch_. For further analysis, we selected the cells within the interval of the median ± s.d. of this distribution, which resulted in a smaller set of cells *S*1.
3. For each cell $c \in S1$, via the semi-automatic procedure described above, we obtained the sorted arrays with the time points of ME and START events: $L_e\left( c \right),|L_e\left( c \right)| \ge 0$, $\forall e \in \{{\mathrm{ME}},{\mathrm{START}}\}$.
4. To identify the average relative timing of the cell-cycle events during normal growth, we studied the position of ME and START between two latest budding events before *t*_switch_. Specifically, defining that ME happens at 0 min of the cell cycle, we calculated the average relative timing of START and budding in minutes, $\Delta \bar t_{\mathrm{START}}$ and $\Delta \bar t_{\mathrm{BUD}}$, in the analyzed cells of *S*1 (by definition, $\Delta \bar t_{ME} = 0$). We also used the difference between the two latest budding events to get the average cell cycle duration $\Delta \bar t_{CC}$.
5. To decrease the contribution of measurement noise to the activity pattern, we smoothed the single-cell NAD(P)H traces: from $F_{\mathrm{raw}}^c\left( t \right)$ to $F_{\mathrm{smooth}}^c\left( t \right),t \in \{ t_{\min}^c + (i - 1)\delta t\} _{i = 1}^{n^c}$, where $t_{\min}^c$ is the first time point in minutes when the cell *c* was imaged and *n*^*c*^ is the total number of time points it was imaged. Specifically, we applied the Savitzky–Golay filter with the window length 7 and the third order of the polynomial used to fit the raw data (Python’s method scipy.signal.savgol_filter). An advantage of this filter for smoothing the NAD(P)H oscillations is that the resulting function minutely follows rather than severely cuts away the protruding data points of the crest and trough (Fig. 1c). In case of CYH and NAA addition, which caused a sharp drop of NAD(P)H fluorescence at the media switch, we broke each trace in two pieces, before and after the media switch, and smoothed them separately to prevent biasing the filter. To assist smoothing at the edges of the two pieces, we considered as if there were three more data points at each edge with the same value (the method’s mode = ‘nearest’). Once smoothing was conducted in two separate pieces of the single-cell trace, they were eventually merged back. We calculated the derivative value for each pair of adjacent data points in the smoothed NAD(P)H trace and assigned it to the middle between the corresponding time frames: $\dot F_{\mathrm{smooth}}^c(t),t \in \{ t_{\min}^c + (i - 0.5)\delta t\} _{i = 1}^{n^c - 1}$.
6. In each experiment, we identified the first time point of severe metabolic perturbation *t*_*p*_ at which the normal population-averaged dynamics of both NAD(P)H and growth rate sharply changed (Supplementary Table 4). Subsequently, we focused on the single cells’ NAD(P)H derivative values immediately preceding *t*_*p*_, $\{ P^{\,c}\} _{c \in S1},P^{\,c} = \dot F_{\mathrm{smooth}}^{\,c}\left( {t_p - 0.5\delta t} \right)$, to detect in them the cell-cycle-dependent response to the perturbation. To align the derivative values of this set along the cell cycle, for every cell *c*, we measured $\{ \theta _e^c\} _e$, the time periods passed from the latest cell cycle event of each kind $e \in \{{\mathrm{ME}},{\mathrm{START}},{\mathrm{BUD}}\}$ till $t_p - 0.5\delta t$. Considering that the closest event would describe the position of cell *c* in the cell cycle most reliably, we picked the smallest of these three time periods and adjusted it to match ME as the beginning of cell cycle: $\varphi ^c = \min\left( {\{ \theta _e^c\} _{e \in \left\{ {\mathrm{ME}},{\mathrm{START}},{\mathrm{BUD}} \right\}}} \right) + \Delta \bar t_{E^c}$ with $E^c = \arg \min _e\left( {\{ \theta _e^c\} _{e \in \left\{ {\mathrm{ME}},{\mathrm{START}},{\mathrm{BUD}} \right\}}} \right)$. To ensure that we are processing cells that had normal cell cycle dynamics before *t*_*p*_ (for example, no cell cycle arrest or slowdown), we excluded the cell *c* from the analysis if its cell cycle event closest to perturbation was abnormally delayed, specifically, if $\varphi ^c > \Delta \bar t_{\mathrm{START}} \cap E^c = {\mathrm{ME}}$, $\varphi ^c > \Delta \bar t_{\mathrm{BUD}} \cap E^c = {\mathrm{START}}$ or $\varphi ^c > \Delta \bar t_{CC} \cap E^c = {\mathrm{BUD}}$. Therefore, we arrived to a smaller or the same set of cells $S2,\left| {S2} \right| \le \left| {S1} \right|,$ for which we associated the NAD(P)H derivative value *P*^*c*^ with the position in cell cycle *φ*^*c*^ when perturbation happened. Besides, for each cell $c \in S2$, we stored the information about the kind of the cell-cycle event closest to perturbation, *E*^*c*^.
7. The NAD(P)H derivative value *P*^*c*^ is thought to contain not only the NAD(P)H response to perturbation but also the normal dynamics characteristic to the corresponding position in cell cycle *φ*^*c*^. Thereby, to obtain the NAD(P)H response to perturbation *R*^*c*^, we subtracted from *P*^*c*^ the median value of NAD(P)H derivative at the comparable time in the preceding cell cycle: $P^{\,c} - N(\varphi ^c)$, where
  $$\begin{array}{l}N\left( \phi \right) = \\\left\{ {\begin{array}{*{20}{l}} {{\mathrm{median}}\left( {\{ \dot F_{\mathrm{smooth}}^x(L_{ME}\left( x \right)_{\left[ { - 2} \right]} + \phi )\} _{x \in S1}} \right),0 < \phi \le \Delta \bar t_{\mathrm{START}}} \hfill \\ {{\mathrm{median}}\left( {\left\{ {\dot F_{\mathrm{smooth}}^x\left( {L_{\mathrm{START}}\left( x \right)_{\left[ { - 2} \right]} + \phi - \Delta \bar t_{\mathrm{START}}} \right)} \right\}_{x \in S1}} \right),} \hfill\\{\Delta \bar t_{\mathrm{START}} < \phi \le \Delta \bar t_{\mathrm{BUD}}} \hfill \\ {{\mathrm{median}}\left( {\left\{ {\dot F_{\mathrm{smooth}}^x\left( {L_{\mathrm{BUD}}\left( x \right)_{\left[ { - 2} \right]} + \phi - \Delta \bar t_{\mathrm{BUD}}} \right)} \right\}_{x \in S1}} \right),} \hfill\\{\Delta \bar t_{\mathrm{BUD}} < \phi \le \Delta \bar t_{CC}} \hfill \end{array}} \right.\end{array}$$
  with $L_e\left( x \right)_{\left[ { - 2} \right]}$ being the time of the second latest cell cycle event *e* before *t*_switch_. We assume that this subtraction reveals the actual NAD(P)H response to perturbation, by removing the contribution of normal NAD(P)H dynamics during the cell cycle.
  As the NAD(P)H response to perturbation is considered as a proxy for metabolic activity which should be expressed in non-negative values, we changed the sign of the obtained difference if *P*^*c*^ is on average negative: $\alpha \left( {P^{\,c} - N\left( {\varphi ^c} \right)} \right)$, where *α* = −1 if population-average NAD(P)H drops at *t*_*p*_ (CYH addition), and *α* = 1 otherwise. Thus, NAD(P)H response to perturbation is always, on average, a positive value.
  To make the value of the NAD(P)H response to perturbation comparable to the normal dynamics of NAD(P)H, we related the difference $P^{\,c} - N(\varphi ^c)$ to the amplitude of $N\left( \phi \right)$ (the amplitude of NAD(P)H oscillation): $R^c = \frac{{\alpha \left( {P^{\,c} - N\left( {\varphi ^c} \right)} \right)}}{{\mathop{\max}\limits_{\phi \in \left( {0,\Delta \bar t_{CC}} \right]}N\left( \phi \right) - \mathop{\min}\limits_{\phi \in \left( {0,\Delta \bar t_{CC}} \right]}N\left( \phi \right)}}$.
8. To obtain the desired cell-cycle activity pattern of the inhibited process, we regressed the single cells’ values of NAD(P)H response to perturbation $\{ R^c\} _{c \in S2}$ against the corresponding positions in cell cycle $\{ \varphi ^c\} _{c \in S2}$ when these cells had experienced the perturbation. Specifically, we implemented a Gaussian process regression (Python’s sklearn.gaussian_process), using as a prior an RBF kernel with the length scale range $[2\delta t,5(or\,6)\delta t]$ and a white kernel with the free noise level, and maximizing the log-marginal likelihood.

### Identification of the cell-cycle phase of karyokinesis

The strain YSBN6 Hta2-mRFP1 (Supplementary Table 1) was cultivated in 2% glucose YNB. Microscopy details were setup 2a; ×100 objective; BF (3 V, 50 ms, EM gain 1), GFP (20%, 200 ms, EM gain 3), RFP (10%, 100 ms, EM gain 3); and time step *δt* = 3 min. The syringe pump was used to maintain the flow rate of 2.4 μl min^−1^ in the microfluidic device.

We identified the boundaries of the mother cell in the bright-field image via BudJ cell segmentation and analyzed the pixels of the corresponding red fluorescence image (Extended Data Fig. 2a). Particularly, we implemented the local thresholding in which a pixel *i* is selected if $F_i > \overline {F_i} \left( {15 \times 15} \right) + p30$, where *F*_*i*_ is the pixel’s intensity, $\overline {F_i} \left( {15 \times 15} \right)$ – the mean intensity among the nearest 15 × 15 pixels (roughly, a third of the mother cell) and *p*30 – the thirtieth percentile among the mother cell’s pixels (Python’s method skimage.filters.threshold_local with block_size = 15, method = ‘mean’, offset = p30 and mode = ‘nearest’). Using the offset *p*30 helps to discard a small number of cytoplasmic pixels that due to noise happen to be brighter than their neighbors. For the same purpose, after the local thresholding, we removed objects (ensembles of selected pixels) smaller than 25 pixels and having the connectivity equal to 1 (two pixels are connected by one orthogonal step). Eventually, we segmented one object containing the brightest pixels, which represents nucleus. If some pixels inside this object were not selected in the local thresholding (due to noise, some single pixels may be dimmer), then we added these pixels to the selection. To calculate the abundance of the fusion Hta2-mRFP in the mother-cell nucleus, we summed the intensities of the pixels located within the segmented nucleus. Microscopy image processing was performed in Python with the help of the module skimage.

### Mathematical modeling of metabolism

The mathematical model describing the dynamics of cell mass during the cell cycle and inferring the absolute units for the biosynthetic rates by solving an optimization problem was built and implemented via General Algebraic Modeling System (GAMS) (GAMS Development Corporation) with the help of the solver ANTIGONE^85^. Supplementary Methods and Supplementary Table 6 provide a detailed description of the model with literature and experimentally derived parameter values.

To infer the fluxes of the core carbon and energy metabolism, we modified and simulated a previously developed thermodynamic-stoichiometric model of yeast metabolism^45^. Specifically, we split the model’s existing biomass equation into six separate equations, respectively defining the production of proteins, lipids, cell-wall polysaccharides, storage polysaccharides, RNA and DNA (Supplementary Table 7), which was conducted in accordance with the original formulation of this biomass equation^86^. Next, we introduced a new biomass production equation that combines these six major biomass components into biomass as the end product (Supplementary Table 7; biomass reaction). Regression analysis to determine the parameters of the modified model (standard Gibbs energies of formation and reactions) was performed in GAMS using the global optimization solver ANTIGONE^85^ as described previously^45^, where metabolomics and physiology data from elsewhere^40^ were employed. To estimate the formation Gibbs free energies of the major biomass components, we introduced them as estimated parameters to the regression problem, using the following equations:

$$\begin{array}{l}j \in \left\{ {\mathrm{proteins}},{\mathrm{lipids}},{\mathrm{polysacch}},{\mathrm{DNA}},{\mathrm{RNA}},{\mathrm{storage}} \right\}{{{\mathrm{and}}}}\\ \beta \in \left\{ {\begin{array}{*{20}{c}} {\mathrm{protein}}\,{\mathrm{reaction}},{\mathrm{lipid}}\,{\mathrm{reaction}},{\mathrm{polysaccharide}}\,{\mathrm{reaction}}, \\ {\mathrm{DNA}}\,{\mathrm{reaction}},{\mathrm{RNA}}\,{\mathrm{reaction}},{\mathrm{storage}}\,{\mathrm{reaction}} \end{array}} \right\}:\\ {{\Delta }}_fG_{\mathrm{biomass}}^{\prime 0} = \mathop {\sum}\limits_j {{{\Delta }}_fG_j^{\prime 0}} ,\\ {{\Delta }}_rG_\beta ^{\prime 0} = {{\Delta }}_fG_j^{\prime 0} + \mathop {\sum}\limits_{m \ne j} {S_{m,\beta }{{\Delta }}_fG_m^{\prime 0},j\,{\mathrm{is}}\,{\mathrm{produced}}\,{\mathrm{in}}\,\beta } ,\\ {{\Delta }}_rG_{\mathrm{biomass}\,\mathrm{reaction}}^\prime = 0\end{array}$$

where ${{\Delta }}_fG_{\mathrm{biomass}}^{\prime 0}$, ${{\Delta }}_fG_j^{\prime 0}$ and ${{\Delta }}_fG_m^{\prime 0}$ are the standard Gibbs energies of formation of biomass, the biomass component *j* and the metabolite *m*; ${{\Delta }}_rG_\beta ^{\prime 0}$ and ${{\Delta }}_rG_{\mathrm{biomass}\,\mathrm{reaction}}^\prime$ are the standard Gibbs energies of the reaction *β* and the biomass reaction; *S*_*m*,*β*_ is the stoichiometric coefficient of the metabolite *m* in the reaction *β*. Besides, in our modified model, we changed the Gibbs energy balance^45^, by using the formation Gibbs energies of the major biomass components instead the formation Gibbs energy of biomass.

FBA on the basis of this parameterized model was implemented in Python using the global optimization solver Gurobi^87^. To model primary metabolism during the cell cycle, we used the momentary relative contributions of biosynthetic processes to the total biomass production (Fig. 3d) to define the stoichiometric coefficients of the respective components in the biomass reaction in a cell-cycle-dependent manner. Specifically, for each of 17 discrete moments during the cell cycle $t \in T = \left\{ {T_i} \right\}_{i = 1}^n,\delta t = 6\,\min ,T_1 = 3\,\min ,\,T_n = 99\,\min$, we defined the biomass reaction as follows:

$$\begin{array}{l}\frac{{c_s^{\mathrm{proteins}}(t)}}{{c_{\mathrm{model}}^{\mathrm{proteins}}}}{\mathrm{proteins}} + \frac{{c_s^{\mathrm{lipids}}(t)}}{{c_{\mathrm{model}}^{\mathrm{lipids}}}}{\mathrm{lipids}} + \frac{{c_s^{\mathrm{polysacch}}(t)}}{{c_{\mathrm{model}}^{\mathrm{polysacch}}}}{\mathrm{polysacch}} + \frac{{c_s^{\mathrm{DNA}}(t)}}{{c_{\mathrm{model}}^{\mathrm{DNA}}}}{\mathrm{DNA}}\\ + \frac{{c_s^{\mathrm{RNA}}(t)}}{{c_{\mathrm{model}}^{\mathrm{RNA}}}}{\mathrm{RNA}} < = > ,\end{array}$$

where $c_s^{\,j}\left( t \right)j \in \{ \mathrm {proteins,lipids,polysacch,DNA,RNA}\}$ is the momentary relative contribution of the biosynthetic process of component *j* to the total biomass production in the cell-mass model trained on the set *s* of replicate measurements of biosynthetic activities (in total nine sets; more details are provided on the sets at the end of Supplementary Methods), $c_{\mathrm{model}}^{\,j}$ is the mass fraction of the component *j* regarding biomass in the model (Supplementary Table 7). Because there is only minor production of trehalose and glycogen under the high-glucose condition investigated in this study^29–31^, we set the coefficient of storage to zero throughout the cell cycle.

For each $t \in T$, we ran nine FBA simulations corresponding to different sets of experimental measurements used to train the cell-mass model (end of Supplementary Methods) and thus using slightly different values of the coefficients in the biomass reaction (as per the reaction above). In each simulation, we maximized the flux through the respectively defined biomass reaction, while using glucose as the sole carbon and energy source, and constraining the cellular Gibbs energy dissipation rate by the upper limit of $12.3\,\mathrm{J}\,\mathrm{gDW}^{ - 1}\mathrm{h}^{ - 1}$ (ref. ^45^). Contrarily to previous work^45^, we did not use population-level data to set bounds for variables (metabolite concentrations and Gibbs energies of reactions) to prevent biasing the simulations during the cell cycle. As an exception, we set the lower bound of cytoplasmic fdp to exp(−10.058) mol and the upper bound of cytoplasmic glyc3p to exp(−8.794) mol as in few individual simulations (in few combinations of *t* and *s*) without these bounds the reaction direction of fructose 1,6-bisphosphatase and glycerol kinase was different from expectations for high-glucose cultivation. Toward identifying optimal solutions in the large non-convex and non-linear solution space, we used the global optimization solver Gurobi 9.5.1 (ref. ^87^). The constraints on the formation energy of the biomass components and biomass were relaxed from the values estimated by the regression (±100 kJ mol^−1^) to account for potential variability in the organization of the macromolecules during the cell cycle. The objective function was extended to minimize the difference between the predicted formation energy of biomass components from the regression and the estimated values from the cell-cycle-specific FBA.

After the FBA simulations, we integrated the inferred fluxes with the total biomass synthesis rate (Fig. 3c), thus translating the FBA-associated flux units $\mathrm {mmol}\,(\mathrm{mmol}\,\mathrm{biomass})^{ - 1}\mathrm{h}^{ - 1}$ to the absolute flux units $\mathrm {pmol}\,\mathrm{cell}^{ - 1}\,\mathrm{h}^{ - 1}$:

$$\begin{array}{l}v_{i,s}^{\mathrm{cell}}\left( t \right)\frac{{\mathrm{pmol}}}{{\mathrm{cell} \cdot h}} = 10^{ - 3} \cdot \frac{{v_{i,s}^{\mathrm{FBA}}\left( t \right)\frac{{\mathrm{mmol}}}{{\mathrm{mmol}\,\mathrm{biomass} \cdot h}}}}{{v_{\mathrm{biomass},{s}}^{\mathrm{FBA}}\left( t \right)\frac{1}{h}}}\\\qquad\qquad\qquad \cdot \frac{1}{{M_{\mathrm{biomass}}\frac{{g\,\mathrm{biomass}}}{{\mathrm{mmol}\,\mathrm{biomass}}}}} \cdot r_{\mathrm{biomass},{s}}(t)\frac{{\mathrm{pg}\,\mathrm{biomass}}}{{\mathrm{cell} \cdot h}},\end{array}$$

where *t* is one of the 17 time points during the cell cycle for which independent FBA simulations were implemented; *s* is one of the nine sets of coefficients in the biomass reactions obtained via the cell-mass model trained on different sets of replicate measurements; $v_{i,s}^{\mathrm{cell}}\left( t \right)$ is the flux of the reaction *i* in the absolute sense, expressed in $\mathrm {pmol}\,\mathrm{cell}^{ - 1}\mathrm{h}^{ - 1}$, without being related to a gram or mole of biomass; $v_{i,s}^{\mathrm{FBA}}\left( t \right)$ is the flux of the reaction *i* in a relative sense, expressed in $\mathrm {mmol}\,(\mathrm{mmol}\,\mathrm{biomass})^{ - 1}\,\mathrm{h}^{ - 1}$, $v_{i,s}^{\mathrm{FBA}}\left( t \right)$ was obtained in FBA; *M*_biomass_ is the molar weight of biomass in the model (0.966 *g* mmol^−1^), which was calculated by uniting the equations in Supplementary Table 7 into one and subtracting the sum of the products’ molar masses multiplied by their coefficients from the sum of the substrates’ molar masses multiplied by their coefficients; $v_{\mathrm{biomass},{s}}^{\mathrm{FBA}}\left( t \right)$ is the flux of the biomass reaction in FBA; $r_{\mathrm{biomass},{s}}(t)$ is the total biomass synthesis rate (Fig. 3c) obtained in the cell-mass model.

In Fig. 4a, to better compare the cell-cycle-phase-resolved FBA predictions (shown in Fig. 4b) with the population-level measurements in the exponentially growing culture, the calculation of the cell-cycle-average yields from the predicted fluxes was conducted by considering a long early G1 (ME to START) characteristic of newborn cells that to a large extent constitute the exponentially growing culture. Cell-cycle-average yields of extracellularly exchanged metabolites with respect to glucose (Fig. 4a; *y* axis) were calculated from the flux predictions in the following way:

$$Y_{\mathrm{met}_{EX},s}^P = \frac{{\mathop {\sum}\nolimits_t {\left[ {v_{\mathrm{met}_{EX},s}^{\mathrm{cell}}\left( t \right)\frac{\mathrm{pmol}}{{\mathrm{cell} \cdot h}} \cdot M_{\mathrm{met}}\frac{g}{\mathrm{mol}} \cdot \varphi ^P(t)} \right]} }}{{ - \mathop {\sum}\nolimits_t {\left[ {v_{\mathrm{glc}_{EX},s}^{\mathrm{cell}}\left( t \right)\frac{\mathrm{pmol}}{{\mathrm{cell} \cdot h}} \cdot M_{\mathrm{glc}}\frac{g}{\mathrm{mol}} \cdot \varphi ^P(t)} \right]} }},$$

where $Y_{\mathrm{met}_{EX},s}^P$ is the cell-cycle-average yield of the extracellularly exchanged metabolite *met*_*Ex*_ given *P*, the cell-cycle-phase distribution of cells in a population; *t* is one of the 17 time points during the cell cycle for which independent FBA simulations were implemented; *s* is one of the nine sets of coefficients in the biomass reactions obtained via the cell-mass model trained on different sets of replicate measurements; $v_{\mathrm{met}_{EX},s}^{\mathrm{cell}}\left( t \right)$ is the flux of the exchange of the metabolite *met*_*EX*_ through the plasma membrane, with positive values corresponding to excretion and negative values meaning uptake (for example oxygen uptake); $v_{\mathrm{glc}_{EX},s}^{\mathrm{cell}}\left( t \right)$ is the flux of glucose uptake (negative values); *M*_met_ and *M*_glc_ are the molar masses of the metabolite *met*_*EX*_ and glucose, respectively; $\varphi ^P(t)$ is the proportion of cells undergoing the cell-cycle phase *t* in a population given the distribution *P*. We used the following distribution *P*: $\varphi ^P\left( t \right) = \frac{{\mathrm{mean}({{\Delta }}t_{\mathrm{early} \mathrm{G}1}^{\mathrm{newborn}})}}{{{{\Delta }}t_{\mathrm{earlyG}1}^{\mathrm{mature}}}}$ for $t \in \left\{ {3,9} \right\}\left( {\min } \right)$ that are in the early G1 (between ME and START) in the mature cell, and $\varphi ^P\left( t \right) = 1$ for the rest of *t*, where $\mathrm{mean}\left( {\Delta t_{\mathrm{earlyG}1}^{\mathrm{newborn}}} \right) = 79.75\,\min$ is the mean of the early G1 duration (between ME and START) in newborn cells and $\Delta t_{\mathrm{earlyG}1}^{\mathrm{mature}} = 11\,\min$ is the early G1 duration in a mature, not newborn, cell. The duration of the rest of the cell cycle is virtually the same between the newborn and mature cells. Thus, the calculation of the cell-cycle-average yields from the predicted fluxes was performed by considering a long early G1 observed in newborn cells that to a large extent constitute an exponentially growing culture, for which measured yields are available in the same strain YSBN6 and the same medium 2% glucose YNB (Fig. 4a; *x* axis).

In Fig. 4b, the fluxes of glucose and oxygen uptake were multiplied by −1 as these fluxes are negative in the model by definition (uptake fluxes). In Fig. 4c, the turnover was calculated as the sum of ATP fluxes in reactions where this metabolite is produced: $\mathop {\sum}\nolimits_i {S_{ij}v_i^{\mathrm{cell}}(t)}$, if $S_{ij}v_i^{\mathrm{cell}}\left( t \right) > 0$, where *S*_*ij*_ is the coefficient of *j* = ATP in the reaction *i* that has the flux $v_i^{\mathrm{cell}}\left( t \right)$ (mol cell^−1^ h^−1^) (due to the steady-state assumption of FBA, the turnover values would be the same if ATP-depletion fluxes only were summed). We showed individual reactions *i* whose cytoplasmic flux of *j* = ATP calculated as $S_{ij}\,v_i^{\mathrm{cell}}\left( t \right)$ is bigger than 0.09 or smaller than −0.09 (mol cell^−1^ h^−1^) in at least one cell-cycle phase. In Fig. 4d, for each precursor, we calculated the sum of its fluxes, $\mathop {\sum}\nolimits_i {S_{ij}v_i^{\mathrm{cell}}(t)}$, in the reactions diverting from central metabolic pathways to the synthesis of major biomass components: for ribose 5-phosphate (r5p), its fluxes in the syntheses of AMP, GMP and UMP as well as phosphoribosyl pyrophosphate; for erythrose 4-phosphate (e4p), its flux in the synthesis of 3-deoxy-d-arabino-heptulosonate 7-phosphate; for phosphoenolpyruvate (pep), its fluxes in the reactions of 3-deoxy-d-arabino-heptulosonate 7-phosphate synthetase and 3-phosphoshikimate 1-carboxyvinyltransferase; for pyruvate (pyr), the l-alanine flux in the reaction of protein biosynthesis; for acetyl-CoA (accoa), its fluxes in the reactions of zymosterol synthesis, homoserine *O*-trans-acetylase, 2-isopropylmalate synthase and lipid biosynthesis; for glycerol 3-phosphate (g3p), its flux in lipid biosynthesis; for glucose 6-phosphate (g6p), its fluxes in polysaccharide and lipid biosynthesis; for fructose 6-phosphate (f6p), its flux in polysaccharide biosynthesis.

### Estimating the glucose-uptake flux during the cell cycle

The experiment with 2-NBDG addition was performed in two replicates. The strain YSBN6 WHI5-mCherry was recovered from a −80 °C stock on a 2% (m/V) glucose YPD plate, whose single colony initiated an overnight pre-culture in 1% (m/V) glucose YNB medium. Afterward cells were cultivated in 0.015% (m/V) glucose YNB medium for several hours before being loaded into the microfluidic device. With microscopy imaging every δt = 6 min, we monitored cells inside the microfluidic device in the following channels: BF (3 V, 50 ms), GFP (2%, 200 ms) and RFP (10%, 600 ms), via microscope setup 1b and ×100 objective. In the microfluidic device, we cultivated cells for 4.8–5.2 h in the YNB medium with 0.015% (m/V) glucose and 0.6% (V/V) dimethylsulfoxide (DMSO) (the vehicle of the subsequently added 2-NBDG). Due to the competition between glucose and 2-NBDG for hexose transporters^88^, cells were cultivated with a lower glucose concentration (0.015%), which, nevertheless, led to the same average cell-cycle duration of ~100 min as on 2% glucose used in other experiments. We used the air-pressurized pumping system together with the flow sensor to maintain the flow rate of 5 µl min^−1^. Afterward, we paused the time-lapse microscopy and, at the beginning of this interruption period, stopped the flow of the medium for several minutes to switch it manually (Switch 1), by cutting and reconnecting the tubing in the ~10 cm proximity of the microfluidic device. Specifically, we switched to the YNB medium with 0.015% (m/V) glucose, 0.6% (V/V) DMSO and 180 µM 2-NBDG (Thermo Fisher, N13195) (DMSO was used to dissolved 2-NBDG in its 10 mg ml^−1^ stock); the flow rate was returned to 5 µl min^−1^. After two consecutive rounds of imaging of all *XY* positions in the microfluidic chamber, we paused the microscopy again and, at the beginning of this second interruption period, stopped the medium flow for several minutes to manually switch back to the previous medium lacking 2-NBDG (Switch 2). The flow rate was set to 5 µl min^−1^ again.

The pulse of 2-NBDG in the cellular environment was estimated to last ~13–15-min. The middle of this time period was then used to measure the cell-cycle phases when individual cells experienced the pulse of 2-NBDG, which was conducted in a manner analogous to the analysis of the stop-and-respond experiments (such as using the latest cell-cycle event before the perturbation and excluding cells with abnormally long cell-cycle phases; for more details see above).

To tackle uneven illumination in the GFP channel (manifesting in the brighter center and darker corners of an image), which may confound low-signal intracellular 2-NBDG fluorescence, we implemented a flat-field correction. Specifically, we stacked GFP-channel images from almost all *XY* positions at the time point before Switch 1 and calculated the median intensity for each pixel. The resulting image with median intensities lacked the structure with microfluidic device pads and cells that were visible in the images of individual *XY* positions. We subtracted the camera baseline value of 500 from the image with median intensities and fitted a two-dimensional (2D) Gaussian distribution to the image to learn the shape of the uneven illumination. To correct for the uneven illumination, we subtracted from every GFP-channel image of the movie the camera baseline value and multiplied the image by the ratio between the maximal value of the fitted 2D Gaussian and the 2D Gaussian itself.

To measure the fluorescence of the intracellular (acquired) 2-NBDG, we used the first microscopy image after Switch 2, when 2-NBDG was gone from the extracellular environment. We did not use images between Switch 1 and 2, with the medium containing 2-NBDG, because the high extracellular fluorescence under and above a segmented cell likely confounded the intracellular fluorescence in the wide-field microscopy (which was supported by large values of fluorescence within the cell’s segmentation and gradual decrease in pixel intensities when moving from the cell’s edges to the center).

To remove the contribution of cellular autofluorescence in the GFP channel to the measurement of the intracellular 2-NBDG fluorescence, we subtracted from a cell’s fluorescence after Switch 2 the mean value of the autofluorescence in the five time points before Switch 1 (we did not observed a cell-cycle dependency of cellular autofluorescence, therefore, correction for it was not cell-cycle-related, rather it was individual cell-related). By implementing this correction, we also removed the contribution of the background to the measured fluorescence within a cell’s segmentation (rolling-ball background correction was not implemented for these experiments).

We noticed that the intracellular fluorescence of the accumulated 2-NBDG decreased as a function of the time that a cell was kept in the medium without the glucose analog (likely due to reverse transport of the analog to the environment). The first microscopy imaging after Switch 2 that we used to measure the accumulated 2-NBDG was performed at slightly different time moments in different *XY* positions, therefore, cells in these positions were kept in the analog-free medium for slightly different time periods. We found that cells in the positions that were imaged later had lower values of the intracellular fluorescence. To correct for it, we united the cells from up to four *XY* positions imaged immediately after each other, calculated the median fluorescence of the accumulated 2-NDBG in these cells and normalized by it the individual cell values. After this normalization, we merged the cells from all *XY* positions, assigned to them the cell-cycle phases in which they experienced the pulse and run a Gaussian process regression to find a cell-cycle dependency in the intracellular 2-NBDG fluorescence. In the regression, we used as a prior an RBF kernel and a white kernel, maximizing the log-marginal likelihood.

### Estimating glycolytic flux during the cell cycle

Two strains, namely YSBN10 glycolytic biosensor and YSBN10 control for glycolytic biosensor, were each grown in two consecutive exponential 2% (m/V) glucose YNB cultures. The strains were loaded in two separate microfluidic devices attached to the same cover glass. In both microfluidic devices, cells were provided with 2% (m/V) glucose YNB medium at the flow rate of 4 μl min^−1^ via a syringe pump. With microscopy imaging every δt = 6 min, we simultaneously monitored cells inside both microfluidic devices in the following channels: BF (3 V, 50 ms), YFP (50%, 300 ms, EM gain 25) and RFP (25%, 200 ms, EM gain 25), via microscope setup 2a and ×100 objective. The analysis of microscopy data and the derivation of the uncoupling between YFP and mCherry production rates during the cell cycle is described elsewhere^47^, where this biosensor was used to assess glycolytic flux during the cell cycle in a respiratory metabolic condition (TM6* strain growing on 2% glucose). The length‐scale range of the RBF kernel that was used in the Gaussian process regression for smoothing the volume, YFP and mCherry fluorescence trajectories was set to [18, 48] {min}. The maturation half‐times were assumed to be 25 min for YFP and 50 min for mCherry.

### Dynamic switches between aerobic and microaerobic conditions

A 1% glucose modified Verduyn minimal medium was used to cultivate the cells of the strain YSBN6 Atp3-mCherry (Supplementary Table 1; our experiments did not focus on properties originating from the cassette pTEF1-pH-tdGFP-pADH1-OsTIR1-KanMX4 in the HO locus). To maintain the aerobic condition in the microfluidic device with cultivated cells, we continuously provided the medium that, right before the experiment, had been aerated by shaking for several hours in a 100-ml Erlenmeyer flask. To make the cells’ environment microaerobic, we provided the medium that had been bubbled with nitrogen for 1 h immediately before the experiment. To minimize the exposure of this medium to atmospheric oxygen, we did not change its reservoirs before the experiment and bubbled nitrogen in the syringe that was later used to inject the medium in the microfluidic chamber. The syringe pump was employed to maintain the medium flow rate at 3.6–4 µl min^−1^. To switch between the aerobic and microaerobic conditions, in a close proximity to the microfluidic device, we cut and reconnected the tubing coming from two syringes that contained the aerated and nitrogen-bubbled medium, respectively.

To decrease the contact of the nitrogen-bubbled medium with atmospheric oxygen through the tubing or the material of the microfluidic device (PDMS), we added a range of accessories to the microfluidic setup. First, we connected the syringe with this medium to the air-impermeable tubing (VICI Jour, JR-T-6130-M3) that, in ~10 cm proximity to the microfluidic device, was attached to the Tygon microbore tubing (0.030 inch inner diameter × 0.090 inch outer diameter) followed by the PTFE microbore tubing (0.012 inch inner diameter × 0.030 inch outer diameter) wrapped in parafilm and epoxy glue. A short fragment of the Tygon and PTFE microbore tubing needed for the medium switch in the closest vicinity to the microfluidic device was not protected by the parafilm and epoxy glue. As the second modification to the microfluidic setup previously described^14,15^, we used a transparent plastic plate of ~5-mm thickness to close the top of the metal holder that accommodated the cover slip at the bottom and, on it, the PDMS chip both forming the microfluidic device. With the help of screws, the cover slip and the plastic plate were tightly connected to the metal holder, with a grease applied at interfaces to block contacts with the outside air. The plastic plate contained three small holes with the diameter slightly bigger than 0.030 inch through which PTFE microbore tubing was inserted to connect with the PDMS chip’s inlet, side channel and outlet, respectively. The plastic plate also contained two bigger holes with 0.090-inch diameter and Tygon microbore tubing providing nitrogen (when necessary) was tightly connected to one of these. Therefore, the PDMS chip with the microfluidic chamber with trapped cells was concealed in a small box formed by the metal holder, the cover slip and the plastic plate. When the aerated medium was switched to the nitrogen-bubbled medium, this box was continuously ventilated with nitrogen, preventing the increase of oxygen level in the cells’ environment due to the air permeability of PDMS.

Microscopy details were setup 2a; ×40 objective; BF (3 V, 50 ms), NAD(P)H (15%, 200 ms), GFP (2%, 30 ms), RFP (25%, 250 ms); time step *δt* *=* 5 min. For Fig. 5a and Extended Data Fig. 9, mCherry fluorescence was determined as the average value across the pixels of the entire mother cell. In Fig. 5a, one unit of mCherry fluorescence was the minimal value of the smoothed trajectory of this cell.

### NAD(P)H dynamics in the carbohydrate-storage mutant

A 1% glucose modified Verduyn minimal medium was used to cultivate the cells of the strain YSBN6 ΔTps1ΔTps2ΔGsy1ΔGsy2 (Supplementary Table 1). Microscopy details were setup 2a; ×100 objective; BF (3 V, 200 ms), NAD(P)H (20%, 200 ms); time step *δt* = 5 min. The syringe pump was employed to maintain the medium flow of 4.8 µl min^−1^. In Fig. 5b, not every metabolic oscillation was accompanied by budding, which was described earlier^44^.

### Cell-cycle NAD(P)H dynamics in different growth conditions

Experiments were performed with the strain YSBN6.G2J (Supplementary Table 1; our experiments did not focus on properties originating from the cassette KanMX4-pTEF1-mGFP-AID-tCYC-pADH1-AtTIR-tADH1 in the HO locus). Cells were cultivated either in modified Verduyn’s minimal medium (MM) or in YPD. In case of conditions containing 1% glucose, cells were cultivated in two consecutive exponentially growing batch cultures and then loaded to the microfluidic device. To get cells growing on 2% pyruvate MM in the microfluidic device, we first inoculated the strain in a flask with 1% glucose MM for a 1-d cultivation to pass the diauxic shift, then diluted the culture at the OD 0.1 in a flask with 2% pyruvate MM for an overnight growth and, again, diluted the culture at the OD ~0.05 in the same medium so that the cells grew exponentially for 1 d before loading. We cultivated cells with the same medium in two last consecutive exponentially growing batch cultures and afterward in the microfluidic device, aside from the case of 1% Glu + CSM (Formedium, DCS0019) to which cells were shifted after ~7 h of growing on 1% Glu in the microfluidic device. Minimizing the probability that the adaptation to this shift confounds the cell-cycle-related NAD(P)H dynamics, for this analysis, we processed the NAD(P)H data after 4 h of growing on 1% Glu + CSM.

The syringe pump was employed to maintain the medium flow in the microfluidic device. The composition of the medium as well as the details of microscopy and microfluidic cultivation are given in Supplementary Table 8. We detrended single-cell NAD(P)H traces (average pixel intensity in the mother-cell compartment), dividing them by the corresponding curves obtained using LOWESS with large window sizes for line fitting (see window size values in Supplementary Table 8). Before detecting the phase of NAD(P)H oscillation’s crest and trough, we smoothed the detrended NAD(P)H traces using LOWESS with small window sizes for line fitting (Supplementary Table 8). For Fig. 5d and Extended Data Fig. 10a, to calculate the phase, cell-cycle-relative time, each time point of *t* minutes between two adjacent budding events happening at $t_{\mathrm{BUD}}^{\,i}$ and $t_{\mathrm{BUD}}^{\,i + 1}$ minutes is converted in the following way: $\frac{{t - t_{\mathrm{BUD}}^{\,i}}}{{t_{\mathrm{BUD}}^{\,i + 1} - t_{\mathrm{BUD}}^{\,i}}}$. In Fig. 5d, the values of the detrended NAD(P)H fluorescence are linearly interpolated at 100 phase points, from 0 to 1, for which we calculated the median NAD(P)H fluorescence and obtained its confidence interval via bootstrapping with 5,000 iterations. For Extended Data Fig. 10a, the frequency was determined as the inverse time difference between two adjacent buddings: $\frac{1}{{t_{\mathrm{BUD}}^{\,i + 1} - t_{\mathrm{BUD}}^{\,i}}}$. The phase of the crest is identified as the phase in the range (−0.25, 0.5) at which the detrended and smoothed NAD(P)H fluorescence is maximal (negative phase values correspond to the time preceding $t_{\mathrm{BUD}}^{\,i}$, the first of the two analyzed adjacent buddings). The phase of the trough is identified as the phase in the range (0.25, 1) at which the detrended and smoothed NAD(P)H fluorescence is minimal. Cells cultivated in 1% Glu (2) were later shifted to 1% Glu + CSM. The marked variability between the cells in 1% Glu and 1% Glu (2) is likely explained by the difference in light exposure (Supplementary Table 8) and, hence, by different degrees of phototoxicity during the corresponding microscopy experiments. For Extended Data Fig. 10b, we calculated budding frequency as $\frac{1}{{t_{\mathrm{BUD}}^{\,i + 1} - t_{\mathrm{BUD}}^{\,i}}}$. To calculate the respective NAD(P)H oscillation peak frequency, we identified the oscillation peaks in detrended and smoothed single-cell NAD(P)H trajectories through visual inspection assisted by an automatic local maximum detection tool (Scipy’s signal.find_peaks). For each budding event, we then found the closest NAD(P)H oscillation peak $t_{\mathrm{peak}}(t_{\mathrm{BUD}}^{\,i})$, which allowed us to calculate the NAD(P)H oscillation peak frequency as $\frac{{N\left( {i,i + 1} \right) - 1}}{{t_{\mathrm{peak}}(t_{\mathrm{BUD}}^{\,i + 1}) - t_{\mathrm{peak}}(t_{\mathrm{BUD}}^{\,i})}}$, where *N*(*i*,*i* + 1) is the number of the NAD(P)H oscillation peaks in the inclusive interval $\left[ {t_{\mathrm{peak}}\left( {t_{\mathrm{BUD}}^{\,i}} \right),t_{\mathrm{peak}}\left( {t_{\mathrm{BUD}}^{\,i + 1}} \right)} \right]$, which is in the majority of cases equal to 2.

### Reporting summary

Further information on research design is available in the Nature Portfolio Reporting Summary linked to this article.

## Supplementary information

Supplementary Information Supplementary Methods and Supplementary Tables 1–8.

Reporting summary

## Data Availability

Data extracted from microscopy imaging, analysis- and modeling-related data required to generate the figures are available at dataverse.nl (10.34894/XPYC7Y). Microscopy raw data can be obtained from M.H. Strains can be obtained from Addgene and M.H.

## Code availability

Code underlying the models (the cell-mass model and the FBA model) and analysis files (Python) required to generate the figures are available at dataverse.nl (10.34894/XPYC7Y).
