# Supplementary material for: Temporal segregation of biosynthetic processes is responsible for metabolic oscillations during the budding yeast cell cycle
Source: Nat Metab. 2023 Feb 27;5(2):294–313. doi: 10.1038/s42255-023-00741-x (PMC9970877; doi:10.1038/s42255-023-00741-x)
Supplement: Supplementary file 1 — Supplementary Methods and Supplementary Tables 1–8. [file 42255_2023_741_MOESM1_ESM.pdf]

nature metabolism

Article https://doi.org/10.1038/s42255-023-00741-x

Temporal segregation of biosynthetic processes is responsible for metabolic oscillations during the budding yeast cell cycle

In the format provided by the authors and unedited

CONTENTS

**Supplementary Methods**	The mathematical model of cell mass during the cell cycle that converts the measured dimensionless biosynthetic activities into rates expressed in absolute units
**Supplementary Table 1**	Yeast strains used in this study
**Supplementary Table 2**	Primers employed in the construction of recombinant strains
**Supplementary Table 3**	Conditions and strains used in the stop-and-respond experiments
**Supplementary Table 4**	The time points of metabolic perturbation used to collect the single cells' NAD(P)H derivative values for the analysis of the cell-cycle-dependent response in the stop-and-respond experiments
**Supplementary Table 5**	Model selection: oscillatory models explain the cell-cycle-resolved experimental data better and have higher predictive performance compared to linear models
**Supplementary Table 6**	Parameter values used in the mathematical model describing cell mass during the cell cycle and converting the measured dimensionless biosynthetic activities into the rates expressed in absolute units
**Supplementary Table 7**	New equations introduced into the thermodynamic-stoichiometric model instead of the original biomass equation
**Supplementary Table 8**	Growth conditions among which cell-cycle-associated NAD(P)H oscillations were compared, and respective microscopy and analysis settings

Supplementary Methods

The mathematical model of cell mass during the cell cycle that converts the measured dimensionless biosynthetic activities into rates expressed in absolute units

To convert the activities of protein, lipid and polysaccharide biosynthesis, obtained with the stop-and-respond method and expressed in arbitrary units, into the biosynthetic rates expressed in *pg/min* as well as to estimate these biosynthetic rates for RNA and DNA, we formulated a mathematical model describing the dynamics of cell mass and its composition during the cell cycle. This model represents an optimisation problem that we solved employing General Algebraic Modeling System (GAMS) (39.3.0) with the global solver ANTIGONE Version 1.1 [85]. The code implementing the model is attached to this paper.

1. *Cell cycle phases*

$$t \in T = \{T_i\}_{i=1}^{n}, \delta t = T_{i+1} - T_i$$

Parameters:

$t\ (min)$ – the cell cycle phase regarding ME.

$T_1 = 3\ min$ – the first cell cycle phase regarding mitotic exit (ME) at which the biosynthetic activities were measured with the stop-and-respond method. We assume that cytokinesis happens at this cell cycle phase.

$T_n = T_{cell\ cycle} = 99\ min$ – the phase of the next ME, the cell cycle duration.

$\delta t = 6\ min$ – the time step (used in microscopy imaging during dynamic perturbation experiments).

2. *Cell volume*

$$V(t) = V^m(t) + V^{err}, V(t) > 0$$

$$-\sigma_V^{max} \le V^{err} \le \sigma_V^{max}$$

Variables:

$V(t)\ (fL)$ – the estimated cell volume during the cell cycle.

$V^{err}\ (fL)$ – the error for the volume estimation.

Parameters:

$V^m(t)$ (fL) – the posterior mean (Gaussian process regression with a radial basis function, RBF, kernel) of the cell volume obtained in single-cell measurements (Extended Data Fig. 4, solid curve). Although, when summarising the cell-volume dynamics across multiple cell-cycle traces, we related the phase to four cell-cycle events making it dimensionless (from 0 to 1), here we replaced this dimensionless phase by the phase measured in minutes $t \in T$, motivated by the fact that the average cell-cycle duration corresponding to the summarised cell-volume traces is similar to $T_{cell\ cycle}$. Specifically, we used for this optimisation problem the part of the curve of the posterior mean between and including the phases 0.03 and 1, and assigned the new phases $t \in T$ used here.

σ_V^{max} (fL) – the maximal posterior standard deviation (Gaussian process regression with RBF kernel) of the cell volume during the cell cycle (Extended Data Fig. 4, half of the maximal width of the shaded area between the phases 0.03 and 1).

3. *Empirical cell mass*

$$m^{emp}(t) = V(t) \cdot D_{literature}(t), m^{emp}(t) > 0$$

Variables:

$m^{emp}(t)$ (pg) – empirical cell-cycle-phase-dependent wet mass of the cell, *i.e.* including water.

Parameters:

$D_{literature}(t)$ (pg/fL) – the cell-cycle-phase-dependent cell density derived from literature [33]. In this source, the cell-cycle phase was measured from cytokinesis to cytokinesis and related to the cell-cycle duration, becoming dimensionless (from 0 to 1). Since the density was measured at discrete phases, we fit a cubic spline going through the measurements, obtaining a continuous curve. We aligned this literature-derived cell-cycle-phase-dependent curve of the cell density to the cell-cycle timing used here $t \in T$, assuming that the phase of ME regarding cytokinesis is 0.(96) (96 min/99 min) and replacing the dimensionless phases by the phases measured in minutes $t \in T$.

4. *Cell-mass estimate*

$$j \in \{proteins, lipids, polysaccharides, DNA, RNA\}$$

$$m_{dry}^{est}(t) = \sum_j m_j(t) + m_r \cdot V(t), m_{dry}^{est}(t) > 0$$

$$m^{est}(t) = \sum_j b_j\, m_j(t) + b_r \cdot V(t), m^{est}(t) > 0$$

$$b_j > 1, b_j \geq b_j^{lower}$$

$$b_r > m_r > 0$$

$$\frac{\sum_t m_{dry}^{est}(t) \cdot \varphi^P(t)}{\sum_t m^{est}(t) \cdot \varphi^P(t)} = \omega$$

$$\omega^{lower} \leq \omega \leq \omega^{upper}$$

Variables:

$m_{dry}^{est}(t)$ (pg) – the cell-cycle-phase-dependent dry mass of the cell.

$m_j(t)$ (pg) – the mass of the major biomass component j at the phase t.

m_r (pg/fL) – the mass of remaining dry material per cell-volume unit *(e.g.* metabolites, metal ions whose mass, as a whole, is assumed to scale with the cell volume).

$m^{est}(t)$ (pg) – the estimate of the wet mass (to approximate $m^{emp}(t)$ in the optimisation).

b_j – the mass of the component j together with the accompanying water divided by the mass of the dry component j. By definition, b_j is bigger than 1.

b_r (pg/fL) – the mass of the remaining material with the accompanying water per cell-volume unit.

ω – the fraction of the dry mass regarding the wet mass in the population of asynchronous cells with the uniform distribution across the cell cycle (which is assumed to correspond to the fraction of the dry mass regarding the wet mass of the cell reported in population-level studies).

Parameters:

b_j^{lower}– the lower boundary for b_j (Supplementary Table 6).

$\omega^{lower}, \omega^{upper}$ – the lower and upper boundaries for ω (Supplementary Table 6).

$\varphi^P(t)$ – the proportion of cells undergoing the cell-cycle phase t in a population given the distribution P. We used the following distribution P: $\varphi^P(t) = \frac{mean(\Delta t_{earlyG1}^{newborn})}{\Delta t_{earlyG1}^{mature}}$ for $t \in \{3,9\}$ (min) that are in the early G1 (between ME and START) in the mature cell, and $\varphi^P(t) = 1$ for the rest of t, where $mean(\Delta t_{earlyG1}^{newborn}) = 79.75$ min is the mean of the early G1 duration (between ME and START) in newborn cells and $\Delta t_{earlyG1}^{mature} = 11$ min is the early G1 duration in a mature, *i.e.* not newborn, cell. The duration of the rest of the cell cycle is virtually the same between the newborn and mature cells. Thus, the relation between the cell-cycle-average water content of a mature cell in the model and population-level measurements was established by considering a long early G1 (ME to START) characteristic of newborn cells that to a large extent constitute an exponentially growing culture, for which the water content values are available.

5. *Measured dimensionless biosynthetic activities*

$$i \in \{proteins, lipids, polysaccharides\}$$

$$f_i(t) = f_i^m(t) + f_i^{err}, f_i(t) > 0$$

$$-\sigma_{f_i^m}^{max} \le f_i^{err} \le \sigma_{f_i^m}^{max}$$

Variables:

$f_i(t)$ $(a.u._i \sim assumed \frac{pg}{min})$ – the biosynthetic activity estimate.

f_i^{err} $(a.u._i \sim assumed \frac{pg}{min})$ – the error for the biosynthetic activity estimation.

Parameters:

$f_i^m(t)$ $(a.u._i \sim assumed \frac{pg}{min})$ – the measured dimensionless biosynthetic activity, which is the posterior mean (Gaussian process regression with RBF kernel) of the single-cell NAD(P)H response to its inhibition (Fig. 1d, 2a,c). The model was simulated using different combinations of the replicate measurements of the biosynthetic activities (See more details in the caption of Fig. 3).

$\sigma_{f_i^m}^{max}$ $(a.u._i \sim assumed \frac{pg}{min})$ – the maximal posterior standard deviation (Gaussian process regression with RBF kernel) of the single-cell NAD(P)H response to its inhibition (Fig. 1d, 2a,c, half of the maximal width of the shaded area between the phases of 3 and 99 min).

6. *Biosynthetic rates expressed in absolute units*

$$i \in \{proteins, lipids, polysaccharides\}:$$

$$F_i(t) = k_i f_i(t), F_i(t) > 0.$$

We assume that DNA biosynthesis has constant rate between budding and karyokinesis:

$$F_{DNA}(t) = \begin{cases} 0, t \in [T_1, t_{BUD}) \cup (t_{kk}, T_n] \\ r_{DNA}, t \in [t_{BUD}, t_{kk}] \end{cases}, r_{DNA} > 0.$$

To model the dynamics of RNA biosynthesis, we consider separately ribosomal RNA (rRNA), accounting for 80% of total RNA [90], and other forms of RNA (oRNA). We assume that the rate of rRNA biosynthesis is proportional to the rate of protein biosynthesis during its first wave (from kariokynesis through the whole G1 until budding) being constant and lower in the rest of the cell cycle. This assumption is based on reports that the transcription of genes responsible for rRNA processing and ribosome biogenesis peaks once during the cell cycle, specifically in G1 [11,34]. We assume that the biosynthetic rate for less abundant oRNA is constant.

$$F_{RNA}(t) = F_{rRNA}(t) + F_{oRNA}(t), F_{RNA}(t) > 0$$

$$F_{rRNA}(t) = \begin{cases} k_{rRNA} f_{proteins}(t), t \in [T_1, t_{BUD}) \cup (t_{kk}, T_n] \\ k_{rRNA} f_{proteins}(t_{BUD} - \delta t), t \in [t_{BUD}, t_{kk}] \end{cases},$$

$$F_{oRNA}(t) = k_{oRNA}, k_{oRNA} > 0.$$

$$j \in \{proteins, lipids, polysaccharides, DNA, rRNA, oRNA\}:$$

$$F_j^s(t) = \begin{cases} F_j(T_1)\frac{\delta t}{2}, t = T_1 \\ \sum_{\tau = T_1 + \delta t}^{t} \frac{F_j(\tau) + F_j(\tau - \delta t)}{2}\delta t + F_j(T_1)\frac{\delta t}{2}, t \in [T_1 + \delta t, T_n] \end{cases}$$

$$m_j(t) = F_j^s(t) + m_j^0.$$

Linking the masses of RNA, rRNA and oRNA:

$$F_{RNA}^s(t) = F_{rRNA}^s(t) + F_{oRNA}^s(t),$$

$$m_{RNA}(t) = m_{rRNA}(t) + m_{oRNA}(t),$$

$$\frac{\sum_t m_{rRNA}(t)}{\sum_t m_{RNA}(t)} = \xi_{rRNA}.$$

Utilising the literature-derived mass fractions of macromolecules:

$$l \in \{proteins, lipids, polysaccharides, DNA, RNA\},$$

$$\frac{\sum_t m_l(t) \cdot \varphi^P(t)}{\sum_t m_{dry}^{comp}(t) \cdot \varphi^P(t)} = \xi_l,$$

$$\sum_l \xi_l < 1,$$

$$0.995\xi_l^m \le \xi_l \le 1.005\xi_l^m.$$

Variables:

$F_i(t)$ (pg/min) – the biosynthetic rate for the biomass component i, such as proteins, lipids, polysaccharides, RNA, rRNA (ribosomal RNA), oRNA (RNA excluding rRNA) or DNA. RNA is the union of rRNA and oRNA. This rate is expressed in absolute units.

k_i $(\frac{pg}{a.u._i \cdot min})$ – the proportionality constant used in the conversion of the activity of the biosynthesis of the component i into the biosynthetic rate.

r_{DNA} (pg/min) – DNA replication rate (assumed to be constant between budding and karyokinesis).

k_{rRNA} – the proportionality factor between the rates of rRNA and protein biosyntheses during the G1-wave of the latter.

k_{oRNA} $(\frac{pg}{min})$ – the assumed constant rate of oRNA biosynthesis.

$F_j^s(t)$ (pg) – the mass of the component j synthesized from the phase 0, that is mitotic exit, to t.

m_j^0 (pg) – the mass of the component j at the phase 0 in the mother cell, *i.e.* the mass inherited from the preceding cell cycle.

$m_j(t)$ (pg) – the mass of the component j at the phase t.

ξ_l and ξ_{ash} – the fractions of the component l's mass and the ash mass (not proteins, lipids, polysaccharides, DNA and RNA) regarding the dry mass in the population of asynchronous cells with the uniform distribution across the cell cycle (which is assumed to correspond to the reported fractions of biomass components in the dry mass of the cell).

Parameters:

$t_{BUD} = 33\ min$, $t_{kk} = 81\ min$ – the phases of budding and karyokinesis between which DNA replication is assumed to happen. This phase for budding is chosen among $T = \{T_i\}_{i=1}^n$ as the closest to the mean phase of budding observed in multiple experiments (Fig. 1d, 2a,c). DNA replication is assumed to begin at budding in line with [6,18,19]. Karyokinesis is identified as the rapid drop in the amount of the histone protein Hta2 in the mother cell (Extended Data Fig. 2).

$\xi_{rRNA} = 0.8$ – the mass fraction of rRNA relative to total RNA in the population of asynchronous cells with the uniform distribution across the cell cycle (which is assumed to correspond to the reported fraction) [90]

ξ_l^m – the literature-derived values for ξ_l (Supplementary Table 6),

$\varphi^P(t)$ – the proportion of cells undergoing the cell-cycle phase t in a population given the distribution P. See more details in the formulation of the water content.

7. *Biomass inherited from the preceding cell cycle in the mother cell*

$$j \in \{proteins, lipids, polysaccharides, DNA, RNA, rRNA, oRNA\}$$

$$x_j = x_j^m + x_j^{err}$$

$$-\sigma_{x_j^m} \le x_j^{err} \le \sigma_{x_j^m}$$

$$x_{proteins} = x_{RNA} > x_{lipids} = x_{polysaccharides}{}^*$$

$$x_j \cdot m_j(T_n) = m_j^0$$

Variables:

x_j – the estimated fraction of the component *j*'s mass present in the mother cell at mitotic exit.

x_j^{err} – the error for the estimation of this fraction.

Parameters:

x_j^m, $\sigma_{x_j^m}$ – the average and standard deviation of the measured fraction of the component *j*'s mass present in the mother cell at mitotic exit. This fraction is calculated on the basis of the cell volume for proteins and RNA, and on the basis of the cell surface area for lipids and polysaccharides (Supplementary Table 6).

* – in individual cell-cycle traces, it was observed that the fraction calculated on the basis of the cell volume is bigger than the fraction calculated on the basis of the cell surface area.

The x_j can be strictly applied to the phases of mitotic exit. Since T_1 follows mitotic exit, the cell volume is allowed to be slightly higher:

$$x_{proteins} \cdot V(T_n) < V(T_1)$$

$$1.05 \cdot x_{proteins} \cdot V(T_n) > V(T_1)$$

8. *Constraint for protein concentration dynamics*

According to the dynamics of the signals of fluorescent proteins, protein concentration peaks once during the cell cycle, increasing in G1 and decreasing in the second half of S/G2/M:

$$c_{proteins}(t) = \frac{m_{proteins}(t)}{V(t)},$$
$$c_{proteins}(t) - c_{proteins}(t - \delta t) > 0.001, t \in [9,33],$$
$$c_{proteins}(t) - c_{proteins}(t - \delta t) < -0.001, t \in [69,93].$$

Variables:

$c_{proteins}(t)$ $(\frac{pg}{fl})$ – protein concentration.

9. *Objective function*

$$minimize \sum_{t=T_1}^{T_n} (m^{emp}(t) - m^{est}(t))^2$$

This model was simulated nine times: 1) using eight sets of replicate measurements of protein, lipid and polysaccharide biosynthesis $s(Rp, Rl, Rps) = \{f^m_{proteins,Rp}(t),\ f^m_{lipids,Rl}(t),\ f^m_{PS,Rps}(t), \sigma^{max}_{f^m_{proteins,Rp}}, \sigma^{max}_{f^m_{lipids,Rl}}, \sigma^{max}_{f^m_{PS,Rps}}\}$, with Rp, Rl and Rps being one of two replicate measurements for each biosynthesis (Fig. 3a, left panel); 2) using one input data set where two replicate measurements of each macromolecule biosynthesis were averaged: $s(average) = \left\{\{f^m_{i,average}(t), \sigma^{max}_{f^m_{i,average}}\}\ i \in \{proteins, lipids, PS\}\right\}$, where $f^m_{i,average}(t) = 0.5\left[f^m_{i,R1i}(t) + f^m_{i,R2i}(t)\right]$ and $\sigma^{max}_{f^m_{i,average}} = \max_t 0.5\sqrt{[\sigma_{f^m_{i,R1i}}(t)]^2 + [\sigma_{f^m_{i,R2i}}(t)]^2}$, with $R1i$ and $R2i$ being the first and second replicate measurements of the activity of i biosynthesis. Therefore, we obtained nine respective sets of cell-cycle-resolved momentary relative contributions of major biosynthetic processes to the total biomass production (Fig. 3d) and nine respective sets of biomass production rates (Fig. 3c). Accordingly, we derived nine sets of cell-cycle-resolved biomass-component coefficients for the biomass reaction in the metabolic model and simulated flux balance analysis nine times per cell-cycle time point.

Supplementary Table 1. Yeast strains used in this study

Strain	Genotype	Source
YSBN6	YSBN6 *wild type*:<br>S288C-derived strain, MATa FY3 HO::HphMX4	[74],<br>from Steve Oliver lab,<br>Cambridge
YSBN6 Atp3-mCherry	YSBN6 *ATP3::mCherry-AID$^{71-114}$-NatMX HO::pTEF1-pH-tdGFP-pADH1-OsTIR1-KanMX4*	This study: transformation of YSBN6.pH-tdGFP.OsTir1.KanMX4 with linearized GA46
YSBN6.pH-tdGFP.OsTir1.KanMX4	YSBN6 *HO::pTEF1-pH-tdGFP-pADH1-OsTIR1-KanMX4*	This study: transformation of YSBN6 with linearized GA38
YSBN6-Cas9	YSBN6 *Can1Δ::cas9-natNT2*	This study: transformation of YSBN6 with the cassette from IMX585 [76]
YSBN6 ΔTps1ΔGsy2	YSBN6 *ΔTps1ΔGsy2 Can1Δ::cas9-natNT2*	This study: double gene deletion in YSBN6-Cas9 via the CRISPR/Cas9 system assisted by pROS_phleo-Tps1/Gsy2 plasmid
YSBN6 ΔTps1ΔTps2ΔGsy1ΔGsy2	YSBN6 *ΔTps1ΔTps2ΔGsy1ΔGsy2 Can1Δ::cas9-natNT2*	This study: double gene deletion in YSBN6 ΔTps1ΔGsy2 via the CRISPR/Cas9 system assisted by pROS13-Tps2/Gsy1 plasmid
YSBN6.G2J	YSBN6 *HO::KanMX4-pTEF1-mGFP-AID-tCYC-pADH1-AtTIR-tADH1*	[44], Addgene plasmid #102882
YSBN6.tetO7-sfGFP	YSBN6 *HO::tetO7-sfGFP-KanMX WHI5::mCherry-BLE*	This study: transformation of the strain YSBN6 WHI5::mCherry-BLE [8] with linearized pB
YSBN6.pTEF1-sfGFP	YSBN6 *HO::pTEF1-sfGFP-KanMX WHI5::mCherry-BLE*	[8]
YSBN6 Ugp1-mCherry-AID	YSBN6 *UGP1::mCherry-AID$^{71-114}$-NatMX WHI5::mGFP-ZEO HO::ADH1p-OsTIR1-KanMX4*	This study: transformation of YSBN6.OsTIR1w/oGFP [44] (Addgene plasmid #102883) with linearized pUGP1.1 and Whi5-mGFP-ZEO cassette
YSBN6.AIDcontrol	YSBN6 *WHI5::mGFP-ZEO HO::ADH1p-OsTIR1-KanMX4*	This study: transformation of YSBN6.OsTIR1w/oGFP [44] (Addgene plasmid #102883) with Whi5-mGFP-ZEO cassette
YSBN6 Hta2-mRFP1	YSBN6 *HTA2::mRFP1-NAT WHI5::sfGFP-KanMX*	[8]
YSBN6 Whi5-mCherry	YSBN6 WHI5::mCherry-BLE	[8]
YSBN10	YSBN10 *wild type*:<br>S288C-derived strain, MATa FY3 HO::HphMX4, ura3-52	[74],<br>from Steve Oliver lab,<br>Cambridge
YSBN10 Glycolytic biosensor	YSBN10 HO::pTEF7mut_CggRAla250, P_cggRO reporter plasmid	This study: transformation of YSBN10 with the P_cggRO reporter plasmid [47] (Addgene plasmid #124582) and with the DNA fragment containing pTEF7mut_CggRAla250 [47] (from Addgene plasmid #124585) as well as with a Cas9/sgRNA-expressing plasmid to target the fragment into HO
YSBN10 Control for glycolytic biosensor	YSBN10 P_cggRO reporter plasmid	This study: transformation of YSBN10 with the P_cggRO reporter plasmid [47] (Addgene plasmid #124582)

Supplementary Table 2. Primers employed in the construction of recombinant strains. This table summarizes primers employed for the amplification of DNA fragments used in plasmid assembly, primers for sequencing that checked the plasmid assembly, primers used for linearization of the plasmids before yeast transformation, primers used in the PCR verifying integration into genome and primers used in the sequencing of the resulting amplicons. Besides, we provide primers that were used to construct plasmids expressing sgRNAs for CRISPR/Cas9-assisted gene deletion. The underlined lowercase sequences represent the overhangs used in Gibson assembly. Genomic DNA is abbreviated with gDNA. The red underlined uppercase sequences target genes to be deleted via CRISPR/Cas9 system.

Primer	Sequence (5′ to 3′)	Template	Application
ATP3_cds_fwd	ttatgcttccgcggctcgtatgttgtgtggATAAAATTAAAATGCAGCTATTGAGAAC	YSBN6 gDNA	To create GA46 plasmid, linearize it and verify the genome integration of its sequence
ATP3_cds_rev	catgttatcctcctcgcccttgctcaccatTCCCAAAGAGGAAGCACC		
mCherry_IAA_nat_fwd.v2	ATGGTGAGCAAGGGCGAG	pG23A [44]	
mCherry_IAA_nat_rev.v2	AGCTTGCCTTGTCCCCGC		
ATP3_down_fwd	ccgggtgacccggcggggacaaggcaagctTAAAAAAATCACCCTGCATTG	YSBN6 gDNA	
ATP3_down_rev	gttccagtttggaacaagagtccactattaGCATACGCTTGGTAAAAAAC		
back2.v2_fwd	TAATAGTGGACTCTTGTTCCAAACTGGAAC	pG23A [44]	
back2.v2_rev	CCACACAACATACGAGCC		
seq_pr3	CCTTGAAGCGCATGAACTC	GA46 (sequencing, primers used separately)	
seq_pr4	CTGTCAAGGAGGGTATTCTGG		
ATP3_cds_fwd	See above	GA46 (linearization)	
ATP3_down_rev	See above		
ATP3_cds_fwd	See above	YSBN6 Atp3-mCherry gDNA (verification PCR)	
tCYC1_rev	CGTACGCTGAGCTGGATC		
seq_pr3	See above	YSBN6 Atp3-mCherry gDNA verification PCR amplicon (sequencing, primers used separately)	
tCYC1_rev	See above		
back_fwd	GGGTGTACAATATGGACTTC	pOsTIR1w/oGFP [44]	To create GA38 plasmid, linearize it and verify the genome integration of its sequence
back_rev	CGCCATTTTAAGTCCAAAGG		
pTEF1_fwd	ttgtgcctttggacttaaaatggcgCAGCTGGAATTCCACACC	pTEF:ATP [44]	
pTEF1_rev	caccccttggacatTTTAATAACCTAGGAAACTTAGATTAGATTG		
pH-tdGFP_fwd	ctaggttattaaaATGTCCAAGGGTGAAGAATTATTC	Addgene plasmid #74322 (pH-tdGFP, from Joerg Stelling)	
pH-tdGFP_rev	aagcttactcgagTTACTTGTATAATTCATCCATACCG		
tCYC1_fwd	attatacaagtaaCTCGAGTAAGCTTGGTACC	pTEF:ATP [44]	
tCYC1_rev	aagaggaagtccatattgtacacccCGTACGCTGAGCTGGATC		
seq_pr1	TGTTGTTTGCATTTATGATCCG	GA38 (sequencing, primers used separately)	
seq_pr2	CCGTCTTGTCCAAAGATCCTAAC		
Seq4_for	AATTATCCTGGGCACGAG	GA38 (linearization), YSBN6.pH-tdGFP.OsTir1.KanMX4 gDNA (verification PCR)	
Seq4_rev	ACTGTAAGATTCCGCCAC		
seq_pr2	See above	YSBN6.pH-tdGFP.OsTir1.KanMX4 gDNA (verification PCR)	
FL_UPST_rev	GCTATACCTGAGAAAGCAACC		
Can1_Cas9-fw	CTGTGTGGTTTCCGGGTGAGTCATAC	IMX585 [76] gDNA	To transform YSBN6 with the amplicon
Can1_Cas9-rv	CATTTGGTTCTAGGTTCGGGTGACG		
pROSmkrII-Phe-Alex-fw	TCATCAATAGGCACCTTCGTACGCTGCAGGATTAAGGGTTCTCGAGAGC	pUG66 [77]	To replace kanMX cassette in pROS13 [76] by the pAgTEF1-
pROSmkrII-Phe-Alex-rv	CATCGTCCTCTCGAAAGGTGGCATAGGCCATAGGTCTAGAGATCTGTTTAGC		

			ble-tAgTEF1 cassette and create pROS_phleo plasmid
TPS2_targetRNA FW	TGCGCATGTTTCGGCGTTCGAAACTTCTCCGCAGTGAAAGATAAATGATCATTTTGGAAACAAATTCTATGTTTTAGAGCTAGAAATAGCAAGTTAAAATAAG	n/a	These sequences were used to create pROS13-Tps2/Gsy1 plasmid employed in Tps2 and Gsy1 deletion
TPS2_repair oligo fw	CACGTTGAACAAGCAATAGAAAACCAAAATAACACTGCCTGTCACTATTTCTGTGCCGAAAACACCCTTTTTAACGAAATGGTTATGACTAGACAGACATCTTACGTCTTACTCCTTCAT		
TPS2_repair oligo rv	ATGAAGGAGTAAGACGTAAGATGTCTGTCTAGTCATAACCATTTCGTTAAAAAGGGTGTTTTCGGCACAGAAATAGTGACAGGCAGTGTTATTTTGGTTTTCTATTGCTTGTTCAACGTG		
TPS2_dg fw	ACAGGGAAATCGGCAGTGAG		
TPS2_dg rv	TACCTACCGCTGTTTCGACG		
GSY1_targetRNA FW	TGCGCATGTTTCGGCGTTCGAAACTTCTCCGCAGTGAAAGATAAATGATCCAATCTACAGTATTTTGATGGTTTTAGAGCTAGAAATAGCAAGTTAAAATAAG		
GSY1_repair oligo fw	ACAGCCTGGAAACCTGTGAAGAAAAAGAAAATAAACTCAGACGCAGCATCACAGCGAAGTAACCATCTTAACTTCTGCTAACATATCTTACTCTTTTAGCTGAGCATCAAATTAATTTTA		
GSY1_repair oligo rv	TAAAATTAATTTGATGCTCAGCTAAAAGAGTAAGATATGTTAGCAGAAGTTAAGATGGTTACTTCGCTGTGATGCTGCGTCTGAGTTTATTTTCTTTTTCTTCACAGGTTTCCAGGCTGT		
GSY1_dg fw	CTTGCCCAAAGAGGTTGCAC		
GSY1_dg rv	CTGCACCATTCTAAACGCGG		
TPS1_targetRNA FW	TGCGCATGTTTCGGCGTTCGAAACTTCTCCGCAGTGAAAGATAAATGATCTACAATAATAGCACCATTCAGTTTTAGAGCTAGAAATAGCAAGTTAAAATAAG	n/a	These sequences were used to create pROS_phleo-Tps1/Gsy2 plasmid employed in Tps1 and Gsy2 deletion
TPS1_repair oligo fw	AGCAACAAAGCAGGCTAACAAACTAGGTACTCACATACAGACTTATTAAGACATAGAACTTGAACCCGATGCAAATGAGACGATCGTCTATTCCTGGTCCGGTTTTCTCTGCCCTCTCTT		
TPS1_repair oligo rv	AAGAGAGGGCAGAGAAAACCGGACCAGGAATAGACGATCGTCTCATTTGCATCGGGTTCAAGTTCTATGTCTTAATAAGTCTGTATGTGAGTACCTAGTTTGTTAGCCTGCTTTGTTGCT		
TPS1_dg fw	TTCTTGAACAAGCACGCAGC		
TPS1_dg rv	ACGATAGCCTTGCATGGACC		
GSY2_targetRNA FW	TGCGCATGTTTCGGCGTTCGAAACTTCTCCGCAGTGAAAGATAAATGATCAATTTGTAAAAAAGACAAGAGTTTTAGAGCTAGAAATAGCAAGTTAAAATAAG		
GSY2_repair oligo fw	AGTGGTAGTTTTTTTGATAACTGTGATTGAAGTTTTGACTACCTCAGAGAAAAATTTTGAATCCTATGAGGATATAAACAGTATTAAAAAAATCTTACCATAAAGACATACGACATTTCG		
GSY2_repair oligo rv	CGAAATGTCGTATGTCTTTATGGTAAGATTTTTTTAATACTGTTTATATCCTCATAGGATTCAAAATTTTTCTCTGAGGTAGTCAAAACTTCAATCACAGTTATCAAAAAAACTACCACT		
GSY2_dg fw	AGGCCTTATGGGGTTCTTGC		
GSY2_dg rv	ACGCAAGAGGACTTCGCTAG		
sfGFP Forward	ATGTCCAAGGGTGAAGAGC	The plasmid HO-ptetO7-mCherry-sfGFP-KanMX-HO [91]	To remove mCherry from the plasmid. The PCR fragment
tet07-sfGFP Vector Reverse	CCCGAATTGATCCGGTA		

			was circularized via phosphorylation-ligation, resulting in pB.
Seq4_for	See above	pB	To linearize pB
Seq4_rev	See above		
fw_ugp1_cds	ttatgcttccgcggctcgtatgttgtgtggTGATCGAATCGAGCAATTTGG	YSBN6 gDNA	To create pUGP1.1 plasmid, linearize it and verify the genome integration of its sequence
rv_ugp1_cds	catgttatcctcctcgcccttgctcaccatATGTTCCAAGATTTGCAAATTACC		
fw_ugp1_down	ccgggtgacccggcggggacaaggcaagctTTTACTTTCAATTCTCCGTTAGGTT	YSBN6 gDNA	
rv_ugp1_down	gttccagtttggaacaagagtccactattaTGAACTCATATTGAGAAGACACA		
mCherry_IAA_nat_fwd.v2	See above	pG23A [44]	
mCherry_IAA_nat_rev.v2	See above		
back2.v2_fwd	See above	pG23A [44]	
back2.v2_rev	See above		
seq_pr3	See above	pUGP1.1 (sequencing, primers used separately), YSBN6 Ugp1-mCherry-AID gDNA verification PCR amplicon (sequencing)	
seq_pr4	See above		
fw_ugp1_cds	See above	pUGP1.1 (linearization), YSBN6 Ugp1-mCherry-AID gDNA (verification)	
rv_ugp1_down	See above		
Whi5-CDS For	ACGGACACGTTAGTATGCC	YSBN6 Whi5-mGFP gDNA (this strain was developed in our lab, mGFP is the same as in the strain KOY.TM6 Whi5-mGFP used in [8]); YSBN6 Ugp1-mCherry-AID gDNA (verification)	To get the cassette Whi5-mGFP-ZEO
Whi5-DN Rev	TGGTGCCGAGTCTGC		
Seq4_fwd	AATTATCCTGGGCACGAG	The plasmid pHO_pTEFmut7_CggR_R250A_ble [47]	To amplify the fragment HO_down–pTEFmut7–CggR–ter
TEF1rev1	CGAGGAGCCGTAATTTTTGC		

Supplementary Table 3. Conditions and strains used in the stop-and-respond experiments.

Perturbation type	Compound	Stock	Perturbation medium	Control medium	Strain	Light exposure
Inhibiting protein biosynthesis	Cycloheximide (CYH), Sigma (C7698)	5 mg/mL (water)	35 μg/mL CYH (13 mL of 2% glucose YNB + 91 μL of CYH stock)	13 mL of 2% glucose YNB + 91 μL of water	YSBN6.pTEF1-sfGFP	BF: 3V, 50 ms<br>GFP: 2%, 100 ms<br>RFP: 10%, 600 ms<br>NAD(P)H: 4%, 200 ms<br>$\delta t = 6$ min
Inhibiting fatty acid biosynthesis	Cerulenin (CER), Sigma (C2389)	10 mg/mL (DMSO)	25 μg/mL CER (13 mL of 2% glucose YNB + 32.5 μL of CER stock)	13 mL of 2% glucose YNB + 32.5 μL of DMSO	The same	The same. In the second replicate (dashed line in Fig. 2a) - without GFP channel.
Using the auxin-inducible degron system (Ugp1 depletion, control experiment)	1-naphthalene acetic acid (NAA), Sigma (N0640)	250 mM (water, KOH)	0.5 mM NAA (13 mL of 2% glucose YNB + 26 μL of NAA stock, pH not affected)	13 mL of 2% glucose YNB + 26 μL of water	YSBN6 Ugp1-mCherry-AID – for Ugp1 depletion, YSBN6.AIDcontrol – for the control experiment	BF: 3V, 50 ms<br>GFP: 3%, 300 ms<br>(RFP: 4%, 300 ms)*<br>NAD(P)H: 4%, 200 ms<br>$\delta t = 6$ min<br>*in Ugp1 depletion experiments but not in the control experiment

Supplementary Table 4. The time points of metabolic perturbation t_p used to collect the single cells' NAD(P)H derivative values for the analysis of the cell-cycle-dependent response in the stop-and-respond experiments. The decision for picking these particular time points comes from observing the population-averaged dynamics of NAD(P)H and growth rate (reflecting budding frequency). In the replicate experiments of the same perturbation type, we pick t_p in a consistent manner.

**Perturbation type**	t_p	**Comment**
Inhibiting protein biosynthesis	Immediately after the switch, *i.e.* $t_p = t_{switch}$	Abrupt drop of NAD(P)H signal and cessation of budding events happen immediately after the switch.
Inhibiting fatty acid biosynthesis	10 time points after the switch, *i.e.* $t_p = t_{switch} + 60\ min$	Abrupt decrease in budding frequency and change of NAD(P)H dynamics happen with a time delay after the switch (likely due to either adsorption of cerulenin to the microfluidic device's PDMS or prolonged build-up of the inhibitor in the cell).
Using the auxin-inducible degron system (Ugp1 depletion, control experiment)	3 time points after the switch, *i.e.* $t_p = t_{switch} + 18\ min$	NAD(P)H signal drops immediately after the switch, which is caused by an effect of NAA unrelated to the enzyme depletion since the drop is observed also in the strain lacking the degron. Therefore, we pick the later time point which corresponds to a significant drop of budding frequency caused by the enzyme depletion.

Supplementary Table 5. Model selection: oscillatory models explain the cell-cycle-resolved experimental data better and have higher predictive performance compared to linear models. For the data of every experiment, we independently run Gaussian process regression under two different models, described by two different kernels, and then compared two alternative measures of how well the data support each model. Model 1 assumes that the underlying function can "wiggle" but is smooth during the cell cycle. The kernel of model 1 is the sum of a Radial Basis Function (RBF) kernel (with optimized hyperparameter **l**, the length scale) and a white noise kernel (with the optimized hyperparameter **n**, the noise level). Model 1 was used in regressing the data in Fig. 1,2 and allowed us to conclude that protein biosynthesis activity has two waves per cell cycle whereas lipid and polysaccharide biosynthesis activities have one wave. On the other hand, model 2 assumes that the underlying function is linear (including a constant line) during the cell cycle, and it is therefore equivalent to performing Bayesian linear regression on the data [20]. The kernel of model 2 is the sum of Dot-Product kernel (with optimized hyperparameter **s**, the variance around the mean) and a white noise kernel (introduced above). For each model, we obtained the optimized hyperparameter values (shown on the table) via log-marginal likelihood maximization. We then compared the two models based on their log-marginal likelihood values (a Bayesian model selection criterion) and their leave-one-out cross-validation error (a frequentist criterion that approximates the prediction error of each model). Higher log-marginal likelihood indicates better support of the data for the respective model. The ratio of the marginal likelihood values of the oscillatory over the linear model (also known as the Bayes factor) quantifies the support of the data for the oscillatory versus the linear model. Bayes factor values higher than one (highlighted in green) indicate support for the oscillatory model. As an alternative approach to model selection, we also compared the oscillatory and linear models based on their estimated predictive performance. For this, we implemented leave-one-out cross-validation (LOO-CV) and presented in this table the leave-one-out log predictive probabilities for both models. The higher the leave-one-out log predictive probability, the higher the predictive performance of the model on unseen data. Predictive probability ratios larger than one (highlighted in green) indicate higher predictive performance of the oscillatory model. Gaussian process regression and calculations of the model selection measures were implemented via Python's module sklearn v.0.19.1, based on the theory presented in [20].

>>> next page >>>

Experiment	Gaussian process regression kernels with optimized hyperparameter values and *the respective shape of the fitted curve (posterior distribution) during the cell cycle*<br><br>Model 1 – oscillatory model<br>Model 2 – linear model	Log marginal likelihood value	Marginal likelihood ratio (oscillatory model/linear model)	Leave-one-out (LOO) log predictive probability (log pseudo-likelihood)	LOO predictive probability ratio (oscillatory model/linear model)
**Protein biosynthesis**, tetO$_7$-sfGFP microscopy, sfGFP maturation corrected data (Fig. 1a)					
Replicate 1, 38 cell-cycle traces	Model 1: 0.0714^2 · RBF(l=0.101) + WhiteKernel(n=0.0338), ***Two waves of biosynthesis***	**182.6630**	**6.8e+14**	**189.1535**	**1.2e+18**
	Model 2: 0.00316^2 · DotProduct(s=1.63e-05) + WhiteKernel(n=0.0384), *Constant biosynthesis*	148.5096		147.5242	
Replicate 2, 47 cell-cycle traces	Model 1: 0.0694^2 · RBF(l=0.112) + WhiteKernel(n=0.0425), ***Two waves of biosynthesis***	**120.8742**	**1.5e+11**	**126.4683**	**1.1e+14**
	Model 2: 0.00316^2 · DotProduct(s=1e-05) + WhiteKernel(n=0.0464), *Constant biosynthesis*	95.1256		94.1392	
Replicate 3, 43 cell-cycle traces	Model 1: 0.0486^2 · RBF(l=0.104) + WhiteKernel(n=0.0406), ***Two waves of biosynthesis***	**124.7708**	**1.4e+04**	**128.2267**	**1.2e+06**
	Model 2: 0.00316^2· DotProduct(s=1e-05) + WhiteKernel(n=0.0425), *Constant biosynthesis*	115.2303		114.2443	
**Protein biosynthesis**, NAD(P)H-based stop-and-respond method (Fig. 1d)					
Replicate 1, 304 cells	Model 1: RBF(l=17.6) + WhiteKernel(n=1.34), ***Two waves of biosynthesis***	**-484.0177**	**226.8399**	**-479.2882**	**110.9511**
	Model 2: DotProduct(s=0.000387) + WhiteKernel(n=1.41), *Constant biosynthesis*	-489.4420		-483.9973	
Replicate 2, 183 cells	Model 1: RBF(l=18) + WhiteKernel(n=1.12), ***Two waves of biosynthesis***	**-276.4838**	**15.6253**	**-272.7093**	**3.1740**
	Model 2: DotProduct(s=0.0012) + WhiteKernel(n=1.16), *Constant biosynthesis*	-279.2327		-273.8643	
**Lipid biosynthesis**, NAD(P)H-based stop-and-respond method (Fig. 2a)					
Replicate 1, 278 cells	Model 1: RBF(l=30) + WhiteKernel(n=0.296), ***One wave of biosynthesis***	**-233.3955**	**1.4e+04**	**-227.4371**	**4.6e+03**
	Model 2: DotProduct(s=0.194) + WhiteKernel(n=0.317), *Linearly increasing biosynthesis*	-242.9501		-235.8691	
Replicate 2, 145 cells	Model 1: RBF(l=30) + WhiteKernel(n=0.311), ***One wave of biosynthesis***	**-128.0863**	**4.3989**	-123.5675	
	Model 2: DotProduct(s=0.224) + WhiteKernel(n=0.315), ***Linearly increasing biosynthesis***	-129.5677		**-122.9742**	**0.5525**
**Polysaccharide biosynthesis**, NAD(P)H-based stop-and-respond method (Fig. 2c)					
Replicate 1, 71 cells	Model 1: RBF(l=36) + WhiteKernel(n=0.292), ***One wave of biosynthesis***	**-62.3645**	**441.9511**	**-59.0223**	**48.4584**
	Model 2: DotProduct(s=8.07e-05) + WhiteKernel(n=0.338), *Linearly increasing biosynthesis*	-68.4557		-62.9031	
Replicate 2, 132 cells	Model 1: RBF(l=36) + WhiteKernel(n=0.555), ***One wave of biosynthesis***	**-153.7453**	**1.5e+03**	**-150.2668**	**113.4818**
	Model 2: DotProduct(s=0.249) + WhiteKernel(n=0.604), *Linearly increasing biosynthesis*	-161.0570		-154.9985	

Supplementary Table 6. Parameter values used in the mathematical model describing cell mass during the cell cycle and converting the measured dimensionless biosynthetic activities into the rates expressed in absolute units. Polysaccharides are abbreviated with P.S., mitotic exit with – ME. The remaining parameter values are given along the description of the model in Supplementary Methods.

Parameters	Values	Sources
$b_{proteins}^{lower}$	1.4	[35,36]
b_{lipids}^{lower}	1.5	22 water molecules in two hydration shells per dipalmitoylphosphatidylcholine in a bilayer [37]
$b_{p.s.}^{lower}$	2.0	2 water molecules per a hydroxyl group of glucose under its high concentrations [38]
b_{DNA}^{lower}	2.1	20 water molecules per nucleotide [39]
b_{RNA}^{lower}	2.1	The hydration of RNA is expected to be higher than that of DNA due to the extra hydroxyl group
ω^{lower}, ω^{upper}	0.3, 0.4	[41,42]
$\xi_{proteins}^{m}$	0.505	From Table S3.1 of [40], we took values corresponding to the highest specific growth rate
ξ_{lipids}^{m}	0.057	
$\xi_{p.s.}^{m}$	0.234	
ξ_{DNA}^{m}	0.005	
ξ_{RNA}^{m}	0.105	
$x_j^m, \sigma_{x_j^m}\ for\ j \in \{proteins, RNA\}$	0.732, 0.047	The average and standard deviation of the ratio of the *mother-cell volume* regarding the *total volume* at ME in the 25 cell-cycle traces that were also used to get $V_m(t)$ and σ_V^{max} employed in the optimisation
$x_j^m, \sigma_{x_j^m}\ for\ j \in \{lipids, P.S.\}$	0.665, 0.036	The average and standard deviation of the ratio of the *mother-cell surface area* regarding the *total surface area* at ME in the 25 cell-cycle traces that were also used to get $V_m(t)$ and σ_V^{max} employed in the optimisation
$x_{DNA}^m, \sigma_{x_{DNA}^m}$	0.5, 0	Half of DNA remains in the mother cell at cytokinesis

Supplementary Table 7. New equations introduced into the thermodynamic-stoichiometric model [45] **instead of the original biomass equation.** The values in parentheses are reaction coefficients, [c] denotes the cytoplasmic location of a metabolite. Summing up these equations will result in the biomass equation used in [45], with the exception of (0.029) pi[c] and (0.02) so4[c] missing respectively as a product and a substrate due to the difficulty of assigning them to the separated equations of the biomass components. For the reactions of the six major biomass components and the biomass reaction, proton and charge coefficients were not estimated. Metabolite abbreviations are the same as in [45].

Protein reaction	(0.4588) ala-L[c] + (0.1607) arg-L[c] + (0.1017) asn-L[c] + (0.2975) asp-L[c] + (16.9650) atp[c] + (0.0066) cys-L[c] + (0.1054) gln-L[c] + (0.3018) glu-L[c] + (0.2904) gly[c] + (16.9650) h2o[c] + (0.0663) his-L[c] + (0.1927) ile-L[c] + (0.2964) leu-L[c] + (0.2862) lys-L[c] + (0.0507) met-L[c] + (0.1339) phe-L[c] + (0.1647) pro-L[c] + (0.1854) ser-L[c] + (0.1914) thr-L[c] + (0.0284) trp-L[c] + (0.1020) tyr-L[c] + (0.2646) val-L[c] <=> (16.9650) adp[c] + (16.9650) pi[c] + proteins[c]
Lipid reaction	(0.4536) accoa[c] + (0.4428) atp[c] + (0.0007) ergst[c] + (0.0053) g6p[c] + (0.0247) glyc3p[c] + (0.0051) met-L[c] + (0.82824) nadph[c] + (0.03304) o2[c] + (0.0122) ser-L[c] + (0.0015) zymst[c] <=> (0.4377) adp[c] + (0.0051) amp[c] + (0.4536) coa[c] + (0.0062) hco3[c] + (0.03778) h2o[c] + (0.0051) hcys-L[c] + (0.82824) nadp[c] + (0.4146) pi[c] + (0.0226) ppi[c] + lipids[c]
Polysaccharide reaction	(1.9427) atp[c] + (0.8079) f6p[c] + (1.1348) g6p[c] <=> (1.9427) adp[c] + (1.9427) ppi[c] + polysacch[c]
DNA reaction	(0.1040) atp[c] + (0.0036) damp[c] + (0.0024) dcmp[c] + (0.0024) dgmp[c] + (0.0036) dtmp[c] + (0.1040) h2o[c] <=> (0.1040) adp[c] + (0.1040) pi[c] + dna[c]
RNA reaction	(0.0460) amp[c] + (1.6380) atp[c] + (0.0447) cmp[c] + (0.0460) gmp[c] + (1.6380) h2o[c] + (0.0599) ump[c] <=> (1.6380) adp[c] + (1.6380) pi[c] + rna[c]
Storage reaction	(5.2096) atp[c] + (0.129625) glycogen[c] + (5.2096) h2o[c] + (0.0234) tre[c] <=> (5.2096) adp[c] + (5.2096) pi[c] + storage[c]
Biomass reaction	proteins[c] + lipids[c] + polysacch[c] + dna[c] + rna[c] + storage[c] <=>

Supplementary Table 8. Growth conditions among which cell-cycle-associated NAD(P)H oscillations were compared, and respective microscopy and analysis settings. MM denotes modified Verduyn's minimal medium. Microscopy channel names are followed by the percentage of the maximal light intensity and the exposure.

**Growth condition**: name, medium composition	**Microscopy and microfluidics details**: setup; objective; channels; time step δt; medium flow rate	**Window sizes of line fitting in LOWESS**: for detrending and smoothing
**1% Glu** (Fig. 5c,d and Extended Data Fig. 10), 1% (10 g/L) glucose MM	Setup 2A; 100x objective; BF (3V, 50 ms), NAD(P)H (15%, 150 ms), GFP (15%, 150 ms), RFP (15%, 150 ms); $\delta t = 5$ min; 3.6 μL/min	60 and 6 time points
**1% Glu (2)** (Extended Data Fig. 10), 1% (10 g/L) glucose MM	Setup 1A, 40x objective; BF (3V, 50 ms), NAD(P)H (15%, 100 ms), GFP (15%, 80 ms), RFP (15%, 200 ms); $\delta t = 2.5$ min; 4 μL/min	120 and 10 time points
**2% Pyr** (Fig. 5c,d and Extended Data Fig. 10), 2% (20 g/L) pyruvate MM	Setup 2A; 40x objective; BF (3V, 50 ms), NAD(P)H (8%, 80 ms), GFP (8%, 80 ms), RFP (8%, 200 ms); $\delta t = 10$ min; 3.6 μL/min	75 and 8 time points
**2% Pyr (2)** (Extended Data Fig. 10), 2% (20 g/L) pyruvate MM	Setup 2A; 40x objective; BF (3V, 50 ms), NAD(P)H (8%, 80 ms), GFP (8%, 80 ms), RFP (8%, 80 ms); $\delta t = 10$ min; 3.6 μL/min	75 and 8 time points
**1% Glu + LM** (Fig. 5c,d and Extended Data Fig. 10), 1% (10 g/L) glucose MM supplemented with 1% (0.01 L/L) Lipid Mixture 1 (Sigma, L0288)	Setup 1A; 100x objective; BF (3V, 50 ms), GFP (15%, 200 ms), NAD(P)H (15%, 200 ms); $\delta t = 5$ min; 3.6 μL/min	60 and 6 time points
**1% Glu + CSM** (Fig. 5c,d and Extended Data Fig. 10), 1% (10 g/L) glucose MM with 790 mg/L complete supplement mixture (CSM; Formedium, DCS0019) and 200 nM AlexaFluor 594 dye. The cells were switched to this condition in the microfluidic device from **1% Glu (2)**.	The same as in **1% Glu (2)**	The same as in **1% Glu (2)**<br>The data more than 4 hours after the switch is processed.
**1% Glu YPD** (Fig. 5c,d and Extended Data Fig. 10), 1% (10 g/L) glucose YPD	Setup 2A; 100x objective; BF (3V, 50 ms), GFP (15%, 150 ms), NAD(P)H (15%, 200 ms); $\delta t = 5$ min; 3.6 μL/min	60 and 6 time points
**Experiment with the switch to a microaerobic condition** (Fig.	Setup 2A; 40x objective; BF (3V, 50 ms), NAD(P)H (15%, 200 ms), GFP	60 and 6 time points

5a and Extended Data Fig. 9), 1% (10 g/L) glucose MM	(2%, 30 ms), RFP (25%, 250 ms); time step $\delta t = 5$ min; 3.6-4 μL/min	
**Experiment with the carbohydrate-storage mutant** (Fig. 5b), 1% (10 g/L) glucose MM	Setup 2A; 100x objective; BF (3V, 200 ms), NAD(P)H (20%, 200 ms); time step $\delta t = 5$ min; 4.8 μL/min	60 and 6 time points

REFERENCES

90. Warner JR: **The economics of ribosome biosynthesis in yeast**. *Trends Biochem Sci* 1999, **24**:437–440.

91. Huberts D: **The impact of metabolism on aging and cell size in single yeast cells**. Doctoral Thesis, University of Groningen 2015.
