# Supplementary material for: Temporal segregation of biosynthetic processes is responsible for metabolic oscillations during the budding yeast cell cycle
Source: Nat Metab. 2023 Feb 27;5(2):294–313. doi: 10.1038/s42255-023-00741-x (PMC9970877; doi:10.1038/s42255-023-00741-x)
Supplement: Supplementary file 2 — Reporting summary [file 42255_2023_741_MOESM2_ESM.pdf]

nature portfolio

Corresponding author(s): Matthias Heinemann

Last updated by author(s): Dec 23, 2022

Reporting Summary

Nature Portfolio wishes to improve the reproducibility of the work that we publish. This form provides structure for consistency and transparency in reporting. For further information on Nature Portfolio policies, see our Editorial Policies and the Editorial Policy Checklist.

Statistics

For all statistical analyses, confirm that the following items are present in the figure legend, table legend, main text, or Methods section.

n/a	Confirmed	
☐	☒	The exact sample size (*n*) for each experimental group/condition, given as a discrete number and unit of measurement
☐	☒	A statement on whether measurements were taken from distinct samples or whether the same sample was measured repeatedly
☒	☐	The statistical test(s) used AND whether they are one- or two-sided<br>*Only common tests should be described solely by name; describe more complex techniques in the Methods section.*
☒	☐	A description of all covariates tested
☒	☐	A description of any assumptions or corrections, such as tests of normality and adjustment for multiple comparisons
☐	☒	A full description of the statistical parameters including central tendency (e.g. means) or other basic estimates (e.g. regression coefficient) AND variation (e.g. standard deviation) or associated estimates of uncertainty (e.g. confidence intervals)
☒	☐	For null hypothesis testing, the test statistic (e.g. *F*, *t*, *r*) with confidence intervals, effect sizes, degrees of freedom and *P* value noted<br>*Give P values as exact values whenever suitable.*
☐	☒	For Bayesian analysis, information on the choice of priors and Markov chain Monte Carlo settings
☒	☐	For hierarchical and complex designs, identification of the appropriate level for tests and full reporting of outcomes
☐	☒	Estimates of effect sizes (e.g. Cohen's *d*, Pearson's *r*), indicating how they were calculated

Our web collection on statistics for biologists contains articles on many of the points above.

Software and code

Policy information about availability of computer code

Data collection	Nikon NIS Elements Advance Research v.4.51
Data analysis	ImageJ 1.49v and 1.52n; Python 3.6.2; Python modules: gdxpds 1.1.0, numpy 1.15.4, pandas 0.23.4, scikit-image 0.13.1, scipy 1.1.0, seaborn 0.9.0, statsmodels 0.10.1, matplotlib 3.3.4, sklearn 0.19.1, cobra 0.22.1; Gurobi 9.5.1; GAMS 31.1.1 and 31.2.0 (determining the parameters of the metabolic model), GAMS 39.3.0 (cell-mass model), geckopy 2.0.2<br><br>Code underlying the models and data analyses is availible at dataverse.nl via https://doi.org/10.34894/XPYC7Y

For manuscripts utilizing custom algorithms or software that are central to the research but not yet described in published literature, software must be made available to editors and reviewers. We strongly encourage code deposition in a community repository (e.g. GitHub). See the Nature Portfolio guidelines for submitting code & software for further information.

Data

Policy information about availability of data

All manuscripts must include a data availability statement. This statement should provide the following information, where applicable:

- Accession codes, unique identifiers, or web links for publicly available datasets
- A description of any restrictions on data availability
- For clinical datasets or third party data, please ensure that the statement adheres to our policy

Data extracted from microscopy imaging, analysis- and modelling-related data are available at https://doi.org/10.34894/XPYC7Y. Microscopy raw data can be obtained from Matthias Heinemann.

Human research participants

Policy information about studies involving human research participants and Sex and Gender in Research.

Reporting on sex and gender	We did not perform human research.
Population characteristics	We did not perform human research.
Recruitment	We did not perform human research.
Ethics oversight	We did not perform human research.

Note that full information on the approval of the study protocol must also be provided in the manuscript.

Field-specific reporting

Please select the one below that is the best fit for your research. If you are not sure, read the appropriate sections before making your selection.

☒ Life sciences ☐ Behavioural & social sciences ☐ Ecological, evolutionary & environmental sciences

For a reference copy of the document with all sections, see nature.com/documents/nr-reporting-summary-flat.pdf

Life sciences study design

All studies must disclose on these points even when the disclosure is negative.

Sample size	All experiments performed in this study are microscopy-based experiments. In experiments with dynamic addition of inhibitors, auxin or glucose analogue and in subsequent single-cell analyses, we traced the maximal number of cells cultivated in the microfluidic device, with these cells reliably segmented and having smooth trajectories of studied variables according to visual inspection. We consider the sample sizes sufficient to draw reliable conclusions as we observed the reported average patterns in replicate experiments and in experiments using alternative methods. In experiments with constant growth conditions and in subsequent single-cell analyses, we traced either the maximal number of cells in the microfluidic device or a smaller than maximal number of cells, which was enough to obtain reliable average patterns such that these patterns reflect individual single-cell trajectories. For the experiments with the oxygen-level perturbation and with cultivation of carbohydrate-metabolism mutant in constant growth conditions, we demonstrated the dynamics of studied variables in the continuous trajectories of several representative cells as it was sufficient to draw respective conclusions. We indicate the exact number of analyzed cells for each experiment.
Data exclusions	We performed visual inspection of cell segmentation and cell tracking quality both on the level of raw time-lapse microscopy images and on the level of single-cell trajectories of cell volume and fluorescence. We identified obvious artefacts generated by wrong cell segmentation, tracking and focus shifts, which result in abrupt jumps in measured mother or daughter cell volumes and fluorescence. We discarded single-cell trajectories or individual data points in single-cell trajectories affected by such artefacts. In a fraction of cells, we were not able to reliably detect the timing of mitotic exit and START due to noisy Whi5 signal, thus, we did not use the data of those cells in analyses requiring the timing of the cell-cycle events. We also excluded data from cells with extreme durations of the cell cycle or parts of the cell cycle (described in detail in Methods). These data-exclusion criteria were preestablished. The data analysis code (namely, Jupyter Notebooks) that we made available via dataverse.nl (https://doi.org/10.34894/XPYC7Y) documents all data exclusion cases.
Replication	We replicated the following perturbation experiments twice: cycloheximide-based stop-and-respond experiments, cerulenin-based stop-and-respond experiments, Ugp1-depeltion stop-and-respond experiments, glucose-analogue-uptake experiments. The control experiment for the Ugp1-depeltion stop-and-respond experiments was performed once. The experiment to trace the production rate of tetO7-controlled sfGFP during the cell cycle was repeated three times. We present the cell-cycle-resolved dynamics of cell volume and cell surface from one experiment as growth conditions were not perturbed in this experiment, and there is low variability in the dynamics of individual cell-cycle traces. The experiment with the glycolytic-flux biosensor was performed once as growth conditions were not perturbed in this experiment, the finding was reproduced with an alternative method (glucose analogue uptake), and a control experiment without the glycolytic-flux-sensing moiety of the sensor was carried out. The oxygen-level perturbation experiment was performed once as it was sufficient to draw the respective conclusion. The experiments to observe NAD(P)H oscillations on YPD medium and glucose minimal media containing complete

	supplement mixture and lipid mixture were performed once. The experiments to observe NAD(P)H oscillations on glucose minimal medium without other carbon-source supplements and on pyruvate minimal medium were performed twice. Critical findings were reproduced using alternative experimental methods. Replicate cell-cycle-resolved patterns of protein, lipid and polysaccharide synthesis were used as an input of the cell-mass model and metabolic model.
Randomization	Randomization was not applied since we did not expect the influence of the order of the experiments on their outcome, as judged by our previous experience and publications in the field. Experiments with yeast cells were performed under controlled conditions and with one genetic background.
Blinding	Blinding was not possible in this study as the experimentalists designed experiments and analyzed respective data.

Reporting for specific materials, systems and methods

We require information from authors about some types of materials, experimental systems and methods used in many studies. Here, indicate whether each material, system or method listed is relevant to your study. If you are not sure if a list item applies to your research, read the appropriate section before selecting a response.

Materials & experimental systems

n/a	Involved in the study
☒	☐ Antibodies
☐	☒ Eukaryotic cell lines
☒	☐ Palaeontology and archaeology
☒	☐ Animals and other organisms
☒	☐ Clinical data
☒	☐ Dual use research of concern

Methods

n/a	Involved in the study
☒	☐ ChIP-seq
☒	☐ Flow cytometry
☒	☐ MRI-based neuroimaging

Eukaryotic cell lines

Policy information about cell lines and Sex and Gender in Research

Cell line source(s)	Saccharomyces cerevisiae strains:<br>YSBN6 wild type (S288C background), Canelas et al., 2010<br>YSBN6 ATP3::mCherry-AID-NatMX HO::pTEF1-pH-tdGFP-pADH1-OsTIR1-KanMX4, This study<br>YSBN6 HO::pTEF1-pH-tdGFP-pADH1-OsTIR1-KanMX4, This study<br>YSBN6 Can1Δ::cas9-natNT2, This study<br>YSBN6 ΔTps1ΔGsy2 Can1Δ::cas9-natNT2, This study<br>YSBN6 ΔTps1ΔTps2ΔGsy1ΔGsy2 Can1Δ::cas9-natNT2, This study<br>YSBN6 HO::KanMX4-pTEF1-mGFP-AID-tCYC-pADH1-AtTIR-tADH1, Papagiannakis et al., 2017<br>YSBN6 HO::tetO7-sfGFP-KanMX WHI5::mCherry-BLE, This study<br>YSBN6 HO::pTEF1-sfGFP-KanMX WHI5::mCherry-BLE, Litsios et al., 2019<br>YSBN6 UGP1::mCherry-AID-NatMX WHI5::mGFP-ZEO HO::ADH1p-OsTIR1-KanMX4, This study<br>YSBN6 WHI5::mGFP-ZEO HO::ADH1p-OsTIR1-KanMX4, This study<br>YSBN6 HTA2::mRFP1-NAT WHI5::sfGFP-KanMX, Litsios et al., 2019<br>YSBN6 WHI5::mCherry-BLE, Litsios et al., 2019<br>YSBN10 wild type (S288C background), Canelas et al., 2010<br>YSBN10 HO::pTEF7mut_CggRAla250, P_cggRO reporter plasmid, This study<br>YSBN10 P_cggRO reporter plasmid, This study
Authentication	Authentication of strains which were not generated in this study was done on the basis of their expected phenotype
Mycoplasma contamination	Given that only yeast strains were used in this study, no testing for mycoplasma contamination was performed
Commonly misidentified lines<br>(See ICLAC register)	No commonly misidentified cell lines were used
